# Genetic interactions and functional analyses of the fission yeast *gsk3* and *amk2* single and double mutants defective in TORC1-dependent processes

**DOI:** 10.1038/srep44257

**Published:** 2017-03-10

**Authors:** Charalampos Rallis, StJohn Townsend, Jürg Bähler

**Affiliations:** 1 Research Department of Genetics, Evolution & Environment and UCL Institute of Healthy Ageing, University College London, Gower Street, WC1E 6BT, London, UK

## Abstract

The Target of Rapamycin (TOR) signalling network plays important roles in aging and disease. The AMP-activated protein kinase (AMPK) and the Gsk3 kinase inhibit TOR during stress. We performed genetic interaction screens using synthetic genetic arrays (SGA) with *gsk3* and *amk2* as query mutants, the latter encoding the regulatory subunit of AMPK. We identified 69 negative and 82 positive common genetic interactors, with functions related to cellular growth and stress. The 120 g*sk3*-specific negative interactors included genes functioning in translation and ribosomes. The 215 *amk2-*specific negative interactors included genes functioning in chromatin silencing and DNA damage repair. Both *amk2-* and *gsk3-*specific interactors were enriched in phenotype categories related to abnormal cell size and shape. We also performed SGA screen with the *amk2 gsk3* double mutant as a query. Mutants sensitive to 5-fluorouracil, an anticancer drug are under-represented within the 305 positive interactors specific for the *amk2 gsk3* query. The triple-mutant SGA screen showed higher number of negative interactions than the double mutant SGA screens and uncovered additional genetic network information. These results reveal common and specialized roles of AMPK and Gsk3 in mediating TOR-dependent processes, indicating that AMPK and Gsk3 act in parallel to inhibit TOR function in fission yeast.

The nutrient-responsive Target of Rapamycin (TOR) signalling pathway regulates fundamental cellular processes such as growth in response to environmental or physiological factors. In the fission yeast, *Schizosaccharomyces pombe*, two Tor kinases (as opposed to a single kinase in mammals) function within two protein complexes, TORC1 and TORC2^1^. Tor2 is part of TOR Complex 1 (TORC1) and positively regulates cellular growth, while Tor1 predominates in the antagonistically acting TOR Complex 2 (TORC2)^2^,^3^. We have previously demonstrated that the fission yeast TOR pathway can be pharmacologically inhibited by the combined action of rapamycin and caffeine; TORC1 inhibition mimics the effects of nitrogen starvation and results in increased chronological lifespan (CLS)^4^.

The duration and intensity of TORC1 activation depends on the abundance and quality of different nutrients. Genetic studies in fission yeast have demonstrated that the TSC1-TSC2 complex is an negative regulator upstream of TORC1^1^,^5^. Effectors downstream of TORC1 have been extensively characterised, including the AGC kinases Psk1, Sck1 and Sck2 that phosphorylate a variety of regulatory proteins to promote processes such as protein synthesis^6^,^7^. For example, a major regulator of protein translation, the translation initiation factor eIF2α^8^,^9^, can be phosphorylated by the Gcn2 kinase to inhibit translation; in nitrogen-rich conditions, Gcn2 is inactivated in a TORC1-dependent manner, thus providing a mechanism for TORC1 to promote translation in the presence of nutrients^9^,^10^.

The AMP-activated protein kinase (AMPK) can detect decrease in cellular energy via AMP levels; in fission yeast, AMPK consists of Ssp2, Amk2 and Cbs2^11^. AMP is an allosteric activator of AMPK: binding to the βγ regulatory subunit (Amk2 and Cbs2) renders the α subunit (Ssp2) a better target for the stress-responsive kinase Ssp1, and Ssp2 phosphorylation activates AMPK, thus promoting catabolic and inhibiting anabolic processes^12^. AMPK is functionally linked to TORC1-dependent processes: the budding yeast Ssp2 ortholog, Snf1, mediates the phosphorylation and activation of Gcn2 in response to nutritional stress, leading to phosphorylation of eIF2α and a global reduction in translation^8^.

Gsk3 is a conserved kinase that inhibits the canonical Wnt pathway^13^ and has clinical significance^14^. However, its role in unicellular eukaryotes remains poorly characterized. We have shown that *gsk3-*mutant cells are resistant to TORC1 inhibition by caffeine and rapamycin and feature a shortened CLS when glucose is depleted^15^. Human GSK3 inhibits AMPK by interacting with the AMPK β subunit while phosphorylating and inactivating the α subunit. Disruption of GSK3 function within the AMPK complex promotes high AMPK activity and cellular catabolic processes even under anabolic conditions, indicating that GSK3 acts as a critical sensor for anabolic signalling to regulate AMPK^16^. To investigate the cellular roles and relationships of AMPK and Gsk3 in fission yeast, we have applied classical and genome-wide genetic approaches. We show that AMPK and Gsk3 are functionally implicated in the same TOR-dependent processes, involving both common and specific genetic interactors.

## Materials and Methods

### Fission yeast strains and media

Cell cultures were grown in YES liquid medium at 32 °C, with shaking at 130 rpm. Deletion mutants were generated as previously described^17^. For wild-type the *972*− strain was used while for growth, stress and lifespan assays the following strains were used: *h*− *gsk3::kanMX6, h*− *gsk3::natMX6, h*− *gsk3::hphMX6, h* + *amk2::natMX6, h*− *amk2::natMX6 gsk3::hphMX6, h* + *ssp2::kanMX6, h*− *ssp2::kanMX6 gsk3::natMX6, h*− *ssp2::kanMX6 gsk3::hphMX6, h* + *ssp1::kanMX6* and *h*− *ssp1::kanMX6 gsk3::hphMX6*.

### Growth assays

Growth curves were generated using the BioLector microfermentation system (m2p-biolabs) as previously described^4^. Caffeine and rapamycin were used at concentrations of 100 mM and 100 ng mL^−1^, respectively while hydrogen peroxide at a concentration of 0.5 mM. Growth kinetics were analysed in R using the grofit package^18^ in order to calculate the time taken for biomass to increase by 25%. This is used as a proxy for caffeine and rapamycin resistance^15^. In the case of hydrogen peroxide treatment the lag phase difference between untreated and treated cells has been used as a proxy for resistance^4^.

### Chronological lifespan assays

Cells were grown in YES to stationary phase and assays were performed as previously described^4^, using CFU counting with data normalised for the day when cells reached stationary phase (Day 0). Error bars represent standard deviations from three independent biological repeats, each one consisting of three technical repeats.

### SGA analyses

SGAs were performed using the Bioneer v5.0 haploid library^19^ essentially as described previously, with each interaction examined in quadruplicate and deriving from independent spore mass pick up^15^,^20^. Query strains were the following: *h*− *gsk3::hphMX6, h*− *amk2::natMX6, h*− *gsk3::hphMX6 amk2::natMX6* and control query *h*− *ade6::natMX6*^15^. Colony size was used as a proxy for double/triple mutant fitness. Colony size measurements were obtained using the gitter package in R^21^ or the Spotsizer tool^22^,^23^. In order to exclude potentially spurious interaction values as a result of linkage, library mutants which fell within 100 kb distance of query mutations were excluded from the respective datasets. Any library mutants for which colony size of the *ade6* double mutant was less than 100 pixels were deemed to be present in too little amount for accurate interaction values to be calculated, and were hence excluded from the dataset. Interaction values were calculated as follows (in-house SGA analysis tool, Townsend *et al*., under preparation): colony sizes were normalised to the plate median in order to control for the effect of the query mutation and plate-specific effects; colony sizes were also normalised for column- or row-specific effects. A median filter was applied to identify and correct for local spatial effects. Medians of colony sizes of the *gsk3, amk2* and *gsk3 amk2* SGAs were normalised with respect to the *ade6* SGA (which represents fitness of library single mutants as query does not alter fitness of library) to calculate the value of genetic interactions between query mutants and library mutants. Interactions with high within-replicate variability were excluded. Finally, the logarithms in base 10 of the interaction were used for interaction scores. For practical reasons, +2 and −2 were taken to be the maximum and minimum values for interaction scores, respectively.

### Enrichment analyses of interactions

Cut-offs for interactions with *gsk3* and *amk2* were −0.15 and + 0.15. Enrichment analyses were performed using the AnGeLi tool^24^, for which p-values were corrected for multiple tests according to FDR. Enrichment analysis was conducted by comparing lists of interacting genes to all genes in the dataset (Bioneer v5.0 collection strains).

## Results and Discussion

### Gsk3 suppresses cellular functions of TORC1

**Figure 1:**
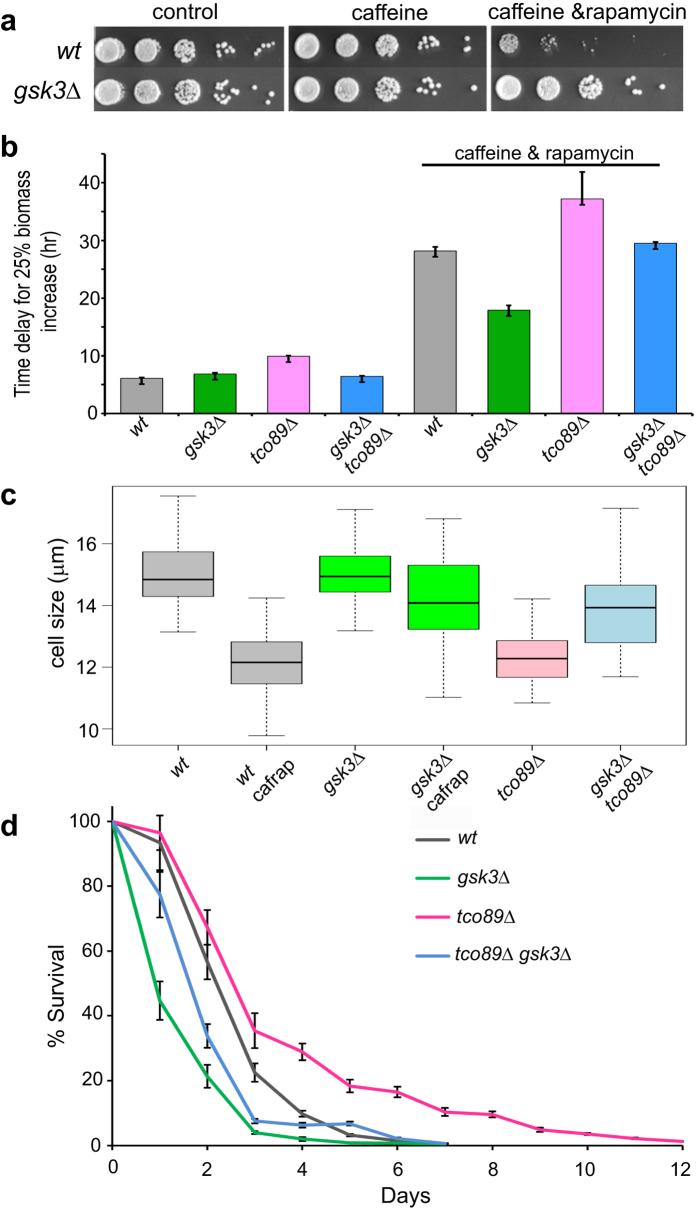
Deletion of *gsk3* represses phenotypes associated with TORC1 inhibition. (**a**) Spot tests of wild-type (*wt*) and *gsk3Δ* cells in the presence or absence of drugs as indicated. (**b**) Graph showing times to reach 25% of maximum cell mass increase for different strains with or without drug treatment as indicated. (**c**) Cell size upon division for different strains as indicated; cafrap: caffeine and rapamycin treatment. (**d**) CLS assays of different strains as indicated. Error bars represent standard deviations of three independent biological repeats.

Fission yeast *gsk3Δ* cells are resistant to TORC1 inhibition by combined treatment with caffeine and rapamycin (Fig. 1a)^15^. To gain insight into functional relationships of Gsk3 within the TOR signalling network, we examined how Gsk3 affects phenotypes related to TORC1 inhibition. TORC1 was inhibited by deletion of *tco89* (a non-essential subunit of TORC1)^2^ and/or by combined treatment with caffeine and rapamycin^4^. The time taken for biomass to increase by 25% for normal or mutant cells grown in the absence or presence of caffeine and rapamycin was used for growth assessment^15^. The severity of TORC1 inhibition negatively correlated with cell growth, with combined deletion of *tco89* and drug treatment representing the most severe inhibition (Fig. 1b). Deletion of *gsk3* was able to rescue the growth defect associated with a mild TORC1 inhibition (*tco89Δ* cells) as well as the phenotype of severe TORC1 inhibition (Fig. 1b, combination of *tco89Δ* and drug treatment). Furthermore, deletion of *gsk3* largely rescued the reduced cell size at division triggered by both pharmacological and genetic TORC1 inhibition (Fig. 1c). Cells deleted for both *gsk3* and *tco89* also showed a short CLS, similar to the *gsk3Δ* single mutant^15^ (Fig. 1d). Together, these results indicate that fission yeast Gsk3 counteracts TORC1 function. This could be achieved either upstream of TORC1 by reducing TORC1 activity itself and/or downstream of TORC1 by inhibiting cell growth. These two scenarios are not mutually exclusive, because Gsk3 is an upstream regulator affecting the TSC2 inhibitor of TORC1^25^, which is conserved in fission yeast^26^ and has also been shown to function downstream of TORC1^27^.

### Gsk3 and AMPK act in parallel to inhibit TORC1-dependent processes

**Figure 2:**
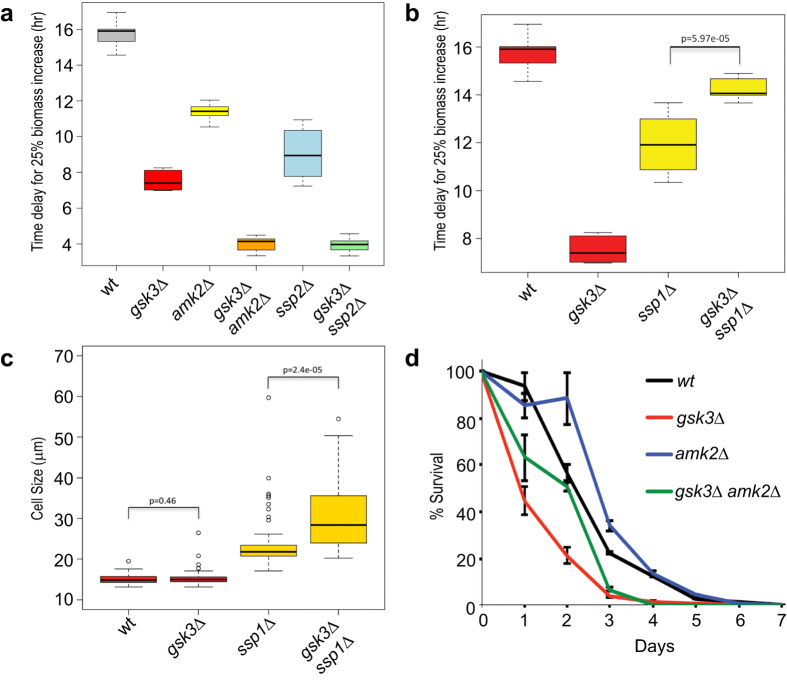
Deletions of *gsk3* and *amk2* additively repress phenotypes associated with TORC1 inhibition. (**a**) Graph showing the times to achieve 25% increase in cell mass for wild-type (*wt*) and mutant cells as indicated under caffeine and rapamycin treatment. (**b**) Times to achieve 25% increase in cell mass as in panel A, with strains as indicated. (**c**) Cell size upon division for strains shown in panel B. (**d**) CLS assays for different strains as indicated. Error bars indicate standard deviations from three biological repeats.

Previous studies have shown that AMPK negatively regulates TORC1-related processes^28^. We therefore tested the response of cells lacking *ssp2 (AMPKα*) or *amk2 (AMPKβ*) for caffeine and rapamycin resistance. Like *gsk3Δ* cells, deletion of *amk2* or *ssp2* resulted in cells whose growth was less inhibited in the presence of caffeine and rapamycin compared to wild-type cells (Fig. 2a). Furthermore, combined deletion of *gsk3* and either *amk2* or *ssp2* resulted in an even lower inhibition of cell growth by caffeine and rapamycin than shown by any of the three single mutants. This additive resistance to caffeine and rapamycin of the double mutants suggests that Gsk3 and AMPK operate in parallel to negatively regulate TORC1-dependent processes.

Ssp1 is a direct upstream activator of AMPK and *ssp1* deletion results in loss of AMPK activity^28^,^29^. We therefore expected that the phenotype of *ssp1Δ* cells will mimic that of *amk2Δ* and *ssp2Δ* cells. Indeed, *ssp1Δ* cells showed lowered inhibition of growth by caffeine and rapamycin (Fig. 2b). However, the *ssp1Δ gsk3Δ* double mutant cells showed higher inhibition of cell growth by caffeine and rapamycin, in stark contrast to the *amk2Δ gsk3Δ* and *ssp2Δ gsk3Δ* double mutants. This result suggests that Ssp1 is required for the resistance of *gsk3Δ* cells to caffeine and rapamycin. To further explore the relationship between Ssp1 and Gsk3, we measured cell sizes at division of *ssp1Δ* and *ssp1Δ gsk3Δ* mutants. As previously reported^28^, *ssp1Δ* cells were elongated and the *ssp1Δ gsk3Δ* double mutant cells showed an even greater increase in cell length (Fig. 2c). Thus, deletion of *gsk3* exacerbates the elongated phenotype of *ssp1Δ* cells. The PInt tool^30^ predicts a physical interaction between Gsk3 and Ssp1, implying that Gsk3 can operate upstream of AMPK. However, using co-immunoprecipitation assays, we found no evidence for any direct interaction between Gsk3 and Ssp1 (data not shown). Together, these results reveal a functional relationship between Gsk3 and Ssp1 that is distinct from the relationship between Gsk3 and AMPK components.

To further investigate relationships between Gsk3 and AMPK, we performed CLS assays. As reported before^15^, *gsk3Δ* cells showed a reduced CLS in rich medium (Fig. 1d), whilst the CLS of *amk2Δ* cells was similar to that of wild-type cells (Fig. 2d). However, the CLS of *amk2Δ gsk3Δ* double mutant cells was reduced compared to wild-type cells: initially the lifespan of the double mutant was intermediate of the single mutants, whilst in later time points was severely reduced. Together, these findings suggest that Gsk3 can act independently of AMPK in different contexts. In mammalian cells, GSK3 and AMPK signalling are integrated at TSC2, which then inhibits mTORC1^25^ and the relationship between AMPK, the TSC1/2 complex and TORC1 is conserved in fission yeast^28^. It is not known whether Gsk3 and Tsc2 functionally interact in fission yeast. AMPK is linked to transcriptional responses, such as the nuclear accumulation of the transcription factor Ste11, which triggers sexual differentiation in response to nitrogen starvation^29^. Taken together, these results suggest that Gsk3 and AMPK largely act in parallel to mediate cellular responses related to TORC1 signalling.

### Genetic interactions of *gsk3* and *amk2*

Genetic (or epistatic) interactions reveal functional relationships between genes and pathways. Negative genetic interactions suggest compensatory, redundant pathways or protein complexes, while positive genetic interactions can reflect functions within the same pathway^31^, but most genetic interactions reveal broad phenotypic connections between functional modules or biological processes^32^. We conducted synthetic genetic array (SGA) analyses^15^,^20^ to identify genome-wide genetic interactions of *gsk3* and *amk2*. The query strains, which all showed equal fitness when grown on YES (data not shown), were crossed against all non-essential mutants in a deletion library^19^.

**Figure 3:**
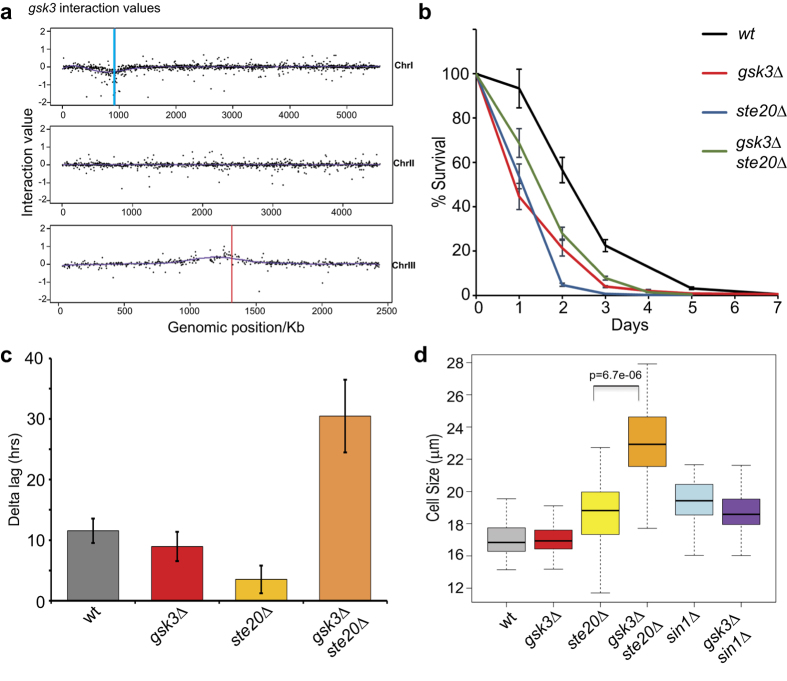
Genetic interactions of *gsk3* and *ste20* mutants. (**a**) Physical mapping along the three chromosomes of mutants that genetically interact with *gsk3*. Blue and red lines indicate the *gsk3* and *ade6* loci, respectively. (**b**) CLS assays comparing wild-type (*wt*) and mutant cells as indicated. Error bars indicate standard deviations from three biological repeats. (**c**) Comparisons of lag phase with oxidative stress minus lag phase without oxidative stress (Delta lag) in hrs for different strains as indicated. (**d**) Cell size upon division for different single and double mutants as indicated.

We analysed genes showing positive or negative interactions with *gsk3Δ* for enrichments with AnGeLi^24^, using the non-essential genes present in the deletion library as a reference^19^. The data for all genetic interactions are presented in Supplemental Table 1. As expected, a bias of negative interactions with *gsk3* was evident around the genomic locus of *gsk3,* reflecting genetic linkage (blue line in Fig. 3a). For the same reason, a bias towards positive interactions was evident around the *ade6* locus, because *ade6* SGAs were used for control and normalisation purposes (red line in Fig. 3a).

Negative interactions with *gsk3* were enriched for Gene Ontology (GO) categories related to protein translation, histone modification, endosome to vacuole transport, protein metabolic process and protein modification by small protein conjugation or removal, i.e. ubiquitination or ubiquitin-proteasome system (UPS). Further investigations will be required to define how and under which conditions Gsk3 may be functionally associated with the UPS. As Gsk3 suppresses TORC1-related phenotypes (Fig. 1), a plausible mechanism is through regulation of protein synthesis, which is a major determinant of CLS^4^,^15^,^33^. Positive interactions were enriched for genes related to abnormal cell shape, decreased population growth and stress (Supplemental Table 2).

Among the positive interactors of *gsk3* was s*te20* which encodes the Rictor homologue, a core component of TORC2^2^. To validate this genetic interaction, we performed CLS assays. While *ste20* mutant cells were even more short-lived than *gsk3* mutant cells, the double mutant cells, albeit short-lived compared to wild-type, showed a somewhat longer CLS than either single mutant (Fig. 3b). Thus, *gsk3* and s*te20* show a positive genetic interaction also in the phenotypic context of CLS. TORC2 is required for various stresses including oxidative stress^34^. Both *gsk3* and s*te20* single mutants showed increased resistance to hydrogen peroxide (Fig. 3c), exhibiting a shorter lag-phase extension between treated and untreated conditions compared to wild-type^4^,^15^. Surprisingly, the double mutant was sensitive to the same oxidative-stress treatment (Fig. 3c). As stress and growth programs are antagonistic^35^, this result of decreased stress resistance of the double mutant is consistent with the improved growth recorded in the SGA screens. The *ste20Δ* cells were larger upon division compared to wild-type, and this phenotype was further enhanced in the *gsk3Δ ste20Δ* double mutant (Fig. 3d). In contrast, the phenotype of the elongated *sin1Δ* mutant cells, which are also defective in TORC2, was diminished in the *gsk3Δ sin1Δ* double mutant. These results reveal diverse, complex and antagonistic genetic interactions of *gsk3* with genes encoding TORC2 components. We conclude that the relationship between Gsk3 and TORC2 is multifaceted, and it is possible that TORC2 activities are determined by several distinct thresholds of activation.

**Figure 4:**
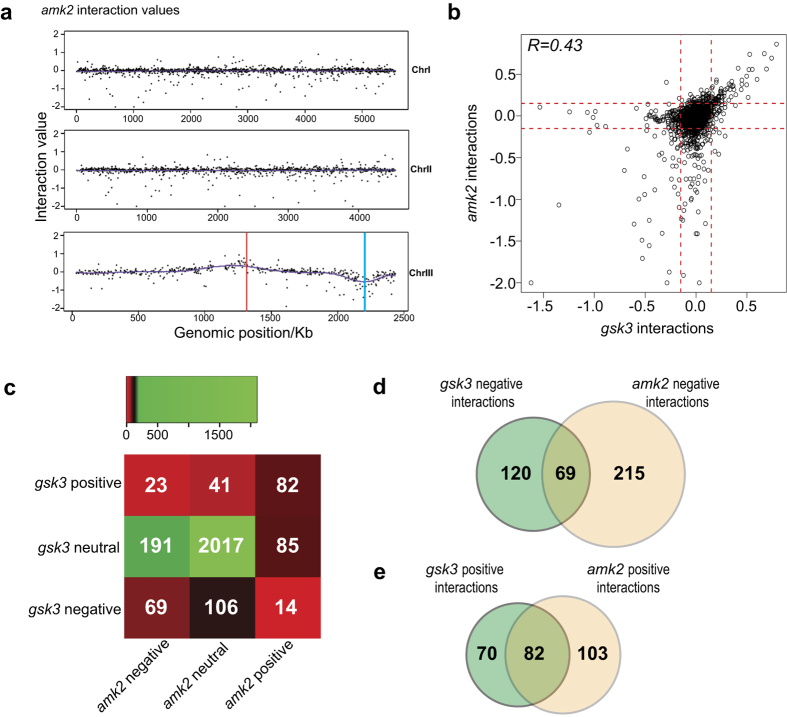
Relationships of genetic interactions of *gsk3* and *amk2*. (**a**) Physical mapping along the three chromosomes of mutants that genetically interact with *gsk3*. Blue and red lines indicate the *amk2* and *ade6* loci, respectively. (**b**) Distribution of *gsk3* interactions with respect to *amk2* interactions. Red lines indicate cut-offs used for positive and negative interactions. (**c**) Genetic interactions were classified as positive (interaction value i ≥0.15) negative (i ≤ 0.15) or neutral (−0.15 < i < 0.15) for both *gsk3* and *amk2*. The numbers of genes for each category are indicated. (**d**) Venn diagram showing overlaps of negative genetic interactions for *gsk3* and *amk2*. (**e**) Venn diagram showing overlaps of positive genetic interactions for *gsk3* and *amk2*.

As expected, the SGA screens with *amk2* showed a negative interaction bias around the *amk2* locus on chromosome 2 (blue line, Fig. 4a) and a positive interaction bias around the *ade6* locus (red line, Fig. 4a). The negative interactions with *amk2* were enriched for phenotypes^36^ such as sensitivity to chemical during vegetative growth (FYPO:0000127, 0002683), abnormal cellular physical quality (FYPO: 0004638), and chromatin silencing at centromere (FYPO: 0002346, 0003412) (Supplemental Table 2). Genes showing positive genetic interactions were enriched for phenotypes related to stress and abnormal cell shape. Although there was no significant enrichment in GO terms, many interacting genes were related to amino acid metabolism, lipid homeostasis, and DNA repair. Notably, loss of AMPK reduces the activity of enzymes responsible for UVB-induced DNA repair in mammalian cells^37^.

The genetic interactions of *gsk3* and *amk2* were moderately correlated with each other (Fig. 4b). Genes that either positively or negatively interacted with both *gsk3* and *amk2* were more abundant than genes that interacted only with one of the two genes (Fig. 4c). There were 69 common negative and 82 common positive genetic interactions (Fig. 4d). These common gene lists showed no functional enrichments in AnGeLi^24^. We conclude that Amk2 and Gsk3 play roles in the same phenotypes (such as stress and abnormal cell shape) through both common and specific interactors.

### Triple mutant SGA: providing additional insights into Gsk3 and Amk2 functions

**Figure 5:**
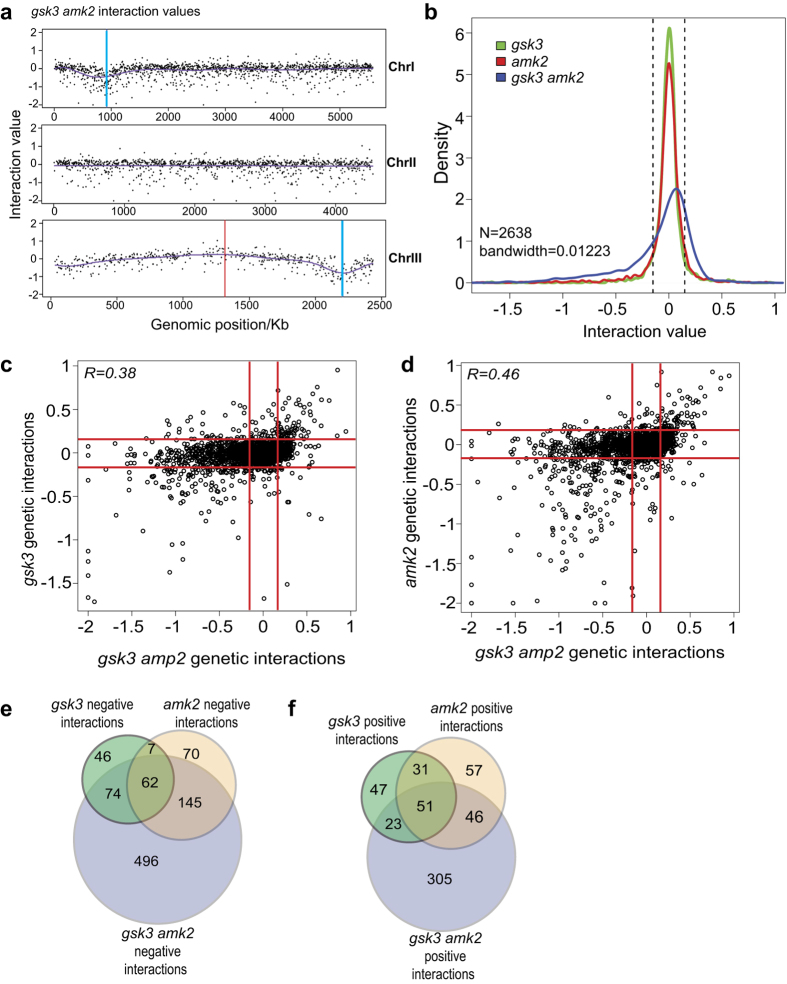
Triple mutant SGA using *gsk3 amk2* as query. (**a**) Physical mapping along the three chromosomes of mutants that genetically interact with *gsk3 amk2* double mutant. Blue lines on Chromosomes I and III represent the genetic loci of *gsk3* and *amk2,* respectively, while the red line indicates the *ade6* locus. (**b**) Distributions of genetic interaction values for the three SGAs conducted in this study. Vertical dotted lines represent the cut-offs used. (**c**) Plot showing distribution of the *gsk3* interaction values compared to the interaction values of the *gsk3 amk2* double mutant. Red lines represent the cut-offs used. (**d**) Plot showing distribution the *amk2* interaction values compared to the interaction values of the *gsk3 amk2* double mutant. Red lines represent the cut-offs used. (**e**) Venn diagram showing overlaps of negative genetic interactions for the *gsk3* and *amk2* single mutants and the *gsk3 amk2* double mutant. (**f**) Venn diagram showing overlaps of positive genetic interactions for the *gsk3* and *amk2* single mutants and the *gsk3 amk2* double mutant.

Given that *gsk3* and *amk2* mutants suppress TOR-related phenotypes additively (Fig. 2), we also conducted an SGA screen to interrogate triple mutant cells, with *gsk3Δ amk2Δ* double mutant cells as query^38^,^39^. As in the case of the single mutant query SGA screens, linkage effects for positive and negative interactions were present in the double mutant query (Fig. 5a, blue and red lines for negative and positive interactions, respectively). The distribution of interaction values of the double mutant query showed a larger dynamic range than the values of the single mutant queries. This result reflects that a larger number of interactions were identified with the *gsk3Δ amk2Δ* (Fig. 5b). The interactions of the *gsk3Δ amk2Δ* double mutant were positively correlated with those of both single mutant queries: *gsk3Δ* (R = 0.38, Fig. 5c) and *amk2Δ* (R = 0.46, Fig. 5d). A bias towards negative interactions was evident (see data points’ displacement in Figure c and d, and tail of double mutant line in Fig. 5b towards left part of graph). While yeast genomes only contain a few hundred essential genes, there are hundreds of thousands of double mutants that are lethal. For example, in budding yeast there are 1100 essential genes and 550,000 gene pairs that are essential together^40^. By extension, it is not surprising that triple mutants result in an even larger number of cases of synthetic lethality or sickness.

We identified 425 positive and 777 negative genetic interactions with *gsk3 amk2* double mutants (applying cut-offs of +0.15 and −0.15, respectively). While positive interactions showed no functional enrichment (Supplemental Table 2), negative interactions were enriched in the GO terms ‘chromatin and gene silencing’, ‘protein translation’ and ‘gene products for localisation to the mitochondria’. Gsk3 and AMPK might together regulate factors linked to chromatin function and to respiration and detoxification of metabolic waste products. Interestingly, negative interactions were also enriched for genes induced during the oxidative stress response^41^ Compared to the single mutant queries, 305 positive and 496 negative genetic interactions were specific for the double mutant query. While the negative interactions were enriched for highly expressed genes, mutants with sensitivity to 5′-fluorouracil, a uracil analogue commonly utilised as a chemotherapeutic agent in the treatment of a range of cancers^42^ are significantly under-represented. Notably, AMPK is emerging as an interesting metabolic tumour suppressor and a promising target for cancer prevention and therapy^43^. This result highlights the principle that targeting multiple proteins simultaneously, e.g. by combined drugs, can greatly increase the effectiveness to combat cancer cells^44^, and triple action treatments open up even more possibilities to kill cancer cells.

In mammalian cells, Gsk3 is primarily known for its role in canonical Wnt signalling via β-catenin^45^. Previous work has also shown that Gsk3 operates in parallel to AMPK for mTORC1 inhibition via TSC2^25^. Functional relationships of AMKP, Gsk3 and TOR are likely conserved from fission yeast to mammals. Our work provides rich data sets for both common and specific interactors that may relate to TORC1 function and associated cell physiology and disease.

## Additional Information

**How to cite this article**: Rallis, C. *et al*. Genetic interactions and functional analyses of the fission yeast *gsk3* and *amk2* single and double mutants defective in TORC1-dependent processes. *Sci. Rep.* **7**, 44257; doi: 10.1038/srep44257 (2017).

**Publisher's note:** Springer Nature remains neutral with regard to jurisdictional claims in published maps and institutional affiliations.

## Supplementary Material

Supplemental Tables
