# Corrigendum: Genetic interactions and functional analyses of the fission yeast *gsk3* and *amk2* single and double mutants defective in TORC1-dependent processes

**DOI:** 10.1038/srep46857

**Published:** 2017-07-10

**Authors:** Charalampos Rallis, StJohn Townsend, Jürg Bähler

Scientific Reports 7: Article number: 44257; 10.1038/srep44257 published online: 03 10 2017; updated: 07 10 2017

This article contains a typographical error in the Materials and Methods section under the subheading ‘Growth assays’.

‘Caffeine and rapamycin were used at concentrations of 100 mM and 100 ng mL^−1^, respectively while hydrogen peroxide at a concentration of 0.5 mM’.

should read:

‘Caffeine and rapamycin were used at concentrations of 10 mM and 100 ng mL^−1^, respectively while hydrogen peroxide at a concentration of 0.5 mM’.

