# Supplementary material for: Genetic interactions and functional analyses of the fission yeast gsk3 and amk2 single and double mutants defective in TORC1-dependent processes
Source: Sci Rep. 2017 Mar 10;7:44257. doi: 10.1038/srep44257 (PMC5345095; doi:10.1038/srep44257)
Supplement: Supplemental Tables [file srep44257-s1.pdf]

Genetic interactions and functional analyses of the fission yeast *gsk3* and *amk2* single and double mutants defective in TORC1-dependent processes

Charalampos Rallis, StJohn Townsend and Jürg Bähler

SUPPLEMENTAL TABLE 1

GENETIC INTERACTIONS RECORDED FROM ALL SGA EXPERIMENTS

Systematic Gene Name	Gsk3 Interaction	Amk2 Interaction	Interaction between Double Mutant Query and Library Mutant
SPAC1002.01	0.014	0.096	0.075
SPAC1002.02	0.026	-0.007	0.052
SPAC1002.03c	0.07	0.015	0.116
SPAC1002.05c	0.014	0.032	0.099
SPAC1002.06c	-0.001	0.01	0.027
SPAC1002.07c	0.026	-0.03	-0.228
SPAC1002.12c	0.036	-0.018	0.142
SPAC1002.14	-0.08	-0.002	0.031
SPAC1002.17c	0.037	-0.008	-0.072
SPAC1002.18	0.057	-0.044	-0.222
SPAC1002.19	0.134	0.053	-0.128
SPAC1002.20	0.033	0.015	-0.031
SPAC1006.01	0.021	-0.057	0.067
SPAC1006.03c	NA	NA	NA
SPAC1006.04c	-0.03	0.05	-0.227
SPAC1006.06	0.062	-0.063	-0.061
SPAC1039.01	-0.05	-0.04	0.013
SPAC1039.02	0.099	-0.009	0.256
SPAC1039.03	0.079	0.093	0.22
SPAC1039.04	0.055	0.01	0.061
SPAC1039.05c	-0.015	-0.016	0.107
SPAC1039.06	0.027	0.07	0.16
SPAC1039.08	0.071	-0.142	-0.024
SPAC1039.09	0.054	0.029	0.217
SPAC1039.10	0.036	-0.039	-0.515
SPAC105.01c	0.009	-0.034	0.113
SPAC105.02c	0.018	-0.028	0.049
SPAC105.03c	-0.057	-0.045	0.085
SPAC1071.02	0.264	0.162	-0.08
SPAC1071.03c	-0.015	0.066	0.144
SPAC1071.05	0.03	-0.067	-0.135
SPAC1071.06	0.002	0.104	0.091
SPAC1071.07c	-0.061	-0.059	-0.35
SPAC1071.08	-0.043	0.065	-0.093
SPAC1071.09c	-0.226	0.034	-0.484
SPAC1071.11	0.042	0.029	0.133
SPAC1071.12c	0.02	-0.009	-0.333
SPAC1093.03	-0.035	0.045	-0.05
SPAC1093.06c	0.059	0.029	0.121
SPAC10F6.04	0.001	0	0.231
SPAC10F6.05c	0.023	-1.145	-0.858
SPAC10F6.06	-0.283	-0.126	0.161
SPAC10F6.07c	0.017	0.036	0.184
SPAC10F6.08c	-0.063	-0.024	-0.191
SPAC10F6.11c	0.018	0.031	0.093
SPAC10F6.12c	-0.047	-0.024	0.05
SPAC10F6.14c	-0.023	-0.028	-0.159
SPAC10F6.15	-0.105	-0.017	0.061
SPAC10F6.17c	-0.017	0.056	-0.251
SPAC110.02	-0.199	-0.244	-0.607
SPAC1142.01	0.027	0.009	0.126
SPAC1142.02c	-0.018	0.049	-0.035
SPAC1142.03c	0.037	0.154	-0.009
SPAC1142.06	0.041	0.036	0.147
SPAC1142.08	-0.014	0.015	0.002
SPAC11D3.01c	-0.149	-0.125	-0.72
SPAC11D3.02c	-0.028	-0.049	-0.25
SPAC11D3.03c	-0.007	-0.024	0.158
SPAC11D3.04c	-0.064	0.006	0.062
SPAC11D3.05	-0.014	-0.022	-0.209
SPAC11D3.06	0.018	0.043	0.021
SPAC11D3.07c	-0.049	0.05	0.17

SPAC11D3.09	-0.06	-0.03	-0.075
SPAC11D3.10	-0.002	0.067	-0.028
SPAC11D3.13	0.032	0.093	-0.1
SPAC11D3.14c	-0.028	0.03	0.03
SPAC11D3.15	-0.004	0.059	-0.026
SPAC11D3.16c	-0.084	-0.083	-0.1
SPAC11D3.17	0.005	0.025	0.082
SPAC11E3.01c	0.069	-0.043	0.076
SPAC11E3.03	-0.095	-0.24	-0.242
SPAC11E3.04c	0.053	0.11	-0.026
SPAC11E3.05	-0.456	-0.916	-0.923
SPAC11E3.08c	0.059	0.289	0.343
SPAC11E3.09	0.101	-0.037	0.213
SPAC11E3.10	0.036	-0.017	-0.019
SPAC11E3.11c	0.019	0.114	0.153
SPAC11E3.12	-0.016	-0.004	-0.016
SPAC11E3.13c	-0.087	0.003	0.017
SPAC11E3.14	0.049	-0.045	0.066
SPAC11G7.01	-0.09	0.035	0.134
SPAC11G7.03	NA	NA	NA
SPAC11G7.06c	0	0.022	0.099
SPAC11H11.01	-0.233	0.072	-0.349
SPAC11H11.02c	-0.023	-0.092	-0.063
SPAC11H11.03c	-0.002	0.021	0.154
SPAC11H11.04	-0.139	0.059	-0.009
SPAC11H11.05c	0.053	0.05	0.306
SPAC1250.02	-0.043	-0.305	-0.537
SPAC1250.03	0.071	0.15	0.183
SPAC1250.05	-0.164	-0.039	-0.351
SPAC1296.01c	-0.127	-0.037	-0.69
SPAC1296.02	-0.364	-0.015	-0.657
SPAC1296.03c	-0.043	0.079	-0.603
SPAC1296.04	-0.273	-0.054	-0.35
SPAC1296.05c	-0.085	0.067	-0.153
SPAC12B10.01c	0.055	-0.042	-0.156
SPAC12B10.02c	0.121	NA	NA
SPAC12B10.03	-0.157	-0.228	-0.349
SPAC12B10.04	-0.066	-0.009	-0.094
SPAC12B10.05	-0.078	0.126	-0.479
SPAC12B10.07	0.06	-0.024	-0.535
SPAC12B10.09	-0.058	0.142	0.337
SPAC12B10.10	-0.085	0.045	0.087
SPAC12B10.11	0.053	0.014	-0.17
SPAC12B10.12c	0.292	-0.312	0.274
SPAC12B10.13	-0.069	0.078	-0.167
SPAC12B10.14c	-0.038	-0.056	-0.146
SPAC12B10.15c	0.022	0.043	0.1
SPAC12B10.16c	0.028	0.043	0.047
SPAC12G12.01c	0.023	0.036	-0.238
SPAC12G12.03	-0.101	-0.2	-0.064
SPAC12G12.07c	-0.009	0.019	-0.045
SPAC12G12.09	-0.095	-0.016	0.095
SPAC12G12.10	-0.038	0.076	-0.023
SPAC12G12.12	0.006	0.009	0.035
SPAC12G12.15	0.033	-0.051	-0.272
SPAC12G12.16c	-0.001	0.057	0.116
SPAC1327.01c	-0.471	-1.562	-0.925
SPAC1399.01c	0.012	-0.012	0.115
SPAC1399.02	-0.014	0.006	0.043
SPAC1399.03	-0.076	-0.008	-0.452
SPAC1399.04c	-0.026	-0.021	0.121
SPAC1399.05c	0.059	-0.021	-0.008
SPAC13A11.01c	-0.042	0.183	-0.072
SPAC13A11.03	-0.325	-0.022	-0.462
SPAC13A11.04c	-0.002	-0.044	-0.464
SPAC13A11.05	0.006	-0.41	-0.187
SPAC13A11.06	-0.074	0.06	-0.109
SPAC13C5.02	NA	NA	NA
SPAC13C5.03	-1.712	-0.471	-1.928
SPAC13C5.04	-0.033	-0.01	-0.097
SPAC13C5.05c	0.025	-0.191	0.037
SPAC13C5.06c	-0.024	-0.068	-0.107
SPAC13D6.01	0.02	-0.014	0.075

SPAC13D6.02c	-0.055	0.093	0.052
SPAC13D6.03c	0.1	0.175	0.3
SPAC13D6.04c	0.047	0.091	0.23
SPAC13F5.01c	-0.001	0.063	0.07
SPAC13F5.03c	-0.018	-0.047	-0.255
SPAC13F5.04c	0.005	0.009	0.122
SPAC13F5.05	-0.038	-0.094	0.162
SPAC13G6.01c	0.038	0.02	-0.24
SPAC13G6.02c	-0.197	-0.01	-0.633
SPAC13G6.03	-0.052	-0.184	-0.43
SPAC13G6.04	-0.075	-0.079	0.022
SPAC13G6.08	-0.133	-0.107	-0.283
SPAC13G6.10c	0.183	0.521	-0.084
SPAC13G6.12c	0.06	0.038	-0.189
SPAC13G6.13	0.047	0.092	0.145
SPAC13G6.15c	0.02	0.056	0.161
SPAC13G7.03	0.344	0.228	0.353
SPAC13G7.04c	-0.011	0.063	-0.36
SPAC13G7.05	0.013	0.043	0.067
SPAC13G7.06	0.026	-0.064	-0.344
SPAC13G7.07	0.058	-0.101	-0.283
SPAC13G7.09c	-0.051	0.036	0.111
SPAC13G7.11	-0.795	-0.319	-0.346
SPAC13G7.12c	0.272	0.272	0.211
SPAC13G7.13c	0.055	-0.069	0.06
SPAC140.01	0.019	-0.039	-0.18
SPAC140.03	-0.19	-0.286	-0.054
SPAC140.04	-0.015	-0.046	0.022
SPAC1420.01c	0.014	0.019	-0.426
SPAC1420.03	0.001	0	-1.049
SPAC144.01	0.029	0.036	0.135
SPAC144.02	0.459	-0.061	-0.218
SPAC144.03	0.015	-0.176	0.084
SPAC144.04c	-0.176	-0.112	-0.431
SPAC144.05	-0.029	0	0.231
SPAC144.06	0.179	-0.081	-0.651
SPAC144.11	-0.007	-0.168	-0.07
SPAC144.14	0.059	0.07	0.045
SPAC144.17c	0.019	0.054	0.09
SPAC1486.02c	0.01	-0.1	-0.57
SPAC1486.04c	-0.205	0.28	-0.202
SPAC14C4.01c	0.029	-0.064	-0.083
SPAC14C4.03	0.022	0.14	0.197
SPAC14C4.04	0.019	-0.033	0.148
SPAC14C4.05c	-0.024	-0.053	-0.358
SPAC14C4.06c	-0.013	0.029	-0.372
SPAC14C4.07	-0.008	0.057	-0.004
SPAC14C4.08	0.05	0.019	0.134
SPAC14C4.09	0.019	0.059	0.116
SPAC14C4.10c	-0.004	-0.015	0.136
SPAC14C4.11	0.027	-0.046	0.11
SPAC14C4.12c	0.041	-0.031	-0.028
SPAC14C4.13	-0.063	-0.122	-0.137
SPAC14C4.15c	0.061	-0.011	0.142
SPAC14C4.16	0.221	-1.341	-0.134
SPAC1527.01	-0.02	0.027	0.053
SPAC1527.03	-0.002	0.079	0.035
SPAC1556.01c	-0.14	-0.323	-0.707
SPAC1556.02c	-0.06	-0.415	0.069
SPAC1556.03	0	-0.037	0.049
SPAC1556.04c	-0.004	0.064	-0.049
SPAC1556.05c	-0.427	-0.437	-0.596
SPAC1565.01	-0.139	-1.094	-0.639
SPAC1565.02c	-0.08	-0.07	0.023
SPAC1565.03	-0.046	-0.046	0.094
SPAC1565.07c	-0.14	-0.082	-0.708
SPAC15A10.03c	0.042	0.003	0.107
SPAC15A10.05c	0.017	0.008	-0.234
SPAC15A10.07	-0.021	-0.012	-0.08
SPAC15A10.08	-0.045	0.064	-0.077
SPAC15A10.09c	0.019	0.034	-0.076
SPAC15A10.10	0.034	0.057	0.126
SPAC15A10.11	0.06	0.034	0.045

SPAC15A10.15	0.017	-0.002	0.169
SPAC15A10.16	0.004	0.082	0.069
SPAC15E1.02c	0.029	0.047	-0.213
SPAC15E1.05c	-0.003	0.03	0.069
SPAC15E1.06	-0.017	-0.224	-1.468
SPAC15E1.07c	-0.002	0.029	-0.285
SPAC15E1.09	0.107	-0.023	0.136
SPAC15F9.01c	-0.02	0.03	0.113
SPAC15F9.02	-0.035	-0.092	-0.004
SPAC16.01	-0.112	-0.073	-0.207
SPAC16.03c	-0.109	-0.078	-0.897
SPAC16.04	-0.001	-0.081	0.116
SPAC16.05c	-0.222	0.026	-1.008
SPAC1610.01	0	-0.276	-0.764
SPAC1610.03c	0.098	-0.036	0.197
SPAC1610.04	-0.02	0.057	0.046
SPAC1635.01	0.153	0.236	0.285
SPAC1639.02c	0.072	0.157	0.237
SPAC167.01	-0.144	-1.23	-0.944
SPAC167.04	0.023	0.01	-0.336
SPAC167.06c	-0.02	-0.015	-0.172
SPAC167.07c	-0.098	-0.037	-0.597
SPAC16A10.02	-0.035	0.048	-0.106
SPAC16A10.04	0.041	-0.017	-0.212
SPAC16A10.05c	0.159	0.19	-0.411
SPAC16E8.01	-0.173	-0.751	-0.685
SPAC16E8.05c	0.064	0.005	-0.063
SPAC16E8.06c	-0.047	-0.029	-0.036
SPAC16E8.08	0.017	-0.046	-0.08
SPAC16E8.12c	-0.03	0.01	0.026
SPAC16E8.13	-0.068	0.019	0.008
SPAC16E8.14c	-0.03	-0.015	-2
SPAC16E8.17c	0.036	0.088	0.142
SPAC16E8.18	-0.198	0.022	0.133
SPAC1705.02	0.025	0.028	0.093
SPAC1751.01c	0.006	0.025	-0.155
SPAC1751.04	0.073	-0.165	-0.025
SPAC1782.01	0.053	0.096	0.14
SPAC1782.02c	-0.003	0.064	0.069
SPAC1782.04	0	0.078	0.16
SPAC1782.06c	-0.002	-0.031	0.168
SPAC1782.08c	0.111	0.17	0.037
SPAC1782.09c	-0.166	-0.011	0.017
SPAC1782.11	0.594	0.091	NA
SPAC1783.01	-0.028	0.065	0.111
SPAC1783.02c	0.201	-0.28	-0.141
SPAC1783.04c	-0.122	-0.243	-0.238
SPAC1783.05	-0.006	0.03	-0.193
SPAC1783.06c	0.016	0.031	0.011
SPAC1783.08c	-0.109	0.067	-0.503
SPAC1786.01c	0.086	-0.02	0.034
SPAC1786.02	0.001	0.004	-0.352
SPAC1786.04	-0.071	-0.221	-0.114
SPAC17A2.01	-0.018	-0.025	0.062
SPAC17A2.02c	0.045	-0.046	-0.051
SPAC17A2.05	0.005	0.044	0.051
SPAC17A2.06c	-0.529	-1.187	-0.573
SPAC17A2.07c	-0.021	-0.1	0.071
SPAC17A2.10c	0.028	0.147	0.007
SPAC17A2.11	0.051	0.069	0.147
SPAC17A2.12	0.082	0.1	-0.475
SPAC17A2.13c	0.026	-0.035	-0.112
SPAC17A2.14	0.107	-0.095	0.313
SPAC17A5.01	-0.043	-0.021	-0.756
SPAC17A5.05c	-0.063	0.016	0.031
SPAC17A5.07c	-0.693	-0.199	-0.707
SPAC17A5.08	-0.026	-0.825	-0.696
SPAC17A5.09c	-0.19	-0.079	0.213
SPAC17A5.10	0.007	-0.001	-0.098
SPAC17A5.11	-0.015	0.037	0.146
SPAC17A5.16	0.105	-0.638	-0.316
SPAC17A5.18c	-0.015	0.001	0.005
SPAC17C9.05c	-0.002	-0.196	-0.02

SPAC17C9.08	-0.039	0.132	0.081
SPAC17C9.09c	-0.024	0.009	0.175
SPAC17C9.10	-0.015	-0.033	0.066
SPAC17C9.11c	0.022	0.025	0.141
SPAC17C9.14	-0.097	-0.147	-0.754
SPAC17C9.15c	0.16	0.07	0.345
SPAC17C9.16c	-0.001	-0.099	0.011
SPAC17D4.01	0.002	-0.023	-0.169
SPAC17D4.03c	0.02	-0.147	0.051
SPAC17D4.04	0.057	0.051	0.058
SPAC17G6.02c	0.024	-0.023	0.17
SPAC17G6.03	-0.064	-0.024	0.081
SPAC17G6.04c	0.218	0.235	0.184
SPAC17G6.05c	-0.042	0.183	0.269
SPAC17G6.06	-0.032	0.013	0.023
SPAC17G6.08	0.018	-0.138	-0.143
SPAC17G6.13	0.105	-0.063	-0.045
SPAC17G6.15c	0.025	-0.115	-0.15
SPAC17G6.17	0.005	0.001	-0.595
SPAC17G8.07	0.113	0.08	0.145
SPAC17G8.08c	0.048	-0.038	0.028
SPAC17G8.09	-0.06	-0.005	0.247
SPAC17G8.10c	-0.083	-0.06	-0.046
SPAC17G8.11c	0.049	0.027	-0.83
SPAC17G8.14c	0.09	-0.007	-0.061
SPAC17H9.01	0.024	0.003	0.072
SPAC17H9.03c	0.051	0.058	0.131
SPAC17H9.04c	0.077	0.08	0.068
SPAC17H9.06c	-0.004	0.009	0.098
SPAC17H9.11	0.034	0.076	-0.148
SPAC17H9.12c	-0.055	-0.053	-0.03
SPAC17H9.13c	-0.229	-0.751	0.069
SPAC17H9.14c	0.077	0.01	0.246
SPAC1805.01c	0.035	0.064	-0.613
SPAC1805.02c	-0.025	-0.116	0.06
SPAC1805.03c	0.017	-0.018	0.104
SPAC1805.04	-0.008	0.101	0.057
SPAC1805.06c	-0.032	-0.031	0.074
SPAC1805.07c	0.257	0.404	0.465
SPAC1805.08	0.049	0.026	0.106
SPAC1805.09c	-0.025	0.041	-0.106
SPAC1805.10	0.071	-0.056	0.171
SPAC1805.11c	0.078	0.209	0.145
SPAC1805.12c	-0.074	-0.039	0.17
SPAC1805.14	0.012	0.004	-0.026
SPAC1805.15c	0.061	0.041	0.18
SPAC1805.16c	0.019	-2	-0.474
SPAC1834.03c	0.013	-0.032	0.033
SPAC1834.04	-0.006	0.032	-0.047
SPAC1834.05	0.294	0.128	0.201
SPAC1834.07	0.039	0.03	0.053
SPAC1834.08	0.056	0.088	0.213
SPAC1834.09	-0.034	-0.038	-0.156
SPAC1834.10c	-0.011	-0.044	0.189
SPAC1851.02	0.18	-0.434	-1.105
SPAC1851.04c	NA	NA	NA
SPAC186.01	0.006	0.032	0.111
SPAC186.02c	0.013	0.046	0.083
SPAC186.03	-0.041	0.053	-0.151
SPAC186.05c	0.008	-0.022	0.105
SPAC186.06	0.052	0.024	0.189
SPAC186.07c	-0.022	0.015	0.027
SPAC186.08c	-0.043	-0.126	-0.49
SPAC186.09	-0.017	-0.031	0.009
SPAC18B11.02c	-0.03	-0.06	0.056
SPAC18B11.03c	0.066	0.027	0.08
SPAC18B11.04	-0.061	0.039	0.019
SPAC18B11.07c	0.132	0.057	0.187
SPAC18B11.08c	0.033	0.01	-0.125
SPAC18B11.09c	-0.008	0.014	-0.003
SPAC18B11.11	0.039	-0.162	0.048
SPAC18G6.01c	0.076	-0.001	0.096
SPAC18G6.02c	-0.065	-0.199	-0.837

SPAC18G6.04c	0.023	-0.031	-0.458
SPAC18G6.05c	-0.079	-0.01	-0.243
SPAC18G6.10	-0.243	-0.407	-1.082
SPAC18G6.12c	0.01	0.028	0.08
SPAC18G6.13	0.08	0.003	0.129
SPAC18G6.15	0.021	0.034	-0.194
SPAC1952.02	-0.076	-0.069	-0.052
SPAC1952.03	-0.052	-0.2	-0.083
SPAC1952.05	-0.026	-0.01	0.158
SPAC1952.06c	0.031	0.059	0.239
SPAC1952.07	0.069	0.074	0.261
SPAC1952.08c	0.026	0.064	0.128
SPAC1952.09c	0.055	0.056	-0.126
SPAC1952.10c	0.004	-0.012	0.137
SPAC1952.11c	-0.02	-0.008	-0.111
SPAC1952.12c	0.011	0.021	0.199
SPAC1952.15c	-0.034	0.065	0.05
SPAC1952.16	0.199	0.133	0.185
SPAC1952.17c	-0.049	-0.057	0.168
SPAC19A8.01c	-0.015	-0.052	-0.249
SPAC19A8.02	-0.028	-0.025	0.24
SPAC19A8.03	0.66	0.757	NA
SPAC19A8.04	-0.102	-0.001	-0.175
SPAC19A8.05c	-0.528	-0.147	-0.425
SPAC19A8.08	0.044	-0.07	-0.3
SPAC19A8.10	0.082	-0.276	0.189
SPAC19A8.14	0.017	0.042	0.129
SPAC19B12.04	-0.052	-0.036	-0.172
SPAC19B12.06c	-0.012	-0.182	0.123
SPAC19B12.07c	0.051	0.089	0.164
SPAC19B12.08	0.009	-0.007	0.076
SPAC19B12.09	0.037	0.06	0.038
SPAC19B12.10	-0.565	-1.027	-0.517
SPAC19B12.11c	-0.011	0.1	0.068
SPAC19B12.12c	-0.035	-0.037	0.046
SPAC19D5.01	0.071	0.043	-0.163
SPAC19D5.02c	0.011	-0.024	0.177
SPAC19D5.03	-0.056	0.063	0.034
SPAC19D5.06c	0.016	0.042	0.314
SPAC19D5.07	-0.004	0.006	0.064
SPAC19D5.11c	-0.112	0.019	0.105
SPAC19E9.01c	-0.015	-0.047	-0.178
SPAC19E9.02	0.035	0.051	0.183
SPAC19E9.03	0.091	-0.051	-0.26
SPAC19G12.02c	-0.026	-0.063	-0.009
SPAC19G12.03	0.023	0.023	-0.13
SPAC19G12.04	0.007	-0.004	0.08
SPAC19G12.05	-0.028	-0.298	-0.702
SPAC19G12.08	-0.192	-0.333	-1.003
SPAC19G12.09	0.009	0.026	0.032
SPAC19G12.12	0.016	-0.005	-0.033
SPAC19G12.13c	0.199	0.027	0.023
SPAC19G12.15c	-0.061	-0.765	-1.193
SPAC19G12.16c	0.011	0.048	-0.166
SPAC1B1.02c	-0.229	0.056	-0.418
SPAC1B1.04c	0.075	0.028	0.053
SPAC1B3.01c	0.011	-0.026	0.094
SPAC1B3.02c	-0.043	0.085	0.048
SPAC1B3.03c	-0.23	-0.049	-0.134
SPAC1B3.04c	0.003	0.065	-0.023
SPAC1B3.05	0	0.119	-0.212
SPAC1B3.06c	0.021	-0.011	0.049
SPAC1B3.07c	0.016	0.065	0.133
SPAC1B3.08	0.025	0.11	0.016
SPAC1B3.10c	0.012	0.009	0.056
SPAC1B3.15c	-0.029	0.041	0.094
SPAC1B3.16c	-0.192	-0.141	-0.472
SPAC1B3.17	0.047	-0.06	-1.515
SPAC1B9.02c	-0.082	-0.086	-0.995
SPAC1D4.01	-0.049	0.012	-0.302
SPAC1D4.02c	-0.05	-0.048	-0.304
SPAC1D4.03c	-0.136	-0.005	-1.204
SPAC1D4.05c	-0.136	0.007	-0.074

SPAC1D4.06c	-0.02	0.074	-1.506
SPAC1D4.09c	-0.161	0.005	-0.005
SPAC1D4.11c	0.114	-0.041	-0.206
SPAC1F12.04c	0.043	0.005	0.181
SPAC1F12.05	0.003	0.04	0.018
SPAC1F12.06c	-0.422	-0.882	-0.448
SPAC1F12.10c	0.04	0.105	-0.301
SPAC1F3.05	-0.106	0.036	-0.208
SPAC1F3.06c	-0.093	0.035	0.077
SPAC1F3.09	-0.343	-1.389	-1.225
SPAC1F5.03c	0.039	0.031	0.088
SPAC1F5.05c	-0.227	-0.299	-0.797
SPAC1F5.07c	-0.038	-0.037	-0.183
SPAC1F5.08c	0.159	0.363	0.466
SPAC1F5.09c	-0.093	-0.033	-0.013
SPAC1F7.06	0.009	0.052	0.05
SPAC1F7.08	0.602	0.917	0.754
SPAC1F7.09c	0.084	0.014	-0.516
SPAC1F7.10	0.067	0.03	0.096
SPAC1F7.11c	0.03	0.114	0.172
SPAC1F7.12	0.05	0.051	0.134
SPAC1F8.01	0.001	0.101	0.196
SPAC1F8.02c	0.011	0.108	0.13
SPAC1F8.03c	0.002	-0.043	-0.036
SPAC1F8.05	-0.076	-0.133	-0.022
SPAC1F8.06	-0.101	-0.07	-0.226
SPAC1F8.08	0.001	0.029	0.183
SPAC20G4.01	0.018	0.065	-0.115
SPAC20G4.02c	0.051	0.014	0.071
SPAC20G4.03c	-0.003	-0.05	0.168
SPAC20G4.04c	0.013	-0.009	-1.175
SPAC20G4.05c	-0.008	0	-0.559
SPAC20G4.08	-0.041	-0.024	-0.075
SPAC20G8.02	0.004	0.061	-0.074
SPAC20G8.04c	-0.09	-0.068	0.112
SPAC20G8.07c	0.01	-0.009	0.142
SPAC20G8.08c	-0.052	-0.005	-0.028
SPAC20G8.09c	-0.016	-0.131	0.027
SPAC20G8.10c	0.631	0.41	0.294
SPAC20H4.03c	-0.094	-0.037	-1.092
SPAC20H4.04	-0.08	-0.02	-0.077
SPAC20H4.05c	-0.106	-0.158	-0.052
SPAC20H4.06c	0.058	0.075	0.161
SPAC20H4.07	-0.062	-0.04	0.046
SPAC20H4.09	-0.077	0.026	0.081
SPAC20H4.10	-0.051	0.114	0.123
SPAC20H4.11c	0.06	-0.017	-0.111
SPAC212.01c	-0.028	-0.052	0.09
SPAC212.02	-0.01	0.009	0.141
SPAC212.03	0.023	0.01	0.088
SPAC212.04c	0.462	0.494	0.46
SPAC212.08c	-0.069	-0.056	-0.046
SPAC21E11.03c	0.041	-0.013	0.27
SPAC21E11.04	0.029	0.069	-0.192
SPAC21E11.05c	-0.01	0.091	0.037
SPAC227.01c	-0.248	0.12	-0.608
SPAC227.03c	-0.033	0.069	-0.032
SPAC227.04	0.013	-0.017	0.202
SPAC227.05	0.106	-0.123	-0.469
SPAC227.06	-0.084	0.011	0.125
SPAC227.07c	-0.02	-0.042	-0.08
SPAC227.10	0.04	0.042	-0.378
SPAC227.11c	-0.032	0.004	-0.086
SPAC227.15	-0.481	0.151	-0.885
SPAC227.18	0.005	-0.951	0.043
SPAC22A12.01c	-0.107	0.033	-0.691
SPAC22A12.02c	0.146	0.028	0.104
SPAC22A12.03c	-0.194	-0.063	-0.349
SPAC22A12.04c	-0.028	0.046	-0.347
SPAC22A12.06c	-0.073	-0.046	0.059
SPAC22A12.10	-0.003	-1.808	-0.165
SPAC22A12.11	-0.113	-0.06	-0.094
SPAC22A12.14c	0.002	0.059	0.121

SPAC22A12.16	-0.019	-0.107	0.002
SPAC22A12.17c	-0.012	-0.041	0.088
SPAC22E12.01	-0.049	0.089	0.011
SPAC22E12.03c	-0.092	0.084	-0.278
SPAC22E12.04	0.229	0.405	0.276
SPAC22E12.05c	-0.027	0.013	0.122
SPAC22E12.06c	0.021	0.018	-0.103
SPAC22E12.11c	-0.038	-0.744	-0.158
SPAC22E12.14c	0.084	-0.002	0.296
SPAC22E12.19	0.009	0.008	-0.698
SPAC22F3.02	-0.097	-0.086	-0.736
SPAC22F3.03c	-0.011	0.041	0.139
SPAC22F3.04	-0.1	0.014	-0.028
SPAC22F3.06c	-0.899	-0.175	-1.373
SPAC22F3.07c	-0.227	-0.087	-1.056
SPAC22F3.08c	-0.402	-0.016	-1.071
SPAC22F3.09c	-0.17	0.303	0.157
SPAC22F3.11c	-0.047	-0.071	0
SPAC22F3.12c	-1.664	-2	-2
SPAC22F3.13	-0.053	-0.055	-0.472
SPAC22F8.02c	-0.047	0.062	-0.057
SPAC22F8.03c	-0.015	0.01	-0.002
SPAC22F8.04	-0.076	0.134	0.255
SPAC22F8.05	0.039	0.036	-0.105
SPAC22F8.07c	-0.019	0.036	-0.138
SPAC22F8.09	0.064	0.05	0.048
SPAC22F8.11	0.611	0.611	0.667
SPAC22G7.01c	-0.049	0.095	-0.096
SPAC22H10.02	0.031	-0.135	0.151
SPAC22H10.03c	0.011	0.071	0.124
SPAC22H10.04	0.126	-0.013	-1.268
SPAC22H10.08	0.03	-0.135	0.024
SPAC22H10.09	0.165	0.023	-1.335
SPAC22H10.11c	0.101	0.153	-0.417
SPAC22H10.13	0.074	0.105	0.133
SPAC23A1.02c	0.012	0.045	0.056
SPAC23A1.03	0.205	0.029	0.175
SPAC23A1.04c	0.066	0.018	0.231
SPAC23A1.06c	0.061	0.091	-0.066
SPAC23A1.07	0.048	0.018	-0.202
SPAC23A1.09	0.035	0.068	0.136
SPAC23A1.11	0.027	0.083	-0.212
SPAC23A1.14c	0.013	0.009	-0.054
SPAC23C11.01	0.044	-0.013	0.023
SPAC23C11.02c	-0.207	0.17	-1.129
SPAC23C11.04c	-0.153	-0.267	-0.863
SPAC23C11.06c	0.049	-0.079	0.094
SPAC23C11.07	0.067	0.049	0.214
SPAC23C11.08	0.136	0.107	-0.368
SPAC23C11.10	-0.159	-0.147	-1.101
SPAC23C11.13c	0.025	-0.039	0.059
SPAC23C11.14	0.054	0.024	0.118
SPAC23D3.01	0.04	-0.023	-0.039
SPAC23D3.03c	0.027	0.041	0.18
SPAC23D3.04c	0.004	-0.004	-0.209
SPAC23D3.09	-0.169	-0.138	-0.215
SPAC23D3.10c	-0.03	0.046	0.094
SPAC23D3.11	-0.01	0.011	-0.115
SPAC23D3.12	0.009	-0.071	-0.096
SPAC23D3.13c	-0.05	-0.028	-0.01
SPAC23E2.01	-0.175	0.123	-0.13
SPAC23H3.04	-0.037	0.128	0.226
SPAC23H3.05c	-0.083	-0.014	-0.499
SPAC23H3.06	0.188	-0.158	0.086
SPAC23H3.08c	-0.02	-0.049	0.208
SPAC23H3.11c	0.01	0	0.126
SPAC23H3.12c	-0.034	-0.012	-0.041
SPAC23H3.14	0.029	0.069	0.132
SPAC23H3.15c	0.101	0.125	0.099
SPAC23H4.01c	0.043	0.009	0.013
SPAC23H4.02	-0.068	-0.065	0.126
SPAC23H4.08	-0.012	0.022	-0.032
SPAC23H4.09	-0.049	-0.07	-0.039

SPAC23H4.10c	0.464	-0.152	-0.687
SPAC23H4.16c	0.035	-0.019	-0.336
SPAC23H4.17c	0.014	-0.048	-0.151
SPAC24B11.07c	-0.097	-0.018	0.15
SPAC24B11.08c	0.049	-0.02	-0.053
SPAC24B11.10c	0.062	-0.029	0.079
SPAC24B11.12c	0.237	-1.582	-0.96
SPAC24B11.13	-0.124	-0.363	-0.548
SPAC24C9.02c	0.031	0.147	0.33
SPAC24C9.05c	0.045	0.229	-0.873
SPAC24C9.07c	0.036	0.158	-0.264
SPAC24C9.08	0.043	-0.013	0.152
SPAC24C9.14	0.064	-0.029	0.204
SPAC24C9.15c	0.071	0.039	-0.282
SPAC24C9.16c	0.076	-0.061	0.221
SPAC24H6.02c	-0.046	0.063	-0.061
SPAC24H6.03	-0.091	0.003	0.019
SPAC24H6.04	-0.036	0.108	0.034
SPAC24H6.07	-0.218	0.134	-0.629
SPAC24H6.08	-0.131	-0.184	-0.087
SPAC24H6.09	0.256	0.289	0.104
SPAC24H6.10c	0.048	0.044	0.217
SPAC24H6.11c	0.253	0.167	0.219
SPAC24H6.13	-0.134	0.09	0.157
SPAC25A8.01c	-0.051	-0.102	-0.025
SPAC25A8.02	-0.014	-0.033	0.161
SPAC25A8.03c	-0.026	0.023	-0.156
SPAC25B8.01	0.018	-0.126	-0.004
SPAC25B8.05	0.136	-0.004	-0.519
SPAC25B8.07c	-0.051	0.045	-0.073
SPAC25B8.08	0.045	0.083	0.105
SPAC25B8.09	-0.012	-0.024	0.125
SPAC25B8.10	0.016	-0.058	0.124
SPAC25B8.11	0.057	0.014	0.248
SPAC25B8.13c	-0.117	-0.543	-1.512
SPAC25B8.15c	-0.013	-0.006	0.142
SPAC25B8.17	-0.157	-0.13	-0.524
SPAC25B8.19c	-0.067	-0.12	-0.66
SPAC25G10.01	0.038	0.018	0.024
SPAC25G10.02	0.112	0.047	0.264
SPAC25G10.05c	0.036	-0.215	-0.198
SPAC25G10.06	-0.071	0.208	-0.565
SPAC25G10.09c	-0.04	-0.093	-0.076
SPAC25H1.02	0.103	0.093	0.141
SPAC25H1.03	0.124	0.111	-0.04
SPAC25H1.04	-0.017	-0.032	0.098
SPAC25H1.07	0.419	-0.928	0.288
SPAC25H1.09	0.07	0.052	0.102
SPAC26A3.01	-0.098	-0.164	-0.402
SPAC26A3.02	-0.069	-0.018	0.021
SPAC26A3.04	-0.213	-0.168	-0.481
SPAC26A3.06	-0.117	0.016	0.193
SPAC26A3.07c	0.131	0.12	-0.435
SPAC26A3.09c	-0.202	0.042	0.08
SPAC26A3.10	-0.078	0.058	-0.042
SPAC26A3.11	-0.011	-0.018	-0.011
SPAC26A3.14c	-0.024	0.137	-0.106
SPAC26A3.16	0.026	0.142	0.158
SPAC26A3.17c	-0.004	-0.007	-0.031
SPAC26F1.01	-0.168	-0.005	-0.527
SPAC26F1.02	-0.09	0.011	0.202
SPAC26F1.05	0.017	0.007	-0.395
SPAC26F1.07	-0.006	-0.1	-0.326
SPAC26F1.08c	0.06	-0.031	0.201
SPAC26F1.09	0.126	0.11	-0.067
SPAC26F1.10c	0.035	0.011	-0.117
SPAC26F1.12c	-0.068	-0.006	-0.023
SPAC26F1.14c	0.079	-0.129	0.195
SPAC26H5.02c	0.1	0.017	0.073
SPAC26H5.03	-0.015	-0.872	-0.591
SPAC26H5.04	-0.049	0.062	-0.347
SPAC26H5.07c	0.069	-0.008	-0.09
SPAC26H5.08c	-0.156	-0.099	0.106

SPAC26H5.09c	0.019	-0.001	-0.059
SPAC26H5.10c	0.025	0.013	0.165
SPAC27D7.02c	0.012	-0.039	-0.138
SPAC27D7.03c	0.018	0.006	0.143
SPAC27D7.04	0.055	0.096	0.094
SPAC27D7.05c	0.009	0.04	0.045
SPAC27D7.06	-0.015	-0.022	0.103
SPAC27D7.08c	0.097	0.162	-0.585
SPAC27D7.11c	-0.043	0.001	0.177
SPAC27D7.12c	-0.039	0.023	0.061
SPAC27D7.13c	0.042	0.072	0.052
SPAC27E2.01	0.014	-1.396	-0.745
SPAC27E2.02	-0.025	-0.026	0.128
SPAC27E2.03c	0.057	0.169	-0.876
SPAC27E2.09	-0.044	0.013	-0.205
SPAC27F1.03c	0.041	0.132	0.102
SPAC27F1.05c	0.129	-0.038	0.116
SPAC27F1.06c	-0.041	0.058	-0.114
SPAC27F1.08	-0.367	-0.04	-0.969
SPAC27F1.10	0.072	0.012	0.198
SPAC29A4.02c	0.018	0.01	0.014
SPAC29A4.05	0.035	-0.12	0.058
SPAC29A4.09	0.094	0.07	-0.072
SPAC29A4.11	0.051	-0.128	-0.104
SPAC29A4.13	0.04	0.039	0.17
SPAC29A4.14c	-0.056	-0.073	-0.24
SPAC29A4.17c	-0.048	-0.145	0
SPAC29A4.19c	0.032	0.063	-0.041
SPAC29A4.20	0.07	0.091	-0.194
SPAC29B12.02c	-0.069	0.036	0.038
SPAC29B12.03	0.052	0.163	0.203
SPAC29B12.06c	-0.011	-0.052	-0.228
SPAC29B12.08	0.092	0.061	-0.171
SPAC29B12.10c	0.009	-0.047	-0.03
SPAC29B12.11c	0.009	-0.073	-0.142
SPAC29B12.12	0.058	0.087	-0.217
SPAC29B12.13	0.025	0.002	0.081
SPAC29B12.14c	-0.024	-0.015	0.123
SPAC29E6.01	0.002	0.028	-0.096
SPAC29E6.05c	-0.05	0.063	-0.111
SPAC29E6.07	0.074	-0.017	-0.012
SPAC29E6.09	-0.029	-0.026	-0.232
SPAC29E6.10c	-0.015	0.06	-0.229
SPAC2C4.05	0.254	-0.484	-0.278
SPAC2C4.06c	-0.042	-0.142	0.014
SPAC2C4.07c	-0.063	-0.054	0.086
SPAC2C4.08	0.012	-0.025	-0.315
SPAC2C4.09	0.076	-0.008	-0.276
SPAC2C4.10c	0.017	-0.543	-0.009
SPAC2C4.14c	0.077	-0.192	-0.142
SPAC2C4.15c	-0.001	0.004	0.083
SPAC2C4.17c	0.06	-0.017	-0.025
SPAC2E12.03c	-0.011	-0.033	0.141
SPAC2E1P3.02c	-0.031	0.134	0.218
SPAC2E1P3.04	0.003	0.117	-0.101
SPAC2E1P5.01c	-0.039	0.049	0.237
SPAC2E1P5.02c	0.019	0.076	-0.193
SPAC2E1P5.03	0.063	0.073	0.205
SPAC2F3.01	-0.057	0.085	0.142
SPAC2F3.02	0.086	0.102	-0.439
SPAC2F3.05c	0.049	0.017	0.155
SPAC2F3.08	0.191	0.515	0.32
SPAC2F3.12c	0.004	-0.079	-0.386
SPAC2F3.15	0.409	0.16	-0.006
SPAC2F3.16	0.065	0.049	0.215
SPAC2F3.18c	0.031	-0.019	-0.146
SPAC2F7.02c	-0.151	0.091	-0.153
SPAC2F7.03c	-0.152	-0.002	-0.235
SPAC2F7.04	-0.089	-0.007	-0.723
SPAC2F7.06c	-0.086	-0.153	0.013
SPAC2F7.08c	-0.022	-0.079	-0.051
SPAC2F7.09c	-0.015	-0.008	-0.182
SPAC2F7.10	0.026	-0.126	-0.996

SPAC2F7.11	0.115	-0.055	-0.703
SPAC2F7.17	-0.088	0.151	-0.301
SPAC2H10.01	0.019	0.018	0.04
SPAC2H10.02c	0.048	0.03	0.026
SPAC30.01c	-0.028	0.061	0.137
SPAC30.02c	0.611	NA	NA
SPAC30.03c	-0.065	0.047	0.128
SPAC30.04c	-0.008	-0.008	0.132
SPAC30C2.02	-0.276	-0.21	-1.261
SPAC30C2.04	-0.049	-0.14	-0.839
SPAC30C2.06c	-0.708	-0.04	0.525
SPAC30C2.07	-0.046	0.113	0.152
SPAC30C2.08	0.04	0.073	0.047
SPAC31A2.09c	-0.067	-0.137	-0.06
SPAC31A2.12	-0.174	0.114	0.215
SPAC31A2.14	-0.149	0.024	-0.014
SPAC31A2.15c	-0.111	-0.099	-0.497
SPAC31A2.16	-0.024	0.07	-0.148
SPAC31G5.04	0.115	0.056	-0.328
SPAC31G5.07	-0.012	0.056	0.198
SPAC31G5.10	0.002	-0.05	0.065
SPAC31G5.11	-0.045	0.098	-0.1
SPAC31G5.12c	-0.116	-0.052	-0.092
SPAC31G5.14	-0.021	-0.026	0.103
SPAC31G5.15	0.009	0.014	-0.158
SPAC31G5.17c	0.042	-0.231	-0.388
SPAC31G5.18c	-0.446	-1.076	-0.515
SPAC323.01c	0.248	0.45	0.006
SPAC323.03c	0.014	0.022	-0.098
SPAC323.04	-0.003	-0.044	-0.012
SPAC328.01c	-0.054	-0.138	-0.012
SPAC328.03	-0.44	-0.82	-0.528
SPAC328.04	0.109	-0.045	-0.072
SPAC328.05	-0.048	0.003	0.011
SPAC328.06	-0.566	0.093	0.294
SPAC328.07c	0.095	0.041	0.217
SPAC328.09	0.092	0.067	0.189
SPAC328.10c	-0.02	0.002	-0.327
SPAC32A11.01	0	0.024	-0.981
SPAC32A11.02c	-0.062	-0.054	-0.077
SPAC32A11.03c	-0.04	0.144	-0.039
SPAC343.04c	-0.058	-0.177	-0.3
SPAC343.06c	-0.028	-0.006	0.026
SPAC343.07	-0.025	0.076	0.057
SPAC343.09	0.073	-0.63	-0.676
SPAC343.10	-0.024	-0.018	-0.515
SPAC343.11c	0.014	0.041	0.033
SPAC343.12	-0.149	-0.043	-0.481
SPAC343.15	0.05	0.179	-1.096
SPAC343.16	-0.005	0.007	-0.414
SPAC343.18	0.008	-0.046	-0.018
SPAC343.19	-0.024	0.096	0.012
SPAC343.20	0.021	0.039	0.25
SPAC3A11.02	0.046	0.067	-0.092
SPAC3A11.03	0.01	-0.033	-0.169
SPAC3A11.04	0.047	-0.029	0.06
SPAC3A11.05c	0.101	0.063	0.034
SPAC3A11.06	-0.056	-0.065	-0.035
SPAC3A11.07	-0.018	-0.173	-0.193
SPAC3A11.08	-0.577	0.199	-0.941
SPAC3A11.10c	0.048	-0.099	0.288
SPAC3A11.11c	0.002	-0.008	-0.045
SPAC3A11.13	0.096	0.015	0.003
SPAC3A11.14c	-0.095	-0.018	0.164
SPAC3A12.03c	-0.05	0.102	0.181
SPAC3A12.06c	-0.079	0.02	0.172
SPAC3A12.08	0.054	-0.047	0.153
SPAC3A12.09c	0.014	-0.014	-0.145
SPAC3A12.10	0.094	-0.567	-0.161
SPAC3A12.12	-0.048	-0.044	-0.275
SPAC3A12.13c	-0.467	-0.583	-0.86
SPAC3A12.17c	-0.014	-0.028	0.092
SPAC3C7.01c	-0.001	-0.079	0.092

SPAC3C7.02c	0.058	-0.008	-0.034
SPAC3C7.03c	-0.108	-0.099	-0.48
SPAC3C7.04	-0.006	-0.161	-0.425
SPAC3C7.05c	-0.025	-0.037	0.216
SPAC3C7.06c	0.011	-0.103	-0.127
SPAC3C7.07c	-0.037	-0.112	0.018
SPAC3C7.08c	0.153	-0.006	-0.133
SPAC3C7.09	-0.003	-0.016	-0.001
SPAC3C7.10	-0.102	-0.029	-0.736
SPAC3C7.12	0.099	0.082	-0.864
SPAC3C7.13c	0.032	-0.036	0.024
SPAC3C7.14c	0.02	0.045	-0.097
SPAC3F10.02c	0.007	0.046	0.077
SPAC3F10.04	0.047	-0.179	-0.299
SPAC3F10.05c	0.041	0.004	0.15
SPAC3F10.06c	-0.01	0.061	-0.023
SPAC3F10.09	-0.016	-0.207	-0.858
SPAC3F10.10c	0	-0.335	0.217
SPAC3F10.11c	-1.373	-1.078	-1.067
SPAC3F10.12c	-0.062	-0.019	-0.132
SPAC3F10.13	-0.107	-0.079	-0.129
SPAC3F10.18c	-0.01	-0.027	0.138
SPAC3G6.02	0.009	-0.07	0.04
SPAC3G6.03c	0.012	-0.01	0.093
SPAC3G6.04	-0.064	0.063	-0.115
SPAC3G6.05	-0.011	-0.031	-0.067
SPAC3G6.06c	0.221	0.241	0.241
SPAC3G6.09c	0.085	-0.068	0.078
SPAC3G6.11	0.01	0.056	0.019
SPAC3G6.13c	-0.022	0.007	-0.126
SPAC3G9.01	-0.116	-0.009	0.001
SPAC3G9.03	-0.101	-0.012	-0.178
SPAC3G9.04	0.083	-0.329	-0.597
SPAC3G9.05	0.001	0	-0.194
SPAC3G9.07c	-0.166	-0.046	-0.43
SPAC3G9.11c	-0.049	0.011	-0.045
SPAC3H1.03	0.009	0.105	-0.021
SPAC3H1.05	-0.014	-0.134	0.101
SPAC3H1.06c	-0.145	-0.083	-0.083
SPAC3H1.07	0.023	0.068	0.242
SPAC3H1.08c	-0.16	-0.134	-0.216
SPAC3H1.09c	0.057	0.178	0.168
SPAC3H1.10	0.041	0.033	0.148
SPAC3H1.12c	0.047	0.079	-0.064
SPAC3H1.13	0.078	0.054	0.228
SPAC3H1.14	-0.043	-0.088	0.157
SPAC3H5.04	0.01	0.116	0.138
SPAC3H5.05c	-0.002	0.013	-0.133
SPAC3H5.07	-0.032	0.017	-0.122
SPAC3H5.08c	0.044	0.069	0.212
SPAC3H5.09c	-0.006	0.102	0.185
SPAC3H5.12c	-0.067	0.121	-0.306
SPAC3H8.02	-0.558	-1.473	-0.913
SPAC3H8.03	0.024	-0.043	-0.069
SPAC3H8.04	-0.066	0.03	-0.458
SPAC3H8.07c	-0.136	0.015	-0.324
SPAC3H8.08c	-0.187	-0.083	-0.436
SPAC3H8.09c	0.02	-0.058	-0.115
SPAC458.02c	-0.002	-0.013	0.088
SPAC458.04c	-0.021	-0.018	-0.169
SPAC458.05	-0.759	0.4	0.664
SPAC458.06	0.037	0.031	0.135
SPAC4A8.02c	0.047	0.051	0.132
SPAC4A8.04	-0.102	-0.007	-0.355
SPAC4A8.05c	-0.004	0.003	-0.298
SPAC4A8.06c	-0.024	-0.063	0.041
SPAC4A8.07c	-0.021	-0.014	-0.137
SPAC4A8.09c	-0.152	-0.083	-0.685
SPAC4A8.10	-0.125	-0.054	-0.249
SPAC4A8.14	0.046	0.127	0.124
SPAC4C5.01	0.048	0.129	0.047
SPAC4C5.02c	NA	0.097	NA
SPAC4C5.03	0.016	0.019	0.082

SPAC4C5.04	-0.035	0.197	0.543
SPAC4D7.01c	0.043	NA 0.056	0.061
SPAC4D7.02c	0.006	0.006	0.058
SPAC4D7.03	-0.185	-0.043	-1.069
SPAC4D7.06c	0.029	-0.703	-0.37
SPAC4D7.07c	0.012	0.018	0.096
SPAC4D7.11	-0.052	-0.042	-0.239
SPAC4F10.02	0.012	-0.026	-0.079
SPAC4F10.04	-0.15	0.244	0.529
SPAC4F10.07c	0.103	0.074	0.136
SPAC4F10.08	-0.031	-0.034	-0.046
SPAC4F10.11	-0.075	-0.034	-0.258
SPAC4F10.14c	-0.032	-0.029	-0.189
SPAC4F10.16c	-0.047	0.005	-0.769
SPAC4F10.17	-0.001	-0.025	-0.092
SPAC4F10.19c	0.107	0.138	-0.253
SPAC4F10.20	-0.025	0	-0.012
SPAC4F8.01	-0.187	-0.081	-1.101
SPAC4F8.08	0.034	0.037	-0.023
SPAC4F8.10c	0.02	-0.016	0.051
SPAC4F8.11	0.01	0.009	-0.075
SPAC4F8.15	0.008	0.007	0.124
SPAC4G9.02	0.04	0.016	-0.001
SPAC4G9.05	0.023	0.027	0.037
SPAC4G9.06c	0.049	-0.033	-0.228
SPAC4G9.09c	-0.107	-0.045	-1.284
SPAC4G9.11c	0.04	-0.008	0.156
SPAC4G9.12	0.017	0.098	0.014
SPAC4G9.14	0.077	0.037	0.025
SPAC4G9.16c	-0.172	-0.019	-1.039
SPAC4G9.19	-0.022	-0.122	0
SPAC4G9.20c	-0.026	-0.082	-0.188
SPAC4H3.01	-0.071	0.032	0.028
SPAC4H3.02c	0.007	0.035	0.087
SPAC4H3.03c	0.158	0.16	0.125
SPAC4H3.04c	-0.07	-0.024	0.253
SPAC4H3.05	0.071	0.072	0.131
SPAC4H3.06	-0.023	-0.011	0.089
SPAC4H3.07c	-0.042	-0.028	-0.92
SPAC4H3.14c	0.008	-0.003	0.17
SPAC513.01c	0.178	0.069	-0.22
SPAC513.02	-0.034	0.01	-0.068
SPAC513.03	-0.053	-0.993	-0.396
SPAC513.05	-0.069	-0.209	0.107
SPAC513.06c	-0.041	0.001	0.012
SPAC513.07	-0.063	0.022	-0.038
SPAC56E4.03	-0.042	-0.055	-0.215
SPAC56E4.06c	-0.064	0.007	-0.042
SPAC56E4.07	-0.056	-0.002	-0.019
SPAC56F8.05c	-0.115	-0.026	-0.124
SPAC56F8.06c	-0.121	0.019	-0.352
SPAC56F8.09	-0.056	0.105	-0.066
SPAC56F8.12	-0.107	0.093	0.107
SPAC56F8.14c	-0.063	0.022	-0.327
SPAC56F8.16	-0.099	0.027	-0.039
SPAC57A10.02	0.107	0.262	-1.692
SPAC57A10.03	-0.055	0.025	0.015
SPAC57A10.04	-0.038	0.023	0.057
SPAC57A10.06	-0.108	-0.054	-0.014
SPAC57A10.07	0.002	0.039	0.005
SPAC57A10.08c	-0.028	-0.161	-0.076
SPAC57A10.09c	0.014	0.118	0.205
SPAC57A10.10c	-0.01	0.073	0.195
SPAC57A10.14	0.047	-0.109	-0.614
SPAC57A7.04c	0.077	-0.498	0.541
SPAC57A7.05	-0.019	0.03	0.105
SPAC57A7.07c	-0.037	0.014	0.09
SPAC57A7.08	-0.076	0.093	0.135
SPAC57A7.09	0.029	-0.133	-0.798
SPAC57A7.12	NA	NA	NA
SPAC57A7.13	-0.074	0	0.072
SPAC589.02c	-0.106	-0.098	-0.16
SPAC589.03c	0.008	0.005	0.059

SPAC589.05c	0.01	0.004	-0.032
SPAC589.07c	0.047	-0.003	0.064
SPAC589.08c	0.043	0.057	0.2
SPAC589.09	-0.19	-0.041	-0.048
SPAC589.10c	0.036	-0.056	0.048
SPAC589.11	0.042	-0.021	-0.124
SPAC589.12	0.066	0.046	0.299
SPAC5D6.01	-0.036	-0.002	-0.168
SPAC5D6.02c	-0.065	0.207	-0.033
SPAC5D6.04	-0.014	0.027	0.147
SPAC5D6.05	-1.675	-0.226	0.01
SPAC5D6.06c	-0.134	-0.073	0.289
SPAC5D6.07c	-0.023	0.041	0.209
SPAC5D6.08c	0.008	0.056	0.036
SPAC5D6.09c	-0.063	-0.029	-0.247
SPAC5D6.10c	0.003	0.051	0.038
SPAC5D6.13	0.056	0.133	0.1
SPAC5H10.01	-0.006	-0.011	0.39
SPAC5H10.02c	-0.031	-0.083	0.153
SPAC5H10.04	-0.306	-0.479	-0.856
SPAC5H10.05c	-0.041	0.021	0.068
SPAC5H10.06c	0.029	0.179	0.071
SPAC5H10.07	0	-0.002	0.053
SPAC5H10.08c	0.045	0.166	-0.107
SPAC5H10.09c	0.009	-0.001	-0.199
SPAC5H10.10	-0.001	0.039	-0.022
SPAC5H10.11	-0.039	-0.001	-0.268
SPAC5H10.12c	-0.036	-0.013	0.146
SPAC5H10.13c	-0.013	0.01	-0.218
SPAC607.02c	0.07	-0.303	-0.633
SPAC607.06c	-0.023	-0.004	0.118
SPAC607.07c	-0.032	0.06	0.045
SPAC607.08c	0.068	-0.057	0.084
SPAC607.09c	0.028	0.238	0.246
SPAC607.10	0.086	0.033	0.152
SPAC630.04c	-0.192	-0.218	-0.806
SPAC630.05	0.03	0.044	-0.295
SPAC630.06c	-0.023	-0.003	0.13
SPAC630.07c	-0.075	-0.035	-0.112
SPAC630.09c	-0.025	-0.086	-0.064
SPAC630.10	-0.063	0.057	-0.188
SPAC630.11	-0.227	-0.228	-0.216
SPAC630.13c	0.019	0.003	-0.334
SPAC630.15	0.029	-0.027	0.091
SPAC631.01c	0.046	-0.016	-0.227
SPAC631.02	0.097	0.174	0.196
SPAC637.03	0.012	-0.032	-0.12
SPAC637.06	0.188	0.581	0.496
SPAC637.10c	-0.183	0.005	-0.771
SPAC637.11	-0.395	-0.547	-1.133
SPAC637.13c	0.035	0.09	0.3
SPAC644.08	0.018	0.067	0.081
SPAC644.09	-0.038	-0.048	-0.012
SPAC644.11c	0.083	-0.017	-0.271
SPAC644.13c	-0.027	-0.016	0.306
SPAC644.15	-0.195	-0.013	-0.061
SPAC652.01	0.156	0.213	-0.301
SPAC664.01c	-0.055	-0.823	-0.959
SPAC664.02c	0.233	-0.069	0.298
SPAC664.03	-0.276	-0.03	-0.11
SPAC664.04c	-0.042	0.04	-0.233
SPAC664.07c	0.021	-0.012	0.2
SPAC664.10	0.018	0.072	0.054
SPAC664.12c	0.005	-0.001	0.289
SPAC664.13	0.024	0.051	0.003
SPAC664.14	-0.04	-0.029	0.051
SPAC664.15	0.044	0.081	0.145
SPAC683.02c	-0.059	0.028	-0.094
SPAC683.03	-0.087	0.039	0.187
SPAC688.03c	-0.032	0.03	0.115
SPAC688.04c	-0.065	0.04	0.108
SPAC688.06c	0.005	-0.013	-0.009
SPAC688.10	0.009	0.022	0.001

SPAC688.12c	-0.074	0.065	0.065
SPAC688.13	0.03	0.157	0.166
SPAC688.14	0.027	0.016	0.1
SPAC694.02	0.005	-0.028	-0.007
SPAC694.03	-0.033	-0.044	0.236
SPAC694.04c	-0.036	-0.182	-0.118
SPAC694.05c	-0.025	0.086	0.107
SPAC694.06c	-0.018	0.04	0.08
SPAC6B12.02c	NA	NA	NA
SPAC6B12.03c	-0.034	-0.02	-0.124
SPAC6B12.04c	0.024	-0.054	-0.165
SPAC6B12.05c	-0.017	-0.058	-0.151
SPAC6B12.06c	0	-0.075	0.116
SPAC6B12.07c	-0.056	0.095	-0.196
SPAC6B12.08	-0.011	0.061	-0.293
SPAC6B12.09	-0.125	-0.08	-0.037
SPAC6B12.12	-0.037	0.015	0.065
SPAC6B12.14c	-0.049	0.04	-0.117
SPAC6B12.16	0.01	0.027	0.037
SPAC6C3.02c	0.049	0.06	0.285
SPAC6C3.03c	0.042	0.037	-0.009
SPAC6C3.04	-0.081	0.138	-0.431
SPAC6C3.05	0.011	-0.036	-0.172
SPAC6C3.06c	-0.032	-0.079	0.006
SPAC6C3.07	-0.043	0.013	0.12
SPAC6C3.08	-0.061	-0.002	-0.107
SPAC6F12.02	0.104	0.044	-0.176
SPAC6F12.03c	-0.502	-0.81	-1.06
SPAC6F12.04	0.015	0.009	-0.106
SPAC6F12.06	0.01	0.136	-0.039
SPAC6F12.09	-0.038	-0.157	-0.291
SPAC6F12.12	-0.015	-0.001	-0.04
SPAC6F6.01	0.436	0.387	0.543
SPAC6F6.02c	0.051	-0.049	-0.006
SPAC6F6.03c	-0.075	0.153	0.188
SPAC6F6.04c	-0.057	-0.006	0.072
SPAC6F6.06c	-0.118	-0.096	0.025
SPAC6F6.09	0.559	0.752	0.12
SPAC6F6.11c	-0.056	0.126	-0.083
SPAC6F6.12	NA	NA	NA
SPAC6F6.13c	0.049	0.04	0.103
SPAC6F6.17	-0.132	-0.071	-0.216
SPAC6G10.02c	0.036	0.034	0.111
SPAC6G10.03c	-0.079	-0.093	0.05
SPAC6G10.06	-0.003	-0.09	0.042
SPAC6G10.08	-0.091	-0.016	0.052
SPAC6G10.10c	-0.006	0.061	0.123
SPAC6G10.11c	NA	0.309	NA
SPAC6G10.12c	0.054	0.027	0.022
SPAC6G9.01c	-0.006	0.025	0.057
SPAC6G9.03c	-0.013	-0.012	0.087
SPAC6G9.04	-0.029	-0.021	-0.034
SPAC6G9.05	-0.012	-0.001	-0.054
SPAC6G9.08	0.038	0.037	0.032
SPAC6G9.09c	-0.005	0.023	-0.138
SPAC6G9.10c	-0.017	-0.012	-0.507
SPAC6G9.12	-0.178	0.098	0.119
SPAC6G9.13c	0.022	0.019	0.094
SPAC6G9.14	-0.322	-0.121	-0.265
SPAC6G9.15c	0.033	-0.083	0.107
SPAC6G9.16c	0.038	0.05	0.034
SPAC732.02c	-0.169	0.043	0.186
SPAC750.05c	-0.044	-0.211	-0.027
SPAC750.06c	-0.013	-0.058	0.061
SPAC750.08c	0.039	-0.063	-0.72
SPAC767.01c	0.029	-0.045	0.065
SPAC7D4.02c	0.016	-0.01	-0.011
SPAC7D4.03c	-0.03	-0.481	-0.874
SPAC7D4.05	-0.019	-0.009	0.154
SPAC7D4.06c	0.148	0.138	-0.519
SPAC7D4.08	0.039	0.029	0.243
SPAC7D4.12c	-1.248	0.044	-2
SPAC7D4.13c	0.118	-0.002	-0.472

SPAC7D4.14c	-0.012	0.143	0.076
SPAC806.03c	-0.12	-0.078	-0.515
SPAC806.04c	-0.169	-0.017	0.202
SPAC806.07	0.081	-0.001	-0.033
SPAC806.08c	-0.067	-0.037	0.008
SPAC823.02	-0.029	-0.039	-0.124
SPAC823.03	0.03	-0.191	-1.055
SPAC823.09c	-0.009	0.041	0.197
SPAC823.10c	0.238	0.349	0.518
SPAC823.11	-0.027	-0.085	-0.605
SPAC823.13c	-0.017	-0.018	-0.165
SPAC823.14	0.004	-0.008	0.097
SPAC823.15	0.07	-0.005	0.057
SPAC823.16c	0.03	-0.008	-0.075
SPAC824.02	NA	NA	NA
SPAC824.03c	-0.017	-0.001	-0.116
SPAC824.05	-0.023	-0.048	0.267
SPAC824.07	-0.013	-0.231	0.015
SPAC824.08	-0.002	0.008	-0.054
SPAC869.01	-0.009	0.009	0.003
SPAC869.02c	-0.057	-0.04	0.128
SPAC869.03c	0	-0.023	-0.025
SPAC869.04	0.019	0.019	0.058
SPAC869.05c	-0.01	-0.002	0.114
SPAC869.06c	-0.029	0.043	-0.238
SPAC869.07c	0.031	-0.006	0.078
SPAC869.08	-0.017	0.053	0.119
SPAC869.09	0.023	0.081	0.115
SPAC869.10c	0.026	-0.012	0.118
SPAC869.11	-0.086	-0.083	-0.25
SPAC890.02c	0.107	0.106	0.013
SPAC890.03	-0.078	-0.023	0.065
SPAC890.05	0.057	0.107	-0.132
SPAC890.06	-0.056	0.066	0.049
SPAC890.07c	0.016	-0.192	-0.261
SPAC8C9.04	-0.038	-0.017	0.111
SPAC8C9.05	-0.013	-0.031	-0.294
SPAC8C9.09c	0.053	-0.035	0.075
SPAC8C9.11	0.088	0.008	-0.361
SPAC8C9.12c	-0.009	0.008	-0.057
SPAC8C9.14	0.093	0.006	0.147
SPAC8C9.16c	0.042	-0.001	0.016
SPAC8C9.17c	-0.034	0.013	-0.075
SPAC8C9.19	0.084	-0.249	0.139
SPAC8E11.02c	0.098	0.014	NA
SPAC8E11.03c	0.055	-0.014	0.176
SPAC8E11.04c	-0.015	-0.122	0.099
SPAC8E11.05c	0.049	0.047	-0.768
SPAC8E11.06	-0.029	0.008	-0.008
SPAC8E11.10	-0.11	-0.035	0.076
SPAC8F11.02c	0.385	0.485	NA
SPAC8F11.03	0.063	0.045	0.066
SPAC8F11.05c	-0.038	-0.016	0.316
SPAC8F11.08c	-0.019	-0.033	-0.041
SPAC8F11.09c	-0.128	-0.024	-0.29
SPAC8F11.10c	-0.042	0.04	-0.36
SPAC9.02c	-0.083	-0.009	-0.17
SPAC9.05	0.053	0.049	-0.096
SPAC9.06c	-0.007	0.036	-0.142
SPAC9.07c	-0.03	-0.106	-0.004
SPAC9.08c	0.331	0.293	-0.692
SPAC9.10	0.019	0.039	0.093
SPAC9.11	-0.022	-0.055	-0.044
SPAC9.12c	0.105	-0.013	0.117
SPAC922.03	-0.016	0.014	0.097
SPAC922.04	0.032	-0.076	0.016
SPAC922.05c	-0.066	-0.339	-0.652
SPAC922.06	0.066	0.092	0.084
SPAC922.07c	-0.011	0.051	-0.006
SPAC926.02	0.052	0.093	0.187
SPAC926.03	-0.444	-0.402	-0.774
SPAC926.05c	-0.077	0.019	-0.095
SPAC926.06c	0.127	-0.402	-0.132

SPAC926.07c	0.029	0.038	0.08
SPAC926.09c	0.012	0.022	0.134
SPAC959.04c	0.073	-0.231	0.492
SPAC959.05c	-0.127	-0.113	0.062
SPAC959.07	0.037	0.09	-0.112
SPAC959.08	0.127	NA	-0.054
SPAC977.05c	0.031	0.029	0.074
SPAC977.06	-0.132	-0.096	-0.083
SPAC977.10	-0.052	0.176	0.286
SPAC977.11	-0.035	0.127	0.243
SPAC977.14c	-0.115	-0.084	-0.04
SPAC977.15	0.041	0.043	0.07
SPAC977.16c	-0.01	0.027	0.124
SPAC977.17	0.036	0.016	-0.198
SPAC9E9.03	0.014	-0.012	0.162
SPAC9E9.05	-0.032	-0.069	0.041
SPAC9E9.08	0.273	0.124	0.446
SPAC9E9.09c	-0.057	-0.312	-1.22
SPAC9E9.10c	-0.064	0.07	-0.164
SPAC9E9.11	-0.006	-0.018	0.116
SPAC9E9.12c	0.041	0.033	0.05
SPAC9E9.13	-0.062	0.038	0.209
SPAC9E9.14	-0.1	-0.109	-0.275
SPAC9E9.15	0.069	-0.097	0.087
SPAC9G1.02	0.275	0.165	-0.957
SPAC9G1.04	0.052	0.064	0.046
SPAC9G1.05	-0.014	-0.066	-0.296
SPAC9G1.06c	0.308	0.34	0.341
SPAC9G1.07	-0.044	0.25	-0.933
SPAC9G1.08c	-0.001	0.024	0.182
SPAC9G1.10c	-0.053	-0.062	-0.162
SPAC9G1.11c	-0.04	0.045	-0.129
SPAC9G1.12	0.219	0.152	-0.04
SPACUNK12.02c	-0.028	-0.072	0.081
SPACUNK4.07c	0.083	-0.1	-0.159
SPACUNK4.08	-0.094	-0.019	0.04
SPACUNK4.09	-0.026	-0.058	0.102
SPACUNK4.10	-0.143	-0.139	0.024
SPACUNK4.11c	-0.041	-0.23	-0.782
SPACUNK4.13c	0.002	0.018	0.026
SPACUNK4.14	0.008	-0.038	0.148
SPACUNK4.15	0.07	0.145	-0.367
SPACUNK4.16c	-0.384	-0.035	0.02
SPACUNK4.17	0.03	0.068	0.086
SPACUNK4.19	0.084	-0.005	0.214
SPAP11E10.01	-0.025	-0.085	0.068
SPAP11E10.02c	0.032	0.032	0.087
SPAP14E8.02	-0.148	-0.059	0.11
SPAP14E8.04	0.017	0.106	0.117
SPAP14E8.05c	-0.012	0.122	0.221
SPAP27G11.02	-0.092	-0.018	-0.117
SPAP27G11.07c	0.032	0.003	-0.093
SPAP27G11.08c	-0.179	-0.004	-0.008
SPAP27G11.10c	-0.038	-0.134	0.138
SPAP27G11.12	-0.068	-0.025	0.174
SPAP27G11.15	-0.005	0.121	0.16
SPAP27G11.16	-0.003	-0.096	0.297
SPAP32A8.02	-0.04	-0.07	-0.483
SPAP32A8.03c	-0.026	0.022	0.017
SPAP7G5.03	0.026	0.094	0.042
SPAP7G5.04c	0.062	0.01	-0.498
SPAP7G5.05	0.028	0.048	0.037
SPAP8A3.02c	-0.055	0.029	-0.032
SPAP8A3.03	0.077	0.1	0.08
SPAP8A3.04c	0.024	-0.052	0.067
SPAP8A3.05	0.057	0.128	0.187
SPAP8A3.07c	0.078	NA	NA
SPAP8A3.12c	0.027	0.081	0.094
SPAP8A3.13c	-0.063	-0.045	-0.319
SPAPB17E12.02	-0.029	-0.087	-0.994
SPAPB17E12.03	-0.094	-0.344	-0.85
SPAPB17E12.05	NA	NA	NA
SPAPB17E12.08	-0.211	-0.203	0.024

SPAPB17E12.12c	0.028	-0.067	0.161
SPAPB17E12.14c	0.032	-0.086	-0.463
SPAPB18E9.04c	0.003	0.019	0.143
SPAPB1A10.03	-0.011	0.053	0.044
SPAPB1A10.05	-0.004	-0.041	-0.023
SPAPB1A10.07c	-0.156	-0.343	-1.209
SPAPB1A10.08	0.036	0.092	-0.084
SPAPB1A10.09	0.056	0.071	-0.104
SPAPB1A10.10c	0.037	-0.015	0.071
SPAPB1A10.12c	-0.011	-0.014	0.015
SPAPB1A10.13	-0.081	0.046	0.206
SPAPB1A10.14	0.002	0.004	-0.236
SPAPB1A10.15	0.058	-0.026	-0.033
SPAPB1A11.01	0.026	0.203	0.109
SPAPB1A11.02	-0.029	0.02	0.198
SPAPB1A11.03	-0.047	0.022	0.131
SPAPB1A11.04c	0.095	-0.039	0.063
SPAPB1E7.04c	-0.017	-0.006	0.066
SPAPB1E7.05	-0.054	-0.027	0.02
SPAPB1E7.06c	0.258	0.275	NA
SPAPB1E7.07	0.085	-0.021	0.235
SPAPB1E7.08c	0.003	-0.028	0.171
SPAPB1E7.12	0.391	NA	NA
SPAPB21F2.02	-0.004	0.036	0.003
SPAPB21F2.03	0.11	0.231	-0.036
SPAPB24D3.01	0.041	0.047	-0.086
SPAPB24D3.02c	-0.166	-0.074	-0.134
SPAPB24D3.03	-0.026	-0.009	0.137
SPAPB24D3.04c	-0.084	-0.074	0.044
SPAPB24D3.07c	-0.004	0.045	0.121
SPAPB24D3.08c	-0.079	-0.018	0.002
SPAPB24D3.09c	-0.101	-0.093	-0.136
SPAPB24D3.10c	0.036	0.06	0.034
SPAPB2B4.02	0.047	-0.158	-0.488
SPAPB2B4.03	-0.121	0.027	-0.003
SPAPB2B4.04c	0.014	-0.014	-0.002
SPAPB2B4.06	0.042	0.043	-0.042
SPAPB2B4.07	0.07	0.015	-0.243
SPAPB2C8.01	-0.029	-0.034	-0.016
SPAPB8E5.02c	0.001	0.044	-0.272
SPAPB8E5.03	0.013	0.014	0.136
SPAPB8E5.04c	0.048	0.162	-0.104
SPAPB8E5.05	0.029	0.002	-0.313
SPAPB8E5.06c	0.07	0.014	0.216
SPAPB8E5.08	-0.031	-0.045	-0.069
SPAPB8E5.10	-0.036	0.056	0.054
SPAPJ691.02	0.011	0.043	0.038
SPAPJ691.03	-0.072	0.076	0.04
SPAPJ695.01c	0.044	0.119	0.035
SPAPJ696.02	-0.306	0.202	-0.115
SPAPJ760.02c	0.023	-0.196	-0.044
SPAPJ760.03c	0.034	0.182	0.114
SPAPYUG7.02c	0.091	0.019	-0.752
SPAPYUG7.03c	-0.019	0.045	-0.121
SPAPYUG7.04c	-0.039	-0.015	-0.004
SPAPYUG7.06	0.004	0.154	0.047
SPAPYUK71.03c	0.525	0.602	0.471
SPBC106.01	-0.029	-0.069	-0.282
SPBC106.02c	0.524	0.031	-0.211
SPBC106.03	0.102	0.053	0.067
SPBC106.04	-0.108	0.108	0.561
SPBC106.05c	0.012	-0.037	-0.052
SPBC106.08c	-0.06	-0.015	0.007
SPBC106.10	0.299	NA	0.164
SPBC106.11c	-0.042	0.048	-0.042
SPBC106.12c	-0.002	-0.045	-0.033
SPBC106.16	-0.284	0.051	0.426
SPBC106.17c	0.129	NA	NA
SPBC106.20	-0.068	-0.402	-0.197
SPBC1105.01	-0.01	0.101	0.012
SPBC1105.02c	0.008	-0.157	-0.514
SPBC1105.05	-0.064	-0.058	0.015
SPBC1105.08	-0.064	-0.001	0.04

SPBC1105.09	0.04	0.043	0.182
SPBC1105.10	NA	NA	NA
SPBC1105.11c	0.015	0.035	0.116
SPBC1105.12	-0.089	0.086	0.202
SPBC1105.18c	-0.072	-0.011	0.115
SPBC115.02c	-0.079	0.012	0.101
SPBC115.03	0.034	0.009	-0.034
SPBC119.03	0.014	-0.02	0.143
SPBC119.04	-0.223	-0.19	-0.37
SPBC119.08	0.011	0.095	-0.4
SPBC119.12	0.406	0.313	NA
SPBC119.14	-0.116	-0.071	-0.024
SPBC119.16c	0.038	0.023	-0.477
SPBC1198.01	-0.041	0.017	0.074
SPBC1198.03c	0.095	0.054	0.004
SPBC1198.06c	0.028	0.025	-0.263
SPBC1198.07c	-0.054	0.046	0.096
SPBC1198.08	-0.008	-0.065	-0.061
SPBC1198.09	-0.063	-0.022	-0.124
SPBC1198.11c	-0.043	0.123	-0.893
SPBC1198.12	0.079	0.032	0.015
SPBC1198.14c	0.02	-0.022	0.052
SPBC11B10.02c	-0.049	-0.048	0
SPBC11B10.05c	-0.001	0.032	-0.089
SPBC11B10.06	0.168	0.106	-0.024
SPBC11B10.07c	0.047	0.032	0.2
SPBC11B10.08	-0.026	0.025	0.083
SPBC11B10.10c	-0.082	-0.249	-0.814
SPBC11C11.01	0.053	0.018	0.116
SPBC11C11.06c	-0.027	0.061	0.218
SPBC11C11.07	-0.375	0.032	-0.59
SPBC11C11.08	0.034	0.132	0.092
SPBC11C11.09c	NA	0.081	-0.1
SPBC11C11.10	-0.191	-0.61	-1.084
SPBC11C11.11c	0.021	0.032	0.054
SPBC11G11.01	-0.149	-0.021	-0.401
SPBC11G11.02c	0.051	0.163	-0.207
SPBC11G11.03	0.018	-0.033	-0.03
SPBC11G11.05	-0.011	0.207	0.19
SPBC1215.01	-0.043	-0.073	-0.296
SPBC1271.01c	0.004	0.007	0.044
SPBC1271.03c	-0.056	-0.108	-0.078
SPBC1271.05c	0.01	0.038	-0.01
SPBC1271.06c	0.02	0.019	0.094
SPBC1271.07c	-0.042	-0.046	0.1
SPBC1271.08c	0.004	-0.033	0.163
SPBC1271.09	-0.032	-0.017	-0.277
SPBC1271.10c	-0.019	0.067	-0.096
SPBC1271.11	0.01	0.074	0.208
SPBC1271.12	-0.52	-0.042	-1.048
SPBC1271.14	-0.062	-0.236	-0.508
SPBC1289.01c	-0.031	-0.111	0.131
SPBC1289.09	-0.008	-0.046	-0.353
SPBC1289.11	-0.013	0.032	0.022
SPBC1289.13c	-0.012	0.002	0.08
SPBC1289.14	-0.118	-0.416	-0.885
SPBC1289.15	-0.06	-0.074	-0.06
SPBC1289.16c	-0.105	-0.077	0.02
SPBC12C2.01c	0.047	0.004	-0.033
SPBC12C2.02C	0.487	0.003	0.212
SPBC12C2.03c	-0.161	-0.098	-0.213
SPBC12C2.04	0.039	0.007	-0.019
SPBC12C2.05c	0.066	0.056	0.038
SPBC12C2.07c	0.029	0.051	0.01
SPBC12C2.08	0.015	0.082	-0.04
SPBC12C2.09c	-0.023	-0.213	0.116
SPBC12C2.12c	0.036	0.06	0.06
SPBC12D12.04c	0.024	-1.363	-0.961
SPBC12D12.05c	0.027	0.103	0.237
SPBC12D12.06	-0.038	-0.169	-0.277
SPBC12D12.07c	-0.227	-0.023	-0.762
SPBC12D12.09	-0.001	-0.066	-0.171
SPBC1306.02	0.04	-0.314	-0.307

SPBC1347.01c	0.03	-0.025	-0.022
SPBC1347.02	-0.164	-0.043	-0.105
SPBC1347.03	0.027	-0.005	0.058
SPBC1347.06c	-0.013	0.007	0.331
SPBC1347.07	-0.022	-0.011	0.073
SPBC1347.08c	-0.066	-0.013	-0.402
SPBC1347.09	0	-0.011	0.123
SPBC1347.11	-0.038	0.049	0.041
SPBC1347.12	0.044	0.003	0.027
SPBC1347.13c	-0.011	0.094	-0.052
SPBC1348.01	-0.03	0.014	0.078
SPBC1348.02	-0.082	-0.079	-0.388
SPBC1348.14c	0.05	-0.038	0.135
SPBC13A2.02	0.027	-0.021	0.115
SPBC13A2.04c	-0.054	0.168	-0.012
SPBC13E7.03c	0.01	0.131	0.122
SPBC13E7.06	0.051	0.084	0.263
SPBC13E7.07	-0.007	0.091	0.124
SPBC13E7.08c	-0.058	0.017	0.05
SPBC13E7.09	0.033	0.045	0.087
SPBC13E7.11	-0.091	-0.281	-1.158
SPBC13G1.02	0.006	0.032	0.054
SPBC13G1.03c	-0.034	-0.06	-0.38
SPBC13G1.04c	0.04	0.017	0.177
SPBC13G1.08c	-0.386	-0.024	-0.811
SPBC13G1.10c	0.611	0.586	0.407
SPBC13G1.12	-0.271	-0.6	-0.802
SPBC13G1.14c	-0.078	-0.178	-0.039
SPBC146.02	0.036	0.019	0.289
SPBC146.04	-0.034	-0.061	-0.078
SPBC146.06c	-0.002	-0.017	0.133
SPBC146.09c	-0.014	-0.087	0.066
SPBC146.10	0.002	0.048	0.217
SPBC146.11c	-0.014	0.039	0.135
SPBC14C8.03	-0.112	-0.088	0.067
SPBC14C8.04	0.096	0.088	0.17
SPBC14C8.05c	-0.049	-0.035	0.002
SPBC14C8.09c	-0.015	0.049	0.079
SPBC14C8.11c	0.025	0.057	0.025
SPBC14C8.15	-0.062	-0.066	-0.13
SPBC14C8.16c	-0.303	0.392	0.004
SPBC14F5.03c	0.001	0.022	0.147
SPBC14F5.07	0.056	0.003	-0.285
SPBC14F5.09c	0.062	-0.02	-0.081
SPBC14F5.10c	0.366	-0.243	-0.863
SPBC14F5.11c	-0.153	-0.03	-0.03
SPBC14F5.13c	-0.037	0.048	-0.262
SPBC1539.02	0.045	0.076	0.111
SPBC1539.03c	0.108	0.128	0.192
SPBC1539.04	0.053	0.043	0.003
SPBC1539.06	0.246	0.868	0.945
SPBC1539.07c	-0.029	-0.039	0.039
SPBC1539.08	-0.002	0.126	-1.178
SPBC1539.10	0.067	-0.299	-0.79
SPBC15C4.01c	-0.977	0.14	-0.284
SPBC15C4.04c	0.016	0.033	-0.228
SPBC15C4.05	-0.023	-0.259	-0.599
SPBC15C4.06c	-0.05	0	0.072
SPBC15D4.01c	0.031	0.157	-0.007
SPBC15D4.03	-0.14	0.083	-0.019
SPBC15D4.05	-0.004	-0.154	-0.285
SPBC15D4.06	0.034	0.077	0.087
SPBC15D4.07c	-0.053	-0.44	-0.044
SPBC15D4.09c	0.081	0.029	-0.184
SPBC15D4.10c	-0.315	-0.08	-0.714
SPBC15D4.12c	0.018	-0.002	0.027
SPBC15D4.13c	0.025	-0.061	0.107
SPBC15D4.15	0.025	-0.05	-0.71
SPBC1604.01	0.015	0.002	0.058
SPBC1604.02c	-0.355	-0.036	-0.774
SPBC1604.04	-0.064	-0.283	-0.189
SPBC1604.08c	-0.3	-2	-1.525
SPBC1604.09c	-0.015	-0.178	-0.451

SPBC1604.12	0.077	0.059	0.056
SPBC1604.16c	-0.011	-0.003	0.091
SPBC1604.18c	0.077	-0.032	0.005
SPBC1604.19c	0.066	0.033	0.128
SPBC1604.20c	0.202	0.382	-0.016
SPBC1652.01	-0.061	0.091	0.128
SPBC1683.01	-0.015	0.116	0.154
SPBC1683.02	0	-0.023	0.108
SPBC1683.03c	0	0.047	0.227
SPBC1683.04	-0.002	0.045	0.138
SPBC1683.06c	-0.074	0.01	0.055
SPBC1683.07	-0.036	-0.011	0.194
SPBC1683.08	0.002	0.03	0.283
SPBC1683.09c	-0.025	-0.605	-0.169
SPBC1683.10c	-0.006	0.062	0.097
SPBC1683.11c	-0.143	-0.045	-0.213
SPBC1683.12	0.055	0.057	0.124
SPBC1683.13c	-0.005	-0.017	-0.025
SPBC1685.01	0.227	0.145	0.288
SPBC1685.02c	-0.03	-0.955	-0.527
SPBC1685.04	0.048	0.17	0.264
SPBC1685.05	0.024	0.017	0.053
SPBC1685.06	-0.052	-0.031	0.066
SPBC1685.07c	-0.033	-0.042	-0.601
SPBC1685.08	-0.017	-0.187	-0.774
SPBC1685.11	-0.01	-0.009	-0.098
SPBC1685.13	0.147	-0.001	-0.99
SPBC1685.14c	0.09	0.052	0.212
SPBC1685.15c	-0.054	0.012	0.159
SPBC16A3.01	-0.054	-0.42	0.297
SPBC16A3.02c	-0.008	-0.044	0.034
SPBC16A3.03c	-0.197	-0.254	-0.577
SPBC16A3.06	-0.008	-0.005	0.144
SPBC16A3.07c	0.565	0.427	NA
SPBC16A3.08c	-0.717	-0.672	-1.68
SPBC16A3.10	0.085	-0.311	-0.25
SPBC16A3.12c	0.043	0.031	0.102
SPBC16A3.13	-0.01	0	0.061
SPBC16A3.14	-0.105	-0.041	-0.127
SPBC16A3.16	-0.127	-0.153	-0.189
SPBC16A3.17c	0.277	0.424	NA
SPBC16A3.18	0.071	0.145	-0.357
SPBC16A3.19	-0.296	-0.105	-0.956
SPBC16C6.01c	-0.028	-0.157	0.141
SPBC16C6.03c	-0.034	0.013	-0.057
SPBC16C6.04	0.004	-0.054	0.058
SPBC16C6.05	0.057	-0.018	-0.117
SPBC16C6.06	0.006	-0.274	-0.358
SPBC16C6.08c	0.028	-0.053	-0.016
SPBC16C6.10	0.008	-0.021	-0.021
SPBC16D10.01c	-0.081	-0.58	-0.466
SPBC16D10.02	-0.022	-0.026	0.147
SPBC16D10.05	-0.133	-0.129	-0.152
SPBC16D10.07c	-0.119	-0.22	-0.348
SPBC16D10.08c	-0.209	-0.177	0.089
SPBC16D10.11c	-0.016	-0.061	-0.066
SPBC16E9.03c	-0.15	0.076	0.052
SPBC16E9.06c	0.057	0.016	-0.011
SPBC16E9.07	0.047	-0.005	0.098
SPBC16E9.08	0.089	-0.036	-0.178
SPBC16E9.09c	-0.226	-0.83	-0.694
SPBC16E9.11c	0.008	-0.037	0.21
SPBC16E9.12c	-0.026	-0.094	-1.55
SPBC16E9.13	0.118	0.104	0.019
SPBC16E9.15	0.113	-0.054	0.092
SPBC16E9.16c	0.016	-0.061	-0.577
SPBC16E9.17c	0.011	-0.056	-0.161
SPBC16E9.18	0.065	0.538	0.592
SPBC16E9.19	0.064	0.098	0.04
SPBC16G5.02c	0.068	-0.137	0.172
SPBC16G5.03	-0.033	0.121	0.244
SPBC16G5.05c	-0.13	-0.033	-0.261
SPBC16G5.06	-0.113	-0.044	0.093

SPBC16G5.07c	0.017	0.089	-0.082
SPBC16G5.09	0.04	0.088	0.041
SPBC16G5.11c	-0.099	-0.011	-0.334
SPBC16G5.13	-0.338	0.048	-0.212
SPBC16G5.15c	-0.044	0.008	-0.045
SPBC16G5.16	-0.019	-0.034	-0.025
SPBC16G5.17	0.063	0.014	0.145
SPBC16H5.02	0.039	0.075	0.134
SPBC16H5.04	0.011	-0.113	0.041
SPBC16H5.05c	-0.057	0.021	0.059
SPBC16H5.06	0.411	0.218	-0.828
SPBC16H5.07c	-0.343	-0.445	-0.585
SPBC16H5.08c	0.096	-0.163	-0.54
SPBC16H5.11c	-0.071	-0.021	-0.01
SPBC16H5.12c	0.053	0.01	-0.772
SPBC16H5.13	-0.315	-0.179	-2
SPBC16H5.14c	-0.043	-0.037	-0.065
SPBC1703.03c	-0.034	0.245	-0.02
SPBC1703.04	-0.051	-0.048	-0.716
SPBC1703.06	0.017	-0.021	-0.257
SPBC1703.07	-0.048	0.109	-0.156
SPBC1703.08c	0.075	-0.095	-0.035
SPBC1703.11	0.051	0.012	0.133
SPBC1703.12	-0.049	-0.1	-0.305
SPBC1703.14c	0.004	-0.038	-0.198
SPBC1706.01	0.061	-0.07	0.076
SPBC1706.03	-0.11	-0.175	-0.108
SPBC1709.01	-0.042	0.007	0.036
SPBC1709.04c	-0.092	0.007	-0.48
SPBC1709.06	0.016	0.068	0.125
SPBC1709.09	-0.118	-1.14	-1.472
SPBC1709.11c	-0.105	-0.033	0.009
SPBC1709.12	0.071	0.213	0.29
SPBC1709.13c	-0.016	0.023	-0.291
SPBC1709.14	0.021	-0.021	-0.223
SPBC1709.16c	-0.019	-0.056	0.092
SPBC1709.18	-0.012	-0.149	-1.162
SPBC1709.19c	0.053	0.175	0.252
SPBC1711.01c	0.016	0.051	-0.146
SPBC1711.03	0.757	0.72	0.619
SPBC1711.04	-0.043	0.037	0.057
SPBC1711.05	0.028	0.046	-0.073
SPBC1711.06	-0.08	-0.067	0.076
SPBC1711.08	-0.095	0.042	-0.184
SPBC1711.09c	-0.014	0.002	-0.02
SPBC1711.11	0.059	0.047	0.133
SPBC1711.12	0.012	0.033	-0.219
SPBC1711.13	-0.071	-0.367	-0.141
SPBC1711.14	0.19	0.278	0.211
SPBC1711.15c	0.04	0.097	-0.396
SPBC1718.02	-0.008	-0.038	0.047
SPBC1718.03	-0.521	-0.325	-0.333
SPBC1734.05c	0.012	0.116	0.011
SPBC1734.06	-0.215	-0.471	-0.305
SPBC1734.07c	-0.082	-0.075	-0.004
SPBC1734.08	-0.328	-0.093	-1.077
SPBC1734.09	-0.023	-0.05	-0.038
SPBC1734.11	-0.091	-0.054	-0.196
SPBC1734.12c	0.118	0.226	-0.049
SPBC1734.13	0.13	0.218	-0.066
SPBC1734.15	0.006	0.042	0.129
SPBC1773.01	0.041	0.042	-0.165
SPBC1773.02c	0.006	0.035	0.009
SPBC1773.03c	-0.052	-0.051	-0.016
SPBC1773.04	-0.002	-0.02	0.117
SPBC1773.05c	0.015	-0.07	0.086
SPBC1773.06c	-0.039	-0.064	0.069
SPBC1773.08c	-0.01	-0.004	0.112
SPBC1773.09c	-0.001	0.041	0.07
SPBC1773.12	0.015	0.09	0.053
SPBC1773.13	-0.003	0.022	0
SPBC1773.14	0.035	-0.174	-0.001
SPBC1773.15	-0.118	0.136	0.088

SPBC1773.16c	0.036	0.047	0.069
SPBC1773.17c	0.012	0.052	0.202
SPBC1778.02	-0.005	-0.032	0.142
SPBC1778.03c	-0.008	-0.016	-0.055
SPBC1778.04	-0.07	-0.033	-0.15
SPBC1778.05c	-0.086	-0.455	-0.955
SPBC1778.06c	0.159	-0.618	-0.784
SPBC1778.07	0.035	0.153	0.133
SPBC1778.09	-0.015	-0.037	-0.093
SPBC1778.10c	0.002	-0.055	0.149
SPBC17A3.02	0.062	0	0.123
SPBC17A3.03c	0.082	0.146	0.268
SPBC17A3.06	-0.005	0.055	-0.637
SPBC17A3.08	-0.009	-0.027	0.124
SPBC17A3.10	-0.057	-0.148	-1.075
SPBC17D1.02	-0.08	-0.065	-0.639
SPBC17D1.05	0.039	0.005	0.06
SPBC17D1.06	-0.013	0.033	-0.157
SPBC17D1.07c	0.014	-0.006	0.032
SPBC17D11.01	0.051	0.08	0.044
SPBC17D11.02c	-0.146	-0.113	0.094
SPBC17D11.03c	0.055	0.073	0.222
SPBC17D11.04c	-0.039	-0.015	-0.306
SPBC17D11.08	-0.119	-0.21	-0.959
SPBC17F3.01c	0.02	0.044	0.129
SPBC17G9.02c	-0.191	0.118	-0.132
SPBC17G9.05	-0.053	-0.045	-0.03
SPBC17G9.08c	-0.072	-0.503	-0.102
SPBC17G9.09	0.044	0.199	0.249
SPBC17G9.10	0.098	0.034	0.036
SPBC17G9.12c	0.005	-0.045	-0.056
SPBC1815.01	0.03	0.033	-0.266
SPBC1861.01c	NA	NA	NA
SPBC1861.02	0.064	0.048	0.044
SPBC1861.03	0.09	-0.011	-0.021
SPBC1861.06c	-0.047	-0.004	0.09
SPBC1861.07	-0.055	0.097	-0.038
SPBC1861.09	-0.051	0.013	-0.151
SPBC18A7.01	-0.014	-0.011	-0.068
SPBC18A7.02c	-0.008	-0.015	-0.104
SPBC18E5.01	-0.017	0.004	-0.067
SPBC18E5.07	-0.032	0.001	0.055
SPBC18E5.08	0.013	-0.004	-0.001
SPBC18E5.10	-0.052	-0.072	0.005
SPBC18E5.11c	-0.026	-0.013	-0.138
SPBC18E5.14c	-0.013	-0.218	0.108
SPBC18H10.05	0.031	0.043	-0.044
SPBC18H10.07	0.037	0.012	0.032
SPBC18H10.08c	-0.062	0.013	0.131
SPBC18H10.09	-0.002	0.004	0.109
SPBC18H10.10c	-0.003	-0.053	-0.236
SPBC18H10.11c	-0.675	-0.388	-0.62
SPBC18H10.13	-0.037	0.01	0.085
SPBC18H10.15	-0.1	-0.017	0.033
SPBC18H10.16	0.008	0.005	-0.065
SPBC18H10.18c	0.014	0.049	0.08
SPBC18H10.19	0.089	-0.236	-0.264
SPBC18H10.20c	0.041	0.051	0.197
SPBC1921.01c	-0.052	-0.086	-0.053
SPBC1921.03c	0.063	0.11	0.056
SPBC1921.04c	-0.097	0.058	-0.028
SPBC1921.05	0.088	0.027	0.235
SPBC1921.06c	-0.007	-0.041	-0.028
SPBC19C2.02	-0.024	-0.028	0.054
SPBC19C2.04c	0.013	0.001	0.103
SPBC19C2.06c	0.01	-0.01	0.153
SPBC19C2.09	0.001	-0.171	-0.155
SPBC19C2.10	0.009	0.065	0.26
SPBC19C2.13c	0.037	0.001	0.129
SPBC19C7.01	-0.046	-0.044	-0.387
SPBC19C7.02	-0.262	-0.049	-0.66
SPBC19C7.05	0.02	0.063	0.171
SPBC19C7.08c	0.001	-0.007	0.141

SPBC19C7.09c	0.016	0.095	0.184
SPBC19C7.10	0.078	0.14	0.014
SPBC19C7.11	0.084	0.178	0.284
SPBC19C7.12c	-0.067	-0.038	0.059
SPBC19F5.01c	-0.059	-0.078	-0.076
SPBC19F8.01c	0.097	-0.017	0.065
SPBC19F8.02	0.072	0.062	0.207
SPBC19F8.03c	-0.024	0.167	0.298
SPBC19F8.04c	0.006	-0.034	-0.175
SPBC19F8.06c	0.27	-0.465	-0.341
SPBC19F8.08	0.159	-0.003	-0.492
SPBC19G7.01c	-0.01	-0.127	-0.178
SPBC19G7.02	-0.061	-0.394	-0.209
SPBC19G7.03c	-0.299	0.07	0.068
SPBC19G7.07c	-0.566	-0.203	0.28
SPBC19G7.08c	-0.07	-0.003	0.081
SPBC19G7.18c	0.002	-0.197	0.017
SPBC1A4.02c	-0.014	0.101	0.074
SPBC1A4.04	0.052	-0.385	-0.624
SPBC1A4.05	-0.044	0.101	-0.085
SPBC1D7.01	0.034	0.026	0.042
SPBC1D7.03	0.085	0.047	0.137
SPBC1D7.05	NA	NA	NA
SPBC1E8.02	-0.039	-0.116	-0.195
SPBC20F10.02c	-0.087	-0.032	0.062
SPBC20F10.03	-0.005	0.007	-0.013
SPBC20F10.05	-0.075	-0.071	-0.694
SPBC20F10.06	0.048	-0.032	0.115
SPBC20F10.07	-0.09	-0.042	-0.004
SPBC20F10.10	0.029	0.035	0.029
SPBC21.03c	0.031	0.114	0.172
SPBC21.07c	0.053	0.039	0.078
SPBC211.06	-0.003	-0.01	0.122
SPBC215.01	-0.003	-0.102	-0.037
SPBC215.02	-0.21	0.024	0.014
SPBC215.03c	0.049	0.25	NA
SPBC215.04	0.02	0.116	0.24
SPBC215.05	NA	NA	0.311
SPBC215.06c	-0.034	0.024	-0.015
SPBC215.07c	-0.02	-0.036	0.042
SPBC215.08c	0.066	-0.58	-0.644
SPBC215.10	0.005	0.017	0.152
SPBC215.11c	-0.007	0.006	0.057
SPBC215.13	0.008	0.007	0.211
SPBC215.14c	0.002	-0.13	-0.768
SPBC216.01c	-0.053	-0.001	-0.088
SPBC216.02	0.049	0.039	0.063
SPBC216.03	-0.119	-0.162	-0.226
SPBC216.04c	0.115	0.105	0.099
SPBC216.05	-0.068	-0.121	-0.007
SPBC216.06c	-0.043	0.135	0.193
SPBC21B10.02	0.088	0.089	-0.299
SPBC21B10.04c	0	-0.048	-0.155
SPBC21B10.05c	0.046	0	0.138
SPBC21B10.06c	-0.01	0.113	0.022
SPBC21B10.07	-0.006	-0.003	0.159
SPBC21B10.08c	0.036	-0.04	-0.01
SPBC21B10.09	0.054	0.022	0.182
SPBC21B10.10	-0.13	-0.132	-0.447
SPBC21B10.12	0.045	0.09	0.096
SPBC21B10.13c	-0.042	-0.044	-0.263
SPBC21C3.01c	0.016	0.005	-0.086
SPBC21C3.03	0.08	0.022	-0.019
SPBC21C3.06	0.054	0.069	0.034
SPBC21C3.07c	-0.578	-1.743	-0.485
SPBC21C3.08c	0.165	0.077	0.067
SPBC21C3.09c	-0.007	-0.011	-0.098
SPBC21C3.11	-0.085	-0.327	-1.066
SPBC21C3.12c	0.027	0.069	0.145
SPBC21C3.14c	-0.037	-0.315	0.001
SPBC21C3.15c	0.007	0.041	0.122
SPBC21C3.17c	-0.047	0.085	0.022
SPBC21C3.19	-0.02	0.077	-0.367

SPBC21C3.20c	-0.072	-0.388	0.137
SPBC21D10.07	0.034	0.058	0.179
SPBC21D10.08c	0.113	0.108	0.182
SPBC21D10.09c	-0.011	-0.136	0.075
SPBC21D10.11c	0.053	0.006	0.052
SPBC21D10.12	0.02	-0.016	0.088
SPBC21H7.03c	0.032	0.121	0.074
SPBC21H7.06c	-0.011	0.016	-0.001
SPBC21H7.07c	0.544	-0.341	-0.264
SPBC23E6.01c	0.017	0.061	0.173
SPBC23E6.02	-0.019	-0.02	0.046
SPBC23E6.03c	0.056	0.052	0.054
SPBC23E6.05	-0.05	-0.027	0.052
SPBC23E6.10c	-0.048	-0.006	0.137
SPBC23G7.04c	-0.045	-0.082	-0.021
SPBC23G7.06c	-0.031	-0.395	0.121
SPBC23G7.07c	0.03	0.076	0.143
SPBC23G7.08c	0.564	0.681	0.379
SPBC23G7.10c	-0.004	0.032	0.057
SPBC23G7.11	-0.007	-0.016	0.024
SPBC23G7.12c	-0.031	-0.122	-0.192
SPBC23G7.13c	-0.017	-0.051	-0.046
SPBC23G7.14	0.054	0.109	0.236
SPBC23G7.15c	0.058	-0.093	0.08
SPBC23G7.16	0.704	NA	NA
SPBC24C6.04	0.047	-0.05	-0.324
SPBC24C6.08c	0.056	-0.125	-0.015
SPBC24C6.09c	0.052	0.068	-0.058
SPBC25B2.02c	-0.014	0.075	0.094
SPBC25B2.03	0.05	-0.018	-0.815
SPBC25B2.04c	0.143	-0.207	-0.343
SPBC25B2.06c	0.099	-0.004	0.28
SPBC25B2.07c	0.042	0.033	0.014
SPBC25B2.08	-0.009	-0.229	-0.123
SPBC25B2.10	-0.02	0.117	-0.116
SPBC25B2.11	0.007	0.059	0.27
SPBC25D12.02c	0.047	0.078	-0.312
SPBC25D12.05	0.013	0.015	-0.181
SPBC25H2.03	0.028	0.008	0.046
SPBC25H2.05	-0.129	-0.067	-0.223
SPBC25H2.08c	0.227	-0.156	-0.697
SPBC25H2.09	0.047	0.066	-0.185
SPBC25H2.10c	0.049	-0.105	-0.448
SPBC25H2.14	0.087	0.059	0.104
SPBC25H2.16c	0.072	-0.253	-0.572
SPBC26H8.01	0.098	0.041	-0.63
SPBC26H8.05c	-0.089	-0.06	0.023
SPBC26H8.09c	-0.037	0.014	-0.008
SPBC26H8.11c	-0.016	0.07	0.065
SPBC27.02c	-0.117	0.077	-0.019
SPBC27.03	0.054	0.057	0.148
SPBC27.04	0.027	0.052	0.107
SPBC27.08c	-0.009	0.064	-0.807
SPBC27B12.03c	-0.028	-0.098	0.079
SPBC27B12.05	-0.04	0.026	0.006
SPBC27B12.07	0.077	0.072	0.076
SPBC27B12.08	-0.125	-1.337	-1.552
SPBC27B12.10c	0.071	-0.808	0.042
SPBC27B12.11c	0.177	0.115	-0.134
SPBC27B12.14	-0.031	-0.031	0.059
SPBC28E12.02	0.011	0.024	0.017
SPBC28E12.03	-0.079	-0.191	-0.204
SPBC28E12.04	0.014	0.046	0.13
SPBC28E12.06c	0.034	0.118	0.305
SPBC28F2.02	0.124	0.119	0.289
SPBC28F2.03	-0.044	-0.017	0.013
SPBC28F2.05c	0.012	-0.016	0.206
SPBC28F2.08c	0.024	-0.11	0.089
SPBC28F2.10c	0.003	0.103	0
SPBC29A10.01	0.037	0.193	-0.771
SPBC29A10.02	-0.14	-0.309	-0.93
SPBC29A10.03c	0.023	-0.017	-0.024
SPBC29A10.05	-0.005	0.036	0.195

SPBC29A10.06c	-0.002	-0.018	-0.022
SPBC29A10.07	-0.069	0.02	0.024
SPBC29A10.08	0.118	0.309	0.316
SPBC29A10.09c	-0.054	0.051	0.061
SPBC29A10.11c	0.056	0.006	0.225
SPBC29A10.12	0.028	-0.1	0.117
SPBC29A10.14	-0.094	-0.029	0.02
SPBC29A3.01	0.432	0.598	-0.433
SPBC29A3.02c	0.01	-0.02	0.108
SPBC29A3.03c	-0.052	-0.172	-0.268
SPBC29A3.05	0.163	0.14	-0.141
SPBC29A3.07c	-0.132	-0.145	-0.305
SPBC29A3.08	0	0.018	-0.176
SPBC29A3.11c	0.035	0.113	0.2
SPBC29A3.12	0.056	0.016	0.103
SPBC29A3.13	-0.065	-0.051	0.091
SPBC29A3.14c	-0.037	-0.003	0.169
SPBC29A3.17	-0.034	0.114	0.257
SPBC29A3.18	0.011	-0.029	-0.032
SPBC29B5.02c	0	-0.017	-0.058
SPBC29B5.03c	-0.393	-0.262	-1.153
SPBC29B5.04c	-0.023	-0.038	0.119
SPBC2A9.02	-0.02	-0.062	-0.345
SPBC2A9.03	-0.003	-0.025	-0.181
SPBC2A9.04c	0.051	-0.008	-0.218
SPBC2A9.05c	0.039	-0.011	0.079
SPBC2A9.07c	-0.022	-0.035	0.195
SPBC2A9.11c	0.042	0.044	0.128
SPBC2A9.13	-0.061	-0.161	0.037
SPBC2D10.03c	-0.006	0.018	0.06
SPBC2D10.04	0.022	-0.02	0.208
SPBC2D10.05	-0.004	0.073	-0.434
SPBC2D10.06	-0.052	-0.049	0.048
SPBC2D10.09	0.038	0.047	0.092
SPBC2D10.11c	-0.014	0.082	0.144
SPBC2D10.12	-0.564	-0.39	-0.143
SPBC2D10.13	0.317	0.395	0.403
SPBC2D10.14c	0.038	0.052	-0.074
SPBC2D10.15c	0.081	-0.001	-0.029
SPBC2D10.16	-1.056	0.031	-1.04
SPBC2D10.17	-0.096	-0.519	-0.729
SPBC2D10.19c	-0.053	0.048	0.109
SPBC2D10.20	0.073	-0.05	0.149
SPBC2F12.03c	0.014	-0.046	0.01
SPBC2F12.04	-0.132	-0.226	-0.53
SPBC2F12.05c	-0.012	-0.07	-0.225
SPBC2F12.09c	0.474	0.228	0.537
SPBC2F12.13	0.008	0.016	0.154
SPBC2F12.15c	-0.128	-0.072	0.054
SPBC2G2.01c	0.215	0.051	-1.082
SPBC2G2.02	0.035	-0.006	-0.462
SPBC2G2.03c	-0.19	0.149	-1.797
SPBC2G2.05	0.027	0.009	-0.091
SPBC2G2.06c	-0.042	-0.074	0.012
SPBC2G2.08	-0.006	0.031	0.08
SPBC2G2.09c	0.021	0.04	0.08
SPBC2G2.10c	-0.428	-0.121	-1.533
SPBC2G2.14	-0.044	0.027	0.113
SPBC2G2.15c	-0.082	0.065	0.043
SPBC2G2.17c	-0.148	0.023	-0.858
SPBC2G5.01	-0.049	-0.075	-0.16
SPBC2G5.02c	-0.188	0.03	0.083
SPBC2G5.03	-0.012	-0.122	0.133
SPBC2G5.04c	0.04	0.023	0.201
SPBC2G5.06c	0.385	NA	NA
SPBC30B4.01c	0.572	-0.128	-0.339
SPBC30B4.02c	0.017	-0.034	-0.093
SPBC30B4.03c	-0.211	-0.063	-0.292
SPBC30B4.04c	-0.043	-0.54	-0.008
SPBC30B4.06c	-0.093	-0.11	-1.28
SPBC30B4.08	-0.024	-0.113	-0.075
SPBC30D10.03c	0.044	0.061	0.128
SPBC30D10.04	-0.036	0.004	0.13

SPBC30D10.05c	0.008	-0.123	-0.065
SPBC30D10.09c	0.123	-0.271	0.25
SPBC30D10.10c	-0.27	0.155	-0.135
SPBC30D10.13c	-0.386	-0.03	-0.54
SPBC30D10.14	-0.007	-0.041	-0.001
SPBC30D10.16	0.271	0.284	0.387
SPBC317.01	0.011	-0.128	-0.083
SPBC31A8.01c	-0.001	-0.019	0.022
SPBC31E1.01c	0.091	0.031	0.288
SPBC31E1.02c	-0.262	-0.17	-0.852
SPBC31F10.02	0.033	-0.037	0.024
SPBC31F10.03	-0.029	-0.032	0.118
SPBC31F10.05	0.004	0.069	0.118
SPBC31F10.07	0.487	0.397	0.062
SPBC31F10.08	0.034	0.047	-0.155
SPBC31F10.12	0.032	0.158	-0.416
SPBC31F10.13c	-0.032	-0.023	-0.303
SPBC31F10.14c	-0.182	0.086	-0.17
SPBC31F10.15c	-0.016	-0.007	-0.528
SPBC31F10.16	-0.031	-0.025	-0.201
SPBC31F10.17c	-0.018	0.029	0.115
SPBC32C12.03c	-0.027	-0.034	0.112
SPBC32F12.01c	0.371	-1.462	-1.095
SPBC32F12.02	-0.121	-0.201	-0.191
SPBC32F12.03c	0.041	0.036	-0.125
SPBC32F12.07c	-0.032	-0.071	0.017
SPBC32F12.09	0.01	0.103	0.154
SPBC32F12.11	0.137	0.201	-0.351
SPBC32F12.12c	-0.104	0.004	-0.01
SPBC32H8.01c	-0.12	0.002	0.107
SPBC32H8.02c	-0.082	-0.129	-1.102
SPBC32H8.03	-0.022	-0.119	0.067
SPBC32H8.05	-0.059	0.017	0.124
SPBC32H8.06	0.021	-0.04	0.061
SPBC32H8.07	0.101	0.352	0.356
SPBC32H8.08c	-0.005	0.016	0.036
SPBC32H8.11	-0.016	0.042	-0.017
SPBC32H8.13c	-0.135	0	0.035
SPBC336.01	0.071	0.237	-0.053
SPBC336.03	-0.061	-0.19	-1.017
SPBC336.05c	-0.029	-0.053	0.128
SPBC336.06c	0.007	0.12	0.304
SPBC336.10c	-0.034	0.015	-0.175
SPBC336.13c	0.118	-0.057	-0.404
SPBC336.14c	-0.205	-0.066	-0.185
SPBC337.03	-0.02	-0.097	-0.023
SPBC337.04	-0.032	-0.083	-0.046
SPBC337.07c	-0.102	-0.063	-0.001
SPBC337.10c	-0.045	0.014	0.131
SPBC337.11	-0.031	0.029	-0.046
SPBC337.16	0.074	-1.42	-2
SPBC342.03	0.006	-0.017	0.111
SPBC342.05	-0.023	-0.041	0.088
SPBC342.06c	-0.019	-0.025	0.004
SPBC354.01	0.019	0.073	0.034
SPBC354.03	-0.179	0.045	-0.305
SPBC354.04	0.093	0.171	0.257
SPBC354.05c	-0.07	-0.108	-0.442
SPBC354.07c	0.034	0.013	0.032
SPBC354.08c	0.003	-0.015	0.122
SPBC354.09c	0.031	0.046	0.309
SPBC354.10	-0.267	-1.972	-1.249
SPBC354.12	-0.01	-0.011	-0.736
SPBC354.13	-0.01	-0.002	-0.005
SPBC354.14c	0.066	-0.405	-0.196
SPBC354.15	-0.028	0.075	0.067
SPBC359.01	-0.013	-0.072	0.086
SPBC359.03c	-0.001	0.013	0.103
SPBC359.04c	0.045	-0.008	-0.188
SPBC359.05	-0.015	-0.017	-0.63
SPBC359.06	-0.005	0.006	0.06
SPBC36.01c	-0.042	0.012	0.068
SPBC36.03c	-0.086	-0.017	0.187

SPBC36.04	-1.411	-2	-2
SPBC36.06c	-0.245	0.055	-0.133
SPBC36.10	0.09	0.016	0.046
SPBC36.11	-0.117	-0.07	0.051
SPBC365.01	0.002	-0.017	0.11
SPBC365.07c	0.071	0	0.125
SPBC365.11	-0.06	-0.025	-0.011
SPBC365.12c	-0.009	-0.243	-0.021
SPBC365.13c	0.103	0.026	-0.104
SPBC365.16	-0.111	-2	-0.589
SPBC365.20c	0.026	-0.033	0.109
SPBC36B7.02	-0.212	0.033	0.155
SPBC36B7.03	-0.027	-0.288	0.053
SPBC36B7.04	0.013	0.041	-0.28
SPBC36B7.05c	-0.048	-0.045	0.092
SPBC36B7.06c	-0.072	-0.067	0.08
SPBC36B7.08c	-0.053	0.007	0.046
SPBC3B8.02	0.181	0.175	-0.566
SPBC3B8.03	-0.065	-0.329	-0.581
SPBC3B8.04c	-0.104	-0.012	-0.046
SPBC3B8.05	0.102	0.02	-0.329
SPBC3B8.06	0.003	-0.042	0.003
SPBC3B8.07c	-0.035	0.038	-1.201
SPBC3B8.08	0.007	-0.031	-0.116
SPBC3B8.10c	0.018	-1.139	-1.071
SPBC3B9.04	0.04	0.024	0.199
SPBC3B9.05	0.006	0.059	0.218
SPBC3B9.06c	0.21	0.17	0.356
SPBC3B9.08c	-0.012	0.015	-0.035
SPBC3B9.09	-0.036	0.07	-1.471
SPBC3B9.13c	0.177	0.394	-0.296
SPBC3B9.15c	0.013	-0.067	-0.029
SPBC3D6.02	0.027	-0.052	-0.167
SPBC3D6.04c	-0.028	-0.052	-0.524
SPBC3D6.06c	0.021	-0.039	0.137
SPBC3D6.09	0.311	0.021	0.663
SPBC3D6.10	-0.003	0.033	0.169
SPBC3D6.13c	-0.002	-0.042	0.112
SPBC3E7.02c	0.03	0.023	0.121
SPBC3E7.05c	0.172	-0.048	-0.516
SPBC3E7.06c	-0.024	-0.047	-0.184
SPBC3E7.07c	-0.022	-0.078	-0.072
SPBC3E7.08c	-0.042	-0.099	-0.112
SPBC3E7.09	0.04	-0.014	0.065
SPBC3E7.11c	0.036	-0.055	0.114
SPBC3E7.12c	-0.005	-0.066	0.072
SPBC3E7.15c	-0.103	-0.101	0.063
SPBC3E7.16c	0.048	0	-0.073
SPBC3F6.01c	0.026	-0.047	-0.493
SPBC3F6.05	0.002	0.116	0.122
SPBC3H7.03c	0.031	-0.6	-0.841
SPBC3H7.05c	-0.054	0.055	-0.134
SPBC3H7.06c	-0.014	0.034	0.1
SPBC3H7.07c	0.006	0.102	0.249
SPBC3H7.09	0.324	-1.054	-1.074
SPBC3H7.10	0.16	-1.058	-0.821
SPBC3H7.11	0.047	0.024	0.171
SPBC3H7.12	-0.215	-0.273	-1.05
SPBC3H7.13	-0.038	-0.008	-0.54
SPBC3H7.14	-0.057	-0.046	-0.059
SPBC3H7.15	0.718	0.917	0.17
SPBC4.02c	0.194	0.06	0.05
SPBC4.05	-0.028	-0.036	0.079
SPBC4.06	-0.033	0.017	-0.217
SPBC405.02c	-0.033	-0.085	-0.15
SPBC405.03c	0.009	0.023	-0.135
SPBC405.04c	-1.131	-1.768	-2
SPBC405.05	0.023	0.064	0.146
SPBC405.06	-0.013	-0.031	-0.061
SPBC409.03	-0.052	-0.034	0.137
SPBC409.08	0	-0.013	0.116
SPBC409.10	-0.05	-0.025	-0.001
SPBC409.11	0.023	0.058	0.062

SPBC409.16c	-0.045	-0.087	0.095
SPBC409.17c	-0.031	-0.005	0.092
SPBC409.19c	0.265	-1.201	-0.817
SPBC418.01c	0.051	0.018	0.122
SPBC418.02	-0.081	-0.078	0.089
SPBC428.03c	-0.032	-0.197	-0.365
SPBC428.04	0.057	-0.013	0.159
SPBC428.05c	-0.128	-1.215	-0.624
SPBC428.07	0.029	0.081	0.066
SPBC428.08c	-0.336	-0.405	-1.322
SPBC428.10	0.018	-0.046	-0.093
SPBC428.11	-0.009	0.045	0.106
SPBC428.12c	0.132	-0.003	0.171
SPBC428.14	0.026	-0.118	0.187
SPBC428.15	0.014	0.005	0.033
SPBC428.17c	-0.013	-0.051	0.155
SPBC4B4.02c	0.057	0.101	0.126
SPBC4B4.03	0.017	0.039	0.025
SPBC4B4.04	0.02	-0.066	0.031
SPBC4B4.07c	0.011	0.019	0.105
SPBC4B4.10c	-0.015	-0.044	0.006
SPBC4B4.12c	0.008	-0.074	-0.638
SPBC4C3.04c	-0.031	-0.012	0.016
SPBC4C3.06	0.068	0.018	0.244
SPBC4C3.08	0.091	0.068	-0.007
SPBC4C3.09	0.035	-0.021	0.023
SPBC4C3.12	-0.224	0.049	-1.53
SPBC4F6.05c	-0.174	-0.202	-0.347
SPBC4F6.08c	-0.034	-0.336	-0.564
SPBC4F6.09	0.037	0.076	0.119
SPBC4F6.11c	-0.076	-0.071	0.006
SPBC4F6.12	-0.071	-0.049	-0.262
SPBC4F6.15c	0.011	0.071	0.161
SPBC4F6.16c	-0.033	0.01	-0.002
SPBC530.01	0.061	0.044	-0.324
SPBC530.03c	-0.042	0.082	0.085
SPBC530.04	-0.017	0.024	0.09
SPBC530.06c	-0.026	-0.047	-0.284
SPBC530.07c	0.028	0.026	-0.172
SPBC530.08	0.021	0.055	0.233
SPBC530.09c	-0.039	-0.033	-0.071
SPBC530.11c	-0.072	-0.266	-0.67
SPBC530.13	-0.101	-0.055	-0.431
SPBC530.14c	0.112	0.089	-0.503
SPBC530.15c	0.021	0.023	0.006
SPBC543.02c	-0.003	0.033	0.103
SPBC543.03c	0.022	0.038	-0.247
SPBC543.05c	-0.048	0.034	-0.07
SPBC543.07	-0.046	-0.074	-0.246
SPBC543.08	0.01	-0.074	0.003
SPBC543.10	0.025	0.011	-0.224
SPBC557.04	0.021	-0.029	-0.086
SPBC557.05	-0.082	0.053	-0.051
SPBC56F2.03	-0.023	-0.058	0.055
SPBC56F2.04	-0.058	0.025	0.233
SPBC56F2.05c	-0.129	-0.077	-1.022
SPBC56F2.06	0.105	0.042	0.049
SPBC56F2.08c	0.097	0.231	-0.237
SPBC56F2.09c	0.077	0.055	-0.364
SPBC56F2.10c	0.043	-0.046	-0.05
SPBC56F2.14	-0.038	-0.047	0.056
SPBC577.02	-0.036	0.321	-0.166
SPBC577.03c	-0.009	-0.038	-0.104
SPBC577.04	0.028	0.106	0.173
SPBC577.05c	-0.098	-0.169	-0.002
SPBC577.06c	-0.049	-0.095	0.029
SPBC577.08c	-0.042	0.104	0.131
SPBC577.11	-0.03	-0.003	-0.384
SPBC577.12	-0.035	-0.015	-0.692
SPBC577.13	0.027	0.01	-0.024
SPBC577.14c	-0.007	-0.071	-0.818
SPBC582.05c	0.025	-0.001	-0.228
SPBC582.06c	-0.04	-0.147	-0.061

SPBC582.08	-0.079	-0.074	-0.033
SPBC582.09	0.004	-0.025	0.05
SPBC582.10c	0.082	0.035	0.097
SPBC609.02	0.316	NA	-0.208
SPBC609.03	-0.071	-0.018	0.029
SPBC609.04	0.016	-0.005	0.026
SPBC609.05	-0.041	-0.01	-0.081
SPBC646.02	-0.034	-0.002	0.102
SPBC646.06c	-0.117	-0.089	-0.218
SPBC646.08c	-0.061	-0.153	-0.519
SPBC646.09c	0.196	0.704	0.846
SPBC649.02	-0.038	0	-0.103
SPBC649.03	0.159	0.008	0.209
SPBC649.04	0.024	0.077	0.111
SPBC651.02	NA	-0.257	NA
SPBC651.03c	-1.225	-0.066	-0.917
SPBC651.04	0.07	-0.058	0.113
SPBC651.05c	-0.002	0.029	-0.007
SPBC651.06	0.039	-0.092	0.21
SPBC651.11c	0.119	0.217	0.206
SPBC651.12c	0.02	0.082	0.065
SPBC660.05	0.028	-0.006	-0.076
SPBC660.06	-0.046	0.006	-0.098
SPBC660.07	0.206	0.05	0.17
SPBC660.09	-0.023	-0.098	0.043
SPBC660.10	-0.157	0.017	-0.279
SPBC660.11	0.015	0.135	-0.108
SPBC660.12c	0.004	0.012	0.035
SPBC660.14	0.032	0.036	0.081
SPBC660.17c	0.004	0.037	-0.277
SPBC685.02	-0.071	-0.02	-0.175
SPBC685.03	0.007	-0.004	-0.027
SPBC685.04c	0.03	-0.053	0.168
SPBC685.06	-0.115	-0.298	-0.542
SPBC691.01	0.003	-0.007	-0.055
SPBC691.03c	0.034	0.004	0.076
SPBC691.04	NA	0.152	0.275
SPBC691.05c	-0.035	-0.046	0.176
SPBC6B1.02	-0.035	-0.061	-0.005
SPBC6B1.03c	-0.024	-0.003	-0.041
SPBC6B1.04	-0.098	-0.361	-0.263
SPBC6B1.05c	0.044	-0.004	0.075
SPBC6B1.06c	0.048	-0.017	-0.729
SPBC6B1.08c	-0.006	-0.057	-0.137
SPBC6B1.09c	0	0.126	0.193
SPBC6B1.10	-0.091	0.067	0.051
SPBC713.03	-0.085	0.024	-0.206
SPBC713.05	0.001	0.038	0.073
SPBC713.06	-0.068	-0.025	0.042
SPBC713.07c	0.017	0.024	0.16
SPBC713.09	0.008	0.052	-0.177
SPBC713.11c	-0.018	-0.013	-0.024
SPBC725.01	0.166	0.151	-0.623
SPBC725.02	-0.172	-0.508	-0.641
SPBC725.03	0.127	0.126	0.084
SPBC725.04	0.048	0.104	0.142
SPBC725.05c	-0.046	-0.029	0.089
SPBC725.06c	0.376	0.127	0.27
SPBC725.07	-0.022	-0.16	-1.09
SPBC725.10	-0.071	-0.328	-0.289
SPBC725.11c	-0.041	0.004	-0.015
SPBC725.12	-0.013	-0.137	-0.173
SPBC725.14	0.016	0.056	-0.63
SPBC725.15	-0.029	0.083	-0.073
SPBC776.01	-0.003	0.061	-0.063
SPBC776.02c	-0.064	-0.106	-0.455
SPBC776.03	0.133	-0.092	-0.193
SPBC776.04	-0.053	0.03	-0.024
SPBC776.05	0.357	0.121	0.409
SPBC776.06c	0.112	0.145	-0.2
SPBC776.11	-0.016	-0.027	0.123
SPBC776.14	-0.027	0.017	0.148
SPBC776.15c	0.196	-0.035	-0.485

SPBC776.16	-0.082	0.016	-0.126
SPBC800.02	-0.08	-0.092	-0.131
SPBC800.03	0.356	0.108	-0.122
SPBC800.04c	-0.036	0.059	-0.162
SPBC800.05c	-0.058	-0.051	-0.794
SPBC800.08	0.117	0.029	0.007
SPBC800.11	-0.003	-0.007	0.105
SPBC800.12c	-0.035	0.025	0.06
SPBC83.01	0.112	-0.004	-0.303
SPBC83.02c	0.022	0.055	-0.738
SPBC83.03c	-0.018	-0.472	-0.318
SPBC83.04	0.018	0.056	0.157
SPBC83.05	-0.002	0.048	0.107
SPBC83.09c	0.114	-0.088	-0.596
SPBC83.10	0.002	-0.223	-0.096
SPBC83.11	0.045	0.006	-0.055
SPBC83.13	0.004	-0.002	0.219
SPBC83.16c	-0.141	-0.003	-0.154
SPBC83.17	0.015	-0.061	0.067
SPBC83.18c	-0.139	0.031	-0.072
SPBC83.19c	0.024	0.023	0.22
SPBC839.02	0.016	-0.016	0.076
SPBC839.03c	-0.075	0.009	0.033
SPBC839.04	0.034	0.013	-0.174
SPBC839.05c	0.045	0.104	-0.285
SPBC839.06	0.034	-0.016	0.173
SPBC839.07	0.013	0.003	0.031
SPBC839.11c	-0.014	0.037	0.007
SPBC839.13c	-0.043	-0.009	-0.188
SPBC839.14c	-0.022	-0.01	-0.182
SPBC839.15c	0.035	-0.098	-0.055
SPBC839.17c	0.023	0.08	0.136
SPBC887.01	0.019	0.045	0.152
SPBC887.02	0.067	0.153	0.182
SPBC887.04c	0.011	-0.091	0.155
SPBC887.05c	-0.164	0.272	0.523
SPBC887.06c	0.024	0	-0.086
SPBC887.08	0.043	0.103	-0.091
SPBC887.11	-0.019	-0.228	0.178
SPBC887.17	0.107	0.051	0.31
SPBC8D2.01	-0.254	-0.045	-0.826
SPBC8D2.02c	-0.155	-0.108	-1.214
SPBC8D2.03c	0.006	0.029	-0.069
SPBC8D2.04	0.022	0.02	0.268
SPBC8D2.10c	-0.111	-0.03	-0.053
SPBC8D2.11	0.014	0.029	-0.219
SPBC8D2.12c	-0.157	-0.012	0.111
SPBC8D2.16c	0.032	0.049	0.124
SPBC8D2.17	-0.449	0.424	0.179
SPBC8D2.18c	-0.113	-0.342	-0.587
SPBC8D2.19	0.037	0.034	0.004
SPBC8E4.01c	0.059	-0.104	0.294
SPBC8E4.02c	-0.012	-0.024	0.039
SPBC8E4.03	-0.013	-0.022	0.169
SPBC8E4.04	-0.028	0.004	0.014
SPBC8E4.05c	-0.011	-0.04	0.172
SPBC902.02c	-0.041	-0.179	-0.279
SPBC902.03	0.08	-0.546	-0.768
SPBC902.04	-0.072	0.064	0.014
SPBC902.05c	0.029	0.038	0.148
SPBC902.06	0.035	0.054	0.109
SPBC947.01	-0.016	-0.018	0.095
SPBC947.03c	-0.083	-0.113	-0.665
SPBC947.04	0.028	0.001	-0.133
SPBC947.05c	-0.02	0.01	0.188
SPBC947.06c	-0.029	-0.07	-0.048
SPBC947.09	0.011	-0.054	0.239
SPBC947.10	-0.053	0.015	0.038
SPBC947.11c	0.044	0.07	0.216
SPBC947.15c	-0.117	-0.031	-1.138
SPBC9B6.03	-0.044	-0.042	-0.275
SPBC9B6.09c	-0.016	-0.022	-0.004
SPBC9B6.11c	-0.007	-0.025	0.094

SPBCPT2R1.01c	-0.032	-0.047	-0.189
SPBCPT2R1.02	-0.005	-0.068	0.156
SPBCPT2R1.08c	0.062	-0.358	-0.482
SPBP16F5.03c	-0.014	0.02	-0.8
SPBP16F5.04	0.017	-0.037	0.173
SPBP16F5.05c	-0.105	-0.108	-0.132
SPBP16F5.08c	-0.044	0.042	0.131
SPBP18G5.03	-0.006	-0.051	0.001
SPBP19A11.02c	-0.047	0.004	0.035
SPBP22H7.04	-0.007	0.006	0.15
SPBP22H7.05c	0.016	0.025	0.115
SPBP22H7.06	-0.015	-0.007	0.118
SPBP23A10.05	-0.069	0.104	0.286
SPBP23A10.10	-0.076	-0.014	0.022
SPBP23A10.12	0.054	0.142	0.236
SPBP23A10.14c	-0.013	0.073	-1.345
SPBP26C9.02c	-0.029	-0.371	0.132
SPBP35G2.02	0.03	-0.04	0.121
SPBP35G2.03c	0.027	-0.015	-0.01
SPBP35G2.04c	0.081	0.098	0.192
SPBP35G2.05c	0.023	-0.087	0.186
SPBP35G2.06c	-0.043	0.013	0.096
SPBP35G2.07	-0.154	-0.923	-0.776
SPBP35G2.08c	0.091	0.312	0.241
SPBP35G2.10	-0.035	-0.012	-0.313
SPBP35G2.11c	0.075	0.098	0.152
SPBP35G2.12	0.033	0.028	0.21
SPBP35G2.13c	-0.017	0.012	-0.068
SPBP4G3.02	-0.039	-0.086	-0.028
SPBP4G3.03	-0.001	-0.064	0.136
SPBP4H10.03	-0.124	0	-0.476
SPBP4H10.05c	-0.024	-0.016	-0.123
SPBP4H10.07	0.001	0.08	0.095
SPBP4H10.08	0.04	0.031	0.095
SPBP4H10.09	-0.073	0.087	-0.028
SPBP4H10.10	-0.031	-0.047	0.133
SPBP4H10.12	-0.03	-0.028	0.133
SPBP4H10.13	-0.243	-0.122	-0.939
SPBP4H10.14c	0.033	0.027	0.147
SPBP4H10.16c	-0.016	-0.018	-0.115
SPBP4H10.17c	-0.103	-0.018	-0.393
SPBP4H10.18c	0.043	0.033	0.089
SPBP4H10.19c	0.036	0.011	-0.068
SPBP4H10.20	-0.121	-0.066	-0.198
SPBP8B7.02	-0.01	0.071	0.057
SPBP8B7.04	-0.044	-0.07	0.033
SPBP8B7.06	-0.046	-0.099	-0.263
SPBP8B7.07c	0.041	0.014	0.079
SPBP8B7.08c	-0.032	0.017	0.052
SPBP8B7.09c	0.063	-0.067	-0.049
SPBP8B7.10c	-0.035	0.026	-0.042
SPBP8B7.11	-0.057	-0.735	-0.457
SPBP8B7.13	-0.029	0.037	-0.044
SPBP8B7.18c	-0.056	-0.456	-0.519
SPBP8B7.21	0.021	0.101	0.087
SPBP8B7.23	-0.004	-0.004	-0.033
SPBP8B7.24c	0.228	0.266	0.35
SPBP8B7.25	0.048	-0.003	0.063
SPBP8B7.26	0.167	0.133	0.272
SPBP8B7.27	0.029	0.084	0.238
SPBP8B7.30c	0.078	-0.013	-0.019
SPBP8B7.31	0.088	0.144	0.188
SPBPB10D8.01	-0.011	-0.066	-0.006
SPBPB10D8.02c	0.03	-0.011	0.115
SPBPB10D8.04c	0.065	0.042	0.155
SPBPB10D8.05c	-0.037	-0.016	0.211
SPBPB10D8.06c	-0.015	-0.187	0.075
SPBPB21E7.01c	-0.068	-0.017	0.032
SPBPB21E7.04c	0.077	0.258	0.209
SPBPB21E7.05	0.115	-0.15	0.05
SPBPB21E7.09	0.069	0.075	0.207
SPBPB2B2.01	-0.055	-0.047	0.087
SPBPB2B2.02	0.045	-0.258	-0.151

SPBPB2B2.05	0.006	0.001	-0.028
SPBPB2B2.07c	0.019	0.05	-0.102
SPBPB2B2.08	0.05	0.002	-0.11
SPBPB2B2.09c	0.559	0.527	0.531
SPBPB2B2.10c	-0.082	-0.057	-0.241
SPBPB2B2.11	-0.03	-0.041	-0.045
SPBPB2B2.12c	-0.024	0.009	-0.123
SPBPB2B2.13	0.053	0.013	0.031
SPBPB2B2.14c	0.032	0.036	0.055
SPBPB7E8.01	0.053	-0.093	0.14
SPBPB7E8.02	0.075	-0.025	-0.587
SPBPB8B6.04c	-0.016	-0.055	0.101
SPBPB8B6.05c	0.033	-0.031	0.073
SPBPJ4664.02	-0.086	-0.114	0.108
SPBPJ4664.03	-0.043	-0.026	-0.074
SPBPJ4664.05	-0.036	-0.044	0.138
SPBPJ4664.06	-0.107	-0.15	-0.103
SPCC1020.01c	0.55	-0.483	0.424
SPCC1020.03	0.1	0.201	0.344
SPCC1020.05	0.085	0.054	-0.065
SPCC1020.06c	0.025	-0.003	-0.328
SPCC1020.07	-0.037	0.204	0.266
SPCC1020.08	0.145	0.091	-0.418
SPCC1020.09	0.106	0.082	0.136
SPCC1020.10	0.152	0.028	0.277
SPCC1020.11c	0.273	-0.2	0.141
SPCC1020.12c	0.145	0.115	0.045
SPCC1020.13c	0.096	0.135	0.177
SPCC1183.02	0.125	-0.078	0.012
SPCC1183.06	0.052	0.058	0.16
SPCC1183.09c	-0.104	-0.619	-0.698
SPCC1183.10	0.111	0.045	0.265
SPCC1183.11	-0.063	-0.072	0.027
SPCC1223.01	0.022	0.047	-0.086
SPCC1223.02	0.059	0.001	-0.15
SPCC1223.03c	0.008	0.004	0.044
SPCC1223.04c	0.135	-0.121	-0.032
SPCC1223.06	0.075	0.035	-0.345
SPCC1223.10c	0.112	-0.119	-0.542
SPCC1223.11	-0.207	-0.195	-0.906
SPCC1223.12c	0.064	-0.058	-0.263
SPCC1223.13	0.107	0.02	-0.002
SPCC1223.15c	0.169	0.136	0.284
SPCC1235.01	-0.059	-0.039	-0.51
SPCC1235.02	-0.041	-0.095	-0.344
SPCC1235.03	0.001	-0.243	-0.346
SPCC1235.05c	0.125	-0.056	-0.043
SPCC1235.06	0.048	0.177	0.281
SPCC1235.08c	-0.033	-0.174	-0.751
SPCC1235.11	0.181	0.362	-0.246
SPCC1235.12c	-0.05	-0.013	-0.418
SPCC1235.13	0.123	0.025	-0.198
SPCC1235.15	-0.02	-0.082	-0.027
SPCC1259.02c	0.679	0.556	0.568
SPCC1259.04	0.509	0.369	0.226
SPCC1259.05c	0.049	0.076	0.26
SPCC1259.08	0.336	0.339	0.168
SPCC1259.09c	0.24	0.24	0.028
SPCC1259.10	0.952	0.841	0.85
SPCC1259.11c	0.249	0.1	0.219
SPCC1259.13	0.287	0.36	0.364
SPCC1259.14c	0.275	0.309	0.328
SPCC1393.02c	0.091	0.216	0.067
SPCC1393.05	0.053	0.065	-0.295
SPCC1393.08	NA	0.277	0.475
SPCC1393.09c	0.112	0.051	0.017
SPCC13B11.01	-0.376	0.028	-0.361
SPCC13B11.02c	0.097	0.036	-0.238
SPCC13B11.03c	0.054	0.049	-0.467
SPCC13B11.04c	0.13	0.066	0.116
SPCC1442.02	-0.039	-0.753	-0.994
SPCC1442.04c	0.16	-0.144	-0.489
SPCC1442.05c	-0.035	-0.143	0.017

SPCC1442.07c	0.024	-0.055	-0.107
SPCC1442.11c	0.05	-0.078	0.071
SPCC1442.13c	0.024	-0.133	-0.054
SPCC1442.14c	0.016	-0.056	-0.099
SPCC1442.15c	-0.038	0.212	-0.227
SPCC1442.16c	0.078	0.105	0.134
SPCC1442.17c	0.045	0.028	0.078
SPCC1450.02	-0.035	0.035	-0.222
SPCC1450.03	-0.233	0.16	0.332
SPCC1450.05c	0.08	-0.07	-0.033
SPCC1450.07c	0.021	-0.001	0.066
SPCC1450.08c	0.036	-0.01	0.054
SPCC1450.09c	0.211	0.135	0.265
SPCC1450.11c	0.107	0.019	0.011
SPCC1450.12	0.052	0.004	0.158
SPCC1450.16c	0.054	-0.069	-0.009
SPCC162.02c	0.039	-0.003	0.144
SPCC162.03	-0.009	0.023	-0.028
SPCC162.04c	0.148	0.163	0.2
SPCC162.06c	0.031	0.08	0.034
SPCC162.10	0.127	0.136	0.18
SPCC162.12	0.574	0.629	0.254
SPCC1672.03c	0.041	-0.043	0.034
SPCC1672.06c	-0.171	-0.351	-0.387
SPCC1672.09	0.026	-0.151	-0.064
SPCC1672.12c	-0.01	-0.011	0.092
SPCC1682.08c	0.036	-0.07	-0.109
SPCC1682.11c	0.058	0.087	-0.104
SPCC1682.12c	0.004	-0.036	-0.145
SPCC1682.13	0.065	0.041	-0.023
SPCC1682.14	0.027	0.034	-0.206
SPCC1682.15	-0.025	0.01	0.021
SPCC16A11.01	0.146	0.082	0.223
SPCC16A11.03c	0.151	0.167	0.185
SPCC16A11.04	0.146	0.152	0.221
SPCC16A11.07	0.077	0.166	-0.005
SPCC16A11.08	0.258	0.187	0.315
SPCC16A11.10c	0.038	0.108	0.29
SPCC16A11.15c	0.168	0.171	0.218
SPCC16A11.16c	0.093	0.108	-0.142
SPCC16C4.01	0.114	0.046	-0.096
SPCC16C4.03	-0.148	-0.132	-0.12
SPCC16C4.04	-0.008	0.114	-0.675
SPCC16C4.06c	0.111	0.012	-0.222
SPCC16C4.09	0.099	0.254	0.423
SPCC16C4.10	0.202	-0.381	-0.582
SPCC16C4.12	0.017	-0.022	-0.027
SPCC16C4.13c	NA	NA	NA
SPCC16C4.20c	0.025	0.04	-0.055
SPCC1742.01	0.031	-0.047	0.115
SPCC1753.02c	0.37	0.367	0.587
SPCC1753.03c	0.114	0.07	0.289
SPCC1795.01c	0.247	0.21	0.165
SPCC1795.02c	0.081	-0.793	-0.627
SPCC1795.09	0.257	0.296	0.369
SPCC18.01c	0.045	-0.063	0.214
SPCC18.02	-0.049	-0.084	-0.096
SPCC18.03	-0.022	-0.092	-0.059
SPCC18.09c	0.093	-0.04	0.069
SPCC18.10	-0.079	-0.851	0.087
SPCC18.13	0.226	0.2	0.12
SPCC18.15	-0.075	0.082	-0.695
SPCC18.17c	-0.072	-0.074	-0.321
SPCC1884.02	-0.156	-0.07	-0.07
SPCC18B5.01c	0.11	-0.13	-0.107
SPCC18B5.03	-0.466	0.362	0.128
SPCC18B5.05c	0.033	-0.014	0.143
SPCC18B5.06	-0.09	0.033	-0.593
SPCC18B5.07c	-0.031	0.014	-0.132
SPCC18B5.09c	0.063	0.153	0.098
SPCC18B5.11c	0.117	0.111	0.285
SPCC1902.01	0.139	0.071	-0.012
SPCC1902.02	0.248	0.143	0.161

SPCC191.05c	0.04	-0.07	0.064
SPCC191.06	0.068	-0.063	0.138
SPCC191.09c	0.002	0	-0.094
SPCC191.10	0.043	-0.114	-0.088
SPCC191.11	0.013	0.011	-0.57
SPCC24B10.02c	0.136	0.142	-0.017
SPCC24B10.03	0.085	0.121	0.217
SPCC24B10.08c	0.343	0.455	0.179
SPCC24B10.09	0.191	0.098	NA
SPCC24B10.10c	0.137	0.098	0.012
SPCC24B10.12	-0.477	-0.018	0.067
SPCC24B10.13	0.188	0.16	0.24
SPCC24B10.15	0.043	0.106	0.185
SPCC24B10.16c	0.19	0.085	0.089
SPCC24B10.17	0.136	0.233	0.203
SPCC24B10.18	-0.043	-0.035	-0.118
SPCC24B10.19c	0.089	0.241	0.262
SPCC24B10.20	0.139	0.119	0.212
SPCC24B10.22	-0.043	-0.121	0.09
SPCC285.04	-0.021	-0.176	-0.064
SPCC285.05	0.051	-0.098	-0.264
SPCC285.10c	0.02	0.075	0.054
SPCC285.13c	0.053	-0.052	-0.68
SPCC285.15c	-0.008	-0.011	-0.504
SPCC285.16c	-0.112	-0.069	0.052
SPCC285.17	0.011	0.028	-0.229
SPCC297.04c	0.018	-0.078	-0.41
SPCC297.05	0.107	-0.129	-0.217
SPCC297.06c	0.055	0.029	0.159
SPCC2H8.02	0.121	-0.041	0.206
SPCC2H8.05c	0.123	0.081	-0.112
SPCC306.02c	-0.002	-0.024	-0.295
SPCC306.04c	-0.784	0.123	-0.655
SPCC306.05c	0.086	-0.084	0.187
SPCC306.07c	0.012	-0.014	-0.241
SPCC306.08c	-0.026	-0.026	-0.312
SPCC31H12.03c	-0.014	0.202	-0.213
SPCC31H12.04c	0.045	-0.003	0.338
SPCC31H12.06	0.048	-0.013	0.2
SPCC320.03	-0.272	-0.016	-1.27
SPCC320.06	-0.145	0.044	-0.291
SPCC320.07c	-0.197	-0.042	-0.513
SPCC320.12	0.003	-0.883	-0.927
SPCC320.14	-0.141	-0.075	-0.575
SPCC330.02	-0.072	-0.108	-0.448
SPCC330.03c	-0.036	-0.113	-0.638
SPCC330.07c	0.047	-0.037	-0.616
SPCC330.11	-0.036	0.057	-0.47
SPCC330.12c	-0.018	0.068	-0.433
SPCC330.14c	-0.082	-0.087	-0.814
SPCC330.19c	-0.077	-0.003	-0.312
SPCC364.01	0.053	0.009	-0.059
SPCC364.02c	-0.01	0.022	-0.005
SPCC364.03	-0.037	-0.114	-0.773
SPCC364.04c	0.015	0.103	-0.018
SPCC364.05	-0.057	0.162	-0.364
SPCC364.06	0.087	0.119	0.188
SPCC364.07	-0.014	-0.033	-0.109
SPCC417.03	0.088	0.14	-0.166
SPCC417.05c	0.063	0.076	0.148
SPCC417.06c	0.098	0.038	0.011
SPCC417.07c	0.023	0.08	-0.477
SPCC417.09c	0.065	0.028	0.072
SPCC417.11c	0.027	0.033	0.071
SPCC417.12	0.001	0	0.187
SPCC4F11.02	-1.042	-0.151	-0.919
SPCC4G3.02	0.045	-0.001	0.128
SPCC4G3.03	0.106	0.03	0.095
SPCC4G3.08	-0.012	0	-0.211
SPCC4G3.09c	0.092	-0.059	-0.28
SPCC4G3.10c	0.07	0.022	-0.065
SPCC4G3.11	0.011	0.017	-0.101
SPCC4G3.12c	-0.002	-0.017	-0.055

SPCC4G3.13c	-0.002	0.177	0.196
SPCC4G3.15c	0.062	-0.019	-0.091
SPCC4G3.17	0.04	0.032	0.151
SPCC4G3.19	0.033	-0.011	0.153
SPCC548.04	-0.165	-0.913	-0.789
SPCC548.05c	0.049	-0.043	-0.624
SPCC548.07c	-0.099	-0.094	-0.092
SPCC553.01c	0	-0.097	-0.32
SPCC553.03	-0.314	-0.176	-1.036
SPCC553.04	0.009	-0.389	-1.044
SPCC553.07c	0.038	0.116	0.129
SPCC553.12c	0.06	-0.056	-0.474
SPCC569.01c	-0.032	0.003	0.014
SPCC569.02c	-0.132	0.026	-0.066
SPCC569.03	-0.037	-0.147	-0.366
SPCC569.04	0.009	-0.066	-0.288
SPCC569.05c	-0.113	0.014	0.037
SPCC569.06	0.026	-0.518	-0.69
SPCC569.07	0.045	-0.132	-0.616
SPCC569.08c	-0.027	0.016	-0.152
SPCC584.01c	0.088	0.156	-0.401
SPCC584.03c	0.129	0.114	0.107
SPCC584.15c	-1.512	0.1	0.275
SPCC584.16c	0.126	0.074	0.127
SPCC594.02c	-0.069	-0.305	-0.949
SPCC594.04c	0.05	0.006	-0.052
SPCC594.05c	-0.222	0.088	-0.431
SPCC594.06c	-0.047	0.18	-0.487
SPCC594.07c	0.064	0.064	0.068
SPCC5E4.05c	-0.003	0.006	0.212
SPCC5E4.10c	-0.047	-0.071	0.162
SPCC613.01	-0.163	-0.013	-0.439
SPCC613.02	-0.14	-0.162	-0.336
SPCC613.03	-0.213	-0.063	-0.202
SPCC613.06	NA	0.372	NA
SPCC613.11c	0.103	0.105	-0.899
SPCC63.03	0.137	0.196	-0.575
SPCC63.04	0.161	0.171	-0.368
SPCC63.06	0.198	0.279	0.432
SPCC63.08c	0.24	0.008	0.181
SPCC63.14	0.047	0.124	0.08
SPCC663.03	0.114	0.075	-0.129
SPCC663.06c	0.03	0	0.292
SPCC663.08c	0.058	0.078	-0.058
SPCC663.09c	-0.001	0.042	0.148
SPCC663.10	0.048	0.026	-0.104
SPCC663.11	0.01	0.17	0.032
SPCC663.13c	0.148	0.114	0.259
SPCC663.14c	0.149	0.109	0.175
SPCC663.15c	0.146	0.086	-0.028
SPCC736.02	-0.174	-0.081	-0.072
SPCC736.04c	-0.09	-0.035	-0.888
SPCC736.08	-0.223	0.109	-0.459
SPCC736.09c	0.043	0.093	0.124
SPCC736.11	0.347	0.348	-0.085
SPCC736.13	0.056	-0.011	-0.246
SPCC737.03c	0.05	0.064	0.021
SPCC737.04	-0.003	-0.025	0.016
SPCC737.05	0.022	-0.067	-0.584
SPCC737.06c	-0.005	-0.079	-0.172
SPCC737.07c	0.015	-0.044	0.125
SPCC737.09c	-0.088	-1.057	-0.956
SPCC74.04	0.082	-0.009	0.069
SPCC74.05	0.042	-0.617	-0.359
SPCC74.06	0.014	-0.113	0.051
SPCC757.02c	-0.197	0.003	-0.743
SPCC757.04	-0.035	-0.058	-0.315
SPCC757.05c	0.015	0.017	0.035
SPCC757.07c	-0.057	0.006	-0.386
SPCC757.09c	-0.25	-0.076	-0.847
SPCC757.11c	-0.018	-0.028	-0.776
SPCC757.12	-0.11	-0.036	-0.186
SPCC757.13	-0.091	0.023	-0.537

SPCC777.02	0.005	0.058	-0.279
SPCC777.03c	0.044	0.058	0.015
SPCC777.04	0.136	0.069	0.263
SPCC777.06c	0.006	-0.047	-0.389
SPCC777.07	0.118	0.161	0.183
SPCC777.08c	-0.072	-0.051	0.013
SPCC777.09c	-0.132	-1.908	-0.155
SPCC777.10c	0.081	-0.384	0.634
SPCC777.12c	0.087	0.035	-0.153
SPCC777.13	0.188	-0.899	-0.638
SPCC777.15	0.109	0.11	0.017
SPCC777.17c	0.014	0.054	0.095
SPCC794.01c	-0.03	-0.052	0.015
SPCC794.02	-0.041	-0.17	-0.495
SPCC794.03	0.01	0.006	-0.366
SPCC794.09c	-0.017	-0.092	-0.205
SPCC794.10	0.017	0.049	0.145
SPCC794.11c	0.144	0.032	-0.176
SPCC794.15	0.001	-0.395	-0.173
SPCC825.02	0.385	0.333	0.147
SPCC825.04c	0.237	0.185	0.265
SPCC825.05c	0.212	0.207	0.239
SPCC895.06	0.438	-0.8	0.1
SPCC895.08c	0.045	0.139	-0.107
SPCC895.09c	0.203	0.201	0.223
SPCC962.04	0.067	0.124	-0.375
SPCC962.05	0.036	-0.128	-0.13
SPCC970.01	-0.102	-0.159	-0.29
SPCC970.02	-0.155	-0.164	-0.248
SPCC970.05	0	-0.151	-0.257
SPCC970.10c	0.039	0.037	0.037
SPCP1E11.11	-0.037	-0.098	-0.457
SPCP20C8.02c	-0.097	-0.031	-0.19
SPCP31B10.02	-0.018	-0.094	-0.131
SPCP31B10.04	-0.048	0.003	-0.17
SPCP31B10.05	0.033	0.028	0.026
SPCP31B10.06	0.029	0.043	0.163
SPCP31B10.07	0.045	0.038	-0.093
SPCPB16A4.02c	0.025	0.078	-0.007
SPCPB16A4.04c	0.125	-0.068	0.011
SPCPB16A4.05c	0.144	0.072	0.196
SPCPB16A4.06c	0.133	0.233	-0.013
SPCPJ732.02c	-0.011	-0.025	0.094

SUPPLEMENTAL TABLE 2

ENRICHMENTS IN GENETIC INTERACTIONS USING THE ANGELI TOOL

GSK3 NEGATIVES

Category_Name	External_ID	GeneSet_Name	over_represented/under_represented	List_Frequency	Background_Frequency	Corrected_pvalue
GO Biological Process	GO:0044260	cellular macromolecule metabolic process	Enriched	55.5555555555556 (105/189)	37.6081233546446 (1000/2659)	8.29E-05
GO Biological Process	GO:0044267	cellular protein metabolic process	Enriched	35.978835978836 (68/189)	20.947724708537 (557/2659)	0.00021497
GO Biological Process	GO:0043170	macromolecule metabolic process	Enriched	55.5555555555556 (105/189)	38.9620157954118 (1036/2659)	0.000562167
GO Biological Process	GO:0019538	protein metabolic process	Enriched	35.978835978836 (68/189)	21.7374952989846 (578/2659)	0.000773615
GO Biological Process	GO:0048519	negative regulation of biological process	Enriched	17.4603174603175 (33/189)	8.19857089131252 (218/2659)	0.00217233
GO Biological Process	GO:0009987	cellular process	Enriched	86.2433862433862 (163/189)	73.5614892816848 (1956/2659)	0.00245955
GO Biological Process	GO:0044238	primary metabolic process	Enriched	64.5502645502645 (122/189)	49.4922903347123 (1316/2659)	0.00291209
GO Biological Process	GO:0048523	negative regulation of cellular process	Enriched	16.4021164021164 (31/189)	7.74727341105679 (206/2659)	0.00384814
GO Biological Process	GO:0016569	covalent chromatin modification	Enriched	7.40740740740741 (14/189)	2.21887927792403 (59/2659)	0.00448272
GO Biological Process	GO:0016570	histone modification	Enriched	7.40740740740741 (14/189)	2.21887927792403 (59/2659)	0.00448272
GO Biological Process	GO:0043162	ubiquitin-dependent protein catabolic process via the multivesicular body sorting pathway	Enriched	3.7037037037037 (7/189)	0.601729973674314 (16/2659)	0.00590137
GO Biological Process	GO:0044237	cellular metabolic process	Enriched	66.1375661375661 (125/189)	52.3505077096653 (1392/2659)	0.00813364
GO Biological Process	GO:0045324	late endosome to vacuole transport	Enriched	4.76190476190476 (9/189)	1.05302745393005 (28/2659)	0.00815249
Phenotypes (FYPO)	FYPO:0000005	abnormal cell morphology	Enriched	40.7407407407407 (77/189)	18.6160210605491 (495/2659)	1.51E-09
Phenotypes (FYPO)	FYPO:0001126	abnormal cell shape	Enriched	38.6243386243386 (73/189)	17.3373448664912 (461/2659)	1.62E-09
Phenotypes (FYPO)	FYPO:0000046	decreased cell population growth	Enriched	29.6296296296296 (56/189)	11.6209101165852 (309/2659)	5.96E-09
Phenotypes (FYPO)	FYPO:0001355	decreased vegetative cell population growth	Enriched	29.1005291005291 (55/189)	11.5080857465212 (306/2659)	1.08E-08
Phenotypes (FYPO)	FYPO:0004325	sensitive to 5-fluorouracil	Enriched	22.2222222222222 (42/189)	7.44640842421963 (198/2659)	1.77E-08
Phenotypes (FYPO)	FYPO:0004638	abnormal cellular physical quality phenotype	Enriched	55.026455026455 (104/189)	31.9292967280933 (849/2659)	1.77E-08
Phenotypes (FYPO)	FYPO:0002177	viable vegetative cell with normal cell morphology	Underrepresented	73.5449735449735 (139/189)	89.6201579541181 (2383/2659)	5.88E-08
Phenotypes (FYPO)	FYPO:0002197	viable vegetative cell with abnormal cell shape	Enriched	31.7460317460317 (60/189)	14.1030462579917 (375/2659)	8.28E-08
Phenotypes (FYPO)	FYPO:0001127	abnormal cell size	Enriched	24.8677248677249 (47/189)	9.96615268898082 (265/2659)	4.48E-07
Phenotypes (FYPO)	FYPO:0001118	abnormal vegetative cell morphology	Enriched	28.042328042328 (53/189)	12.485896953742 (332/2659)	1.47E-06
Phenotypes (FYPO)	FYPO:0002196	abnormal vegetative cell shape	Enriched	26.984126984127 (51/189)	11.6585182399398 (310/2659)	1.47E-06
Phenotypes (FYPO)	FYPO:0001322	abnormal subcellular component during vegetative growth	Enriched	31.2169312169312 (59/189)	15.0808574652125 (401/2659)	3.08E-06
Phenotypes (FYPO)	FYPO:0003037	abnormal cell phenotype	Enriched	57.1428571428571 (108/189)	36.8559608875517 (980/2659)	3.08E-06
Phenotypes (FYPO)	FYPO:0004639	abnormal cellular physical quality phenotype during vegetative growth	Enriched	46.031746031746 (87/189)	26.9650244452802 (717/2659)	3.96E-06
Phenotypes (FYPO)	FYPO:0000287	abnormal subcellular component	Enriched	31.7460317460317 (60/189)	16.0962767957879 (428/2659)	1.39E-05
Phenotypes (FYPO)	FYPO:0000017	elongated cell	Enriched	21.6931216931217 (41/189)	9.02594960511471 (240/2659)	1.79E-05
Phenotypes (FYPO)	FYPO:0000082	decreased cell population growth at high temperature	Enriched	8.99470899470899 (17/189)	2.06844678450545 (55/2659)	4.32E-05
Phenotypes (FYPO)	FYPO:0000708	decreased mating efficiency	Enriched	14.2857142857143 (27/189)	4.77623166603986 (127/2659)	4.32E-05
Phenotypes (FYPO)	FYPO:0000045	abnormal cell population growth	Enriched	73.5449735449735 (139/189)	55.3967657013915 (1473/2659)	5.24E-05
Phenotypes (FYPO)	FYPO:0001356	abnormal vegetative cell population growth	Enriched	73.015873015873 (138/189)	55.0959007145543 (1465/2659)	7.70E-05
Phenotypes (FYPO)	FYPO:0001122	elongated vegetative cell	Enriched	20.1058201058201 (38/189)	8.6874764949229 (231/2659)	0.000108378
Phenotypes (FYPO)	FYPO:0000127	increased sensitivity to chemical during vegetative growth	Enriched	59.7883597883598 (113/189)	41.9330575404287 (1115/2659)	0.000123206
Phenotypes (FYPO)	FYPO:0002683	increased sensitivity to chemical	Enriched	59.7883597883598 (113/189)	42.008273787138 (1117/2659)	0.000123206
Phenotypes (FYPO)	FYPO:0005447	abnormal biological process	Enriched	78.8359788359788 (149/189)	62.3918766453554 (1659/2659)	0.000223981
Phenotypes (FYPO)	FYPO:0000142	gene expression phenotype	Enriched	24.8677248677249 (47/189)	12.3354644603234 (328/2659)	0.000242225
Phenotypes (FYPO)	FYPO:0001362	abnormal cellular process during vegetative growth	Enriched	39.1534391534392 (74/189)	24.0691989469725 (640/2659)	0.000607264
Phenotypes (FYPO)	FYPO:0001407	decreased cell population growth on glucose carbon source	Enriched	15.8730158730159 (30/189)	6.54381346370816 (174/2659)	0.000607264
Phenotypes (FYPO)	FYPO:0001985	abnormal phenotype	Enriched	82.5396825396825 (156/189)	67.619405791651 (1798/2659)	0.000623727
Phenotypes (FYPO)	FYPO:0000139	cell population growth phenotype	Enriched	74.0740740740741 (140/189)	58.1421587062806 (1546/2659)	0.000632547
Phenotypes (FYPO)	FYPO:0001124	normal vegetative cell size	Enriched	14.2857142857143 (27/189)	5.5660022564874 (148/2659)	0.000632547
Phenotypes (FYPO)	FYPO:0001358	vegetative cell population growth phenotype	Enriched	74.0740740740741 (140/189)	58.1421587062806 (1546/2659)	0.000632547
Phenotypes (FYPO)	FYPO:0000628	abnormal cellular process	Enriched	44.973544973545 (85/189)	29.3343362166228 (780/2659)	0.00063415
Phenotypes (FYPO)	FYPO:0000114	cellular process phenotype	Enriched	50.7936507936508 (96/189)	34.8627303497556 (927/2659)	0.000773615
Phenotypes (FYPO)	FYPO:0001353	abnormal cellular component organization during vegetative growth	Enriched	26.4550264550265 (50/189)	14.1406543813464 (376/2659)	0.000773615
Phenotypes (FYPO)	FYPO:0000989	altered level of substance in cell	Enriched	23.8095238095238 (45/189)	12.1474238435502 (323/2659)	0.000860324
Phenotypes (FYPO)	FYPO:0001331	cellular process phenotype during vegetative growth	Enriched	41.2698412698413 (78/189)	26.5137269650244 (705/2659)	0.000980847
Phenotypes (FYPO)	FYPO:0000672	normal cell morphology	Enriched	17.989417989418 (34/189)	8.16096276795788 (217/2659)	0.00102886
Phenotypes (FYPO)	FYPO:0001510	viable vegetative cell, abnormal cell shape, normal cell size	Enriched	13.2275132275132 (25/189)	5.11470477623167 (136/2659)	0.00102886
Phenotypes (FYPO)	FYPO:0000257	normal phenotype	Enriched	48.6772486772487 (92/189)	33.2079729221512 (883/2659)	0.00112944
Phenotypes (FYPO)	FYPO:0001121	vegetative cell, abnormal cell shape, normal cell size	Enriched	13.2275132275132 (25/189)	5.18992102294096 (138/2659)	0.00129228
Phenotypes (FYPO)	FYPO:0001972	abnormal cell separation after cytokinesis resulting in septated cell	Enriched	3.17460317460317 (6/189)	0.338473110191801 (9/2659)	0.00150014
Phenotypes (FYPO)	FYPO:0000059	abnormal mitotic cell cycle	Enriched	21.6931216931217 (41/189)	11.0567882662655 (294/2659)	0.00177603
Phenotypes (FYPO)	FYPO:0000087	sensitive to hydrogen peroxide	Enriched	9.52380952380952 (18/189)	3.1214742384355 (83/2659)	0.00202041
Phenotypes (FYPO)	FYPO:0000300	biological process phenotype	Enriched	80.4232804232804 (152/189)	66.453553967657 (1767/2659)	0.00217233
Phenotypes (FYPO)	FYPO:0000334	cellular component organization phenotype	Enriched	29.6296296296296 (56/189)	17.2621286197819 (459/2659)	0.00217233
Phenotypes (FYPO)	FYPO:0000335	abnormal cellular component organization	Enriched	29.6296296296296 (56/189)	17.2621286197819 (459/2659)	0.00217233
Phenotypes (FYPO)	FYPO:0001312	normal vegetative phenotype	Enriched	30.6878306878307 (58/189)	18.2399398270026 (485/2659)	0.00218946
Phenotypes (FYPO)	FYPO:0001492	viable elongated vegetative cell	Enriched	14.2857142857143 (27/189)	5.94208349003385 (158/2659)	0.0022787
Phenotypes (FYPO)	FYPO:0000834	altered protein level	Enriched	11.1111111111111 (21/189)	4.13689356901091 (110/2659)	0.00255115
Phenotypes (FYPO)	FYPO:0000011	abnormal cell cycle	Enriched	28.042328042328 (53/189)	16.0962767957879 (428/2659)	0.00277996
Phenotypes (FYPO)	FYPO:0000190	abnormal actin cortical patch localization	Enriched	4.23280423280423 (8/189)	0.714554343738247 (19/2659)	0.00294331
Phenotypes (FYPO)	FYPO:0000675	abnormal protein targeting	Enriched	2.11640211640212 (4/189)	0.150432493418578 (4/2659)	0.00327365
Phenotypes (FYPO)	FYPO:0000676	abnormal protein targeting to vacuole	Enriched	2.11640211640212 (4/189)	0.150432493418578 (4/2659)	0.00327365
Phenotypes (FYPO)	FYPO:0001315	normal vegetative cell morphology	Enriched	16.4021164021164 (31/189)	7.70966528770214 (205/2659)	0.00364883
Phenotypes (FYPO)	FYPO:0000801	abnormal actin cytoskeleton organization during vegetative growth	Enriched	5.82010582010582 (11/189)	1.39150056412185 (37/2659)	0.00372868
Phenotypes (FYPO)	FYPO:0000597	abnormal vacuolar transport during vegetative growth	Enriched	2.64550264550265 (5/189)	0.263256863482512 (7/2659)	0.00399138
Phenotypes (FYPO)	FYPO:0000991	decreased level of substance in cell	Enriched	15.8730158730159 (30/189)	7.40880030086499 (197/2659)	0.00430468
Phenotypes (FYPO)	FYPO:0000296	actin cytoskeleton organization phenotype	Enriched	5.82010582010582 (11/189)	1.42910868747649 (38/2659)	0.0044333
Phenotypes (FYPO)	FYPO:0000631	cell cycle phenotype	Enriched	28.042328042328 (53/189)	16.4347499059797 (437/2659)	0.0044333
Phenotypes (FYPO)	FYPO:0001328	abnormal cytoskeleton organization during vegetative growth	Enriched	15.8730158730159 (30/189)	7.44640842421963 (198/2659)	0.0044333
Phenotypes (FYPO)	FYPO:0004803	abnormal actin cytoskeleton organization	Enriched	5.82010582010582 (11/189)	1.42910868747649 (38/2659)	0.0044333
Phenotypes (FYPO)	FYPO:0003578	abnormal vacuolar transport	Enriched	3.17460317460317 (6/189)	0.413689356901091 (11/2659)	0.00450443
Phenotypes (FYPO)	FYPO:0000049	inviable cell	Enriched	13.2275132275132 (25/189)	5.79165099661527 (154/2659)	0.00637915
Phenotypes (FYPO)	FYPO:0000295	cytoskeleton organization phenotype	Enriched	15.8730158730159 (30/189)	7.59684091763821 (202/2659)	0.00813364
Phenotypes (FYPO)	FYPO:0000802	abnormal cytoskeleton organization	Enriched	15.8730158730159 (30/189)	7.59684091763821 (202/2659)	0.00813364
Phenotypes (FYPO)	FYPO:0002401	microtubule bundles present in increased numbers	Enriched	3.17460317460317 (6/189)	0.451297480255735 (12/2659)	0.00813364
Phenotypes (FYPO)	FYPO:0002397	abnormal cytoskeleton	Enriched	13.2275132275132 (25/189)	5.97969161338849 (159/2659)	0.0093629
Phenotypes (FYPO)	FYPO:0000545	decreased protein modification during vegetative growth	Enriched	8.99470899470899 (17/189)	3.30951485520872 (88/2659)	0.00988843
Transcript Features		Relative Pol II occupancy	Higher	1.497481081	1.250757676	0.00217233

GSK3 POSITIVES

Category_Name	External_ID	GeneSet_Name	over_represented/under_represented	List_Frequency	Background_Frequency	Corrected_pvalue
Gene Features		Chromosome 3	Enriched	25.6578947368421 (39/152)	11.959383226777 (318/2659)	0.00130512
Gene Features		Abs. distance from centromere	Lower	1008819.836	1288997.151	0.00650587
Gene Features		Relative distance from telomere	Higher	0.600463613	0.501482262	0.00730652
Gene Features		Relative distance from centromere	Lower	0.399536387	0.498517738	0.00730652
Phenotypes (FYPO)	FYPO:0000127	increased sensitivity to chemical during vegetative growth	Enriched	63.8157894736842 (97/152)	41.9330575404287 (1115/2659)	0.000100758
Phenotypes (FYPO)	FYPO:0002683	increased sensitivity to chemical	Enriched	64.4736842105263 (98/152)	42.008273787138 (1117/2659)	0.000100758
Phenotypes (FYPO)	FYPO:0003559	sensitive to doxorubicin	Enriched	9.86842105263158 (15/152)	1.88040616773223 (50/2659)	0.000122114
Phenotypes (FYPO)	FYPO:0002197	viable vegetative cell with abnormal cell shape	Enriched	30.2631578947368 (46/152)	14.1030462579917 (375/2659)	0.000213777
Phenotypes (FYPO)	FYPO:0001126	abnormal cell shape	Enriched	34.2105263157895 (52/152)	17.3373448664912 (461/2659)	0.000334043
Phenotypes (FYPO)	FYPO:0000005	abnormal cell morphology	Enriched	35.5263157894737 (54/152)	18.6160210605491 (495/2659)	0.000609715
Phenotypes (FYPO)	FYPO:0001355	decreased vegetative cell population growth	Enriched	25.6578947368421 (39/152)	11.5080857465212 (306/2659)	0.000621287
Phenotypes (FYPO)	FYPO:0000046	decreased cell population growth	Enriched	25.6578947368421 (39/152)	11.6209101165852 (309/2659)	0.000658069
Phenotypes (FYPO)	FYPO:0002177	viable vegetative cell with normal cell morphology	Underrepresented	76.3157894736842 (116/152)	89.6201579541181 (2383/2659)	0.000723258
Phenotypes (FYPO)	FYPO:0004638	abnormal cellular physical quality phenotype	Enriched	50.6578947368421 (77/152)	31.9292967280933 (849/2659)	0.000768579
Phenotypes (FYPO)	FYPO:0003037	abnormal cell phenotype	Enriched	55.2631578947368 (84/152)	36.8559608875517 (980/2659)	0.00155645
Phenotypes (FYPO)	FYPO:0001985	abnormal phenotype	Enriched	84.2105263157895 (128/152)	67.619405791651 (1798/2659)	0.00166994
Phenotypes (FYPO)	FYPO:0000102	sensitive to cisplatin	Enriched	9.21052631578947 (14/152)	2.21887927792403 (59/2659)	0.00178933
Phenotypes (FYPO)	FYPO:0000045	abnormal cell population growth	Enriched	73.0263157894737 (111/152)	55.3967657013915 (1473/2659)	0.0025961
Phenotypes (FYPO)	FYPO:0000089	sensitive to methyl methanesulfonate	Enriched	19.0789473684211 (29/152)	8.08574652124859 (215/2659)	0.00366688
Phenotypes (FYPO)	FYPO:0001356	abnormal vegetative cell population growth	Enriched	72.3684210526316 (110/152)	55.0959007145543 (1465/2659)	0.00366688
Phenotypes (FYPO)	FYPO:0005447	abnormal biological process	Enriched	78.2894736842105 (119/152)	62.3918766453554 (1659/2659)	0.00787707

AMK2 NEGATIVES

Category_Name	External_ID	GeneSet_Name	over_represented/under_represented	List_Frequency	Background_Frequency	Corrected_pvalue
Phenotypes (FYPO)	FYPO:0004325	sensitive to 5-fluorouracil	Enriched	16.1971830985916 (46/284)	7.44640842421963 (198/2659)	0.000901861
Phenotypes (FYPO)	FYPO:0000127	increased sensitivity to chemical during vegetative growth	Enriched	55.2816901408451 (157/284)	41.9330575404287 (1115/2659)	0.00362345
Phenotypes (FYPO)	FYPO:0002177	viable vegetative cell with normal cell morphology	Underrepresented	80.6338028169014 (229/284)	89.6201579541181 (2383/2659)	0.00362345
Phenotypes (FYPO)	FYPO:0002346	abnormal chromatin silencing at centromere outer repeat	Enriched	4.22535211267606 (12/284)	0.90259496051147 (24/2659)	0.00362345
Phenotypes (FYPO)	FYPO:0002683	increased sensitivity to chemical	Enriched	55.2816901408451 (157/284)	42.008273787138 (1117/2659)	0.00362345
Phenotypes (FYPO)	FYPO:0003412	decreased chromatin silencing at centromere outer repeat	Enriched	3.87323943661972 (11/284)	0.827378713802181 (22/2659)	0.00551832
Phenotypes (FYPO)	FYPO:0004638	abnormal cellular physical quality phenotype	Enriched	44.0140845070423 (125/284)	31.9292967280933 (849/2659)	0.00906873

AMK2 POSITIVES

Category_Name	External_ID	GeneSet_Name	over_represented/under_represented	List_Frequency	Background_Frequency	Corrected_pvalue
Gene Features		Chromosome 3	Enriched	27.027027027027 (50/185)	11.959383226777 (318/2659)	4.16E-05
Phenotypes (FYPO)	FYPO:0000005	abnormal cell morphology	Enriched	35.1351351351351 (65/185)	18.6160210605491 (495/2659)	0.000102342
Phenotypes (FYPO)	FYPO:0001126	abnormal cell shape	Enriched	33.5135135135135 (62/185)	17.3373448664912 (461/2659)	0.000102342
Phenotypes (FYPO)	FYPO:0002197	viable vegetative cell with abnormal cell shape	Enriched	28.1081081081081 (52/185)	14.1030462579917 (375/2659)	0.000505236
Phenotypes (FYPO)	FYPO:0000127	increased sensitivity to chemical during vegetative growth	Enriched	60 (111/185)	41.9330575404287 (1115/2659)	0.000544827
Phenotypes (FYPO)	FYPO:0002683	increased sensitivity to chemical	Enriched	60 (111/185)	42.008273787138 (1117/2659)	0.000544827
Phenotypes (FYPO)	FYPO:0002177	viable vegetative cell with normal cell morphology	Underrepresented	77.2972972972973 (143/185)	89.6201579541181 (2383/2659)	0.000578115
Phenotypes (FYPO)	FYPO:0001118	abnormal vegetative cell morphology	Enriched	25.4054054054054 (47/185)	12.485896953742 (332/2659)	0.000691118
Phenotypes (FYPO)	FYPO:0003037	abnormal cell phenotype	Enriched	54.0540540540541 (100/185)	36.8559608875517 (980/2659)	0.000915212
Phenotypes (FYPO)	FYPO:0004638	abnormal cellular physical quality phenotype	Enriched	48.6486486486487 (90/185)	31.9292967280933 (849/2659)	0.000915212
Phenotypes (FYPO)	FYPO:0001356	abnormal vegetative cell population growth	Enriched	71.8918918918919 (133/185)	55.0959007145543 (1465/2659)	0.00121181
Phenotypes (FYPO)	FYPO:0000045	abnormal cell population growth	Enriched	71.8918918918919 (133/185)	55.3967657013915 (1473/2659)	0.00162217
Phenotypes (FYPO)	FYPO:0000046	decreased cell population growth	Enriched	23.2432432432432 (43/185)	11.6209101165852 (309/2659)	0.00202183
Phenotypes (FYPO)	FYPO:0000049	inviable cell	Enriched	14.5945945945946 (27/185)	5.79165099661527 (154/2659)	0.00202183
Phenotypes (FYPO)	FYPO:0001355	decreased vegetative cell population growth	Enriched	23.2432432432432 (43/185)	11.5080857465212 (306/2659)	0.00202183
Phenotypes (FYPO)	FYPO:0000139	cell population growth phenotype	Enriched	74.054054054054 (137/185)	58.1421587062806 (1546/2659)	0.00205506
Phenotypes (FYPO)	FYPO:0001358	vegetative cell population growth phenotype	Enriched	74.054054054054 (137/185)	58.1421587062806 (1546/2659)	0.00205506
Phenotypes (FYPO)	FYPO:0005447	abnormal biological process	Enriched	77.2972972972973 (143/185)	62.3918766453554 (1659/2659)	0.00411552
Phenotypes (FYPO)	FYPO:0004639	abnormal cellular physical quality phenotype during vegetative growth	Enriched	41.6216216216216 (77/185)	26.9650244452802 (717/2659)	0.00412989
Phenotypes (FYPO)	FYPO:0001985	abnormal phenotype	Enriched	81.6216216216216 (151/185)	67.619405791651 (1798/2659)	0.00553259
Phenotypes (FYPO)	FYPO:0000085	sensitive to camptothecin	Enriched	12.972972972973 (24/185)	5.26513726965024 (140/2659)	0.00772047
Phenotypes (FYPO)	FYPO:0002196	abnormal vegetative cell shape	Enriched	22.1621621621622 (41/185)	11.6585182399398 (310/2659)	0.00861203
Phenotypes (FYPO)	FYPO:0000088	sensitive to hydroxyurea	Enriched	26.4864864864865 (49/185)	15.0432493418578 (400/2659)	0.00904919
Phenotypes (FYPO)	FYPO:0000304	sensitive to stress during vegetative growth	Enriched	14.0540540540541 (26/185)	6.01729973674314 (160/2659)	0.00981771
Phenotypes (FYPO)	FYPO:0002549	sensitive to stress	Enriched	14.0540540540541 (26/185)	6.01729973674314 (160/2659)	0.00981771

DOUBLE NEGATIVES

Category_Name	External_ID	GeneSet_Name	over_represented/under_represented	List_Frequency	Background_Frequency	Corrected_pvalue
GO Biological Process	GO:0009987	cellular process	Enriched	81.8532818532819 (636/777)	73.5614892816848 (1956/2659)	4.62E-07
GO Biological Process	GO:0009058	biosynthetic process	Enriched	34.3629343629344 (267/777)	25.9496051147048 (690/2659)	6.76E-07
GO Biological Process	GO:0044249	cellular biosynthetic process	Enriched	33.5907335907336 (261/777)	25.3854832643851 (675/2659)	9.40E-07
GO Biological Process	GO:1901576	organic substance biosynthetic process	Enriched	33.5907335907336 (261/777)	25.498307634449 (678/2659)	1.58E-06
GO Biological Process	GO:0071704	organic substance metabolic process	Enriched	60.7464607464607 (472/777)	52.3881158330199 (1393/2659)	1.86E-05
GO Biological Process	GO:0044237	cellular metabolic process	Enriched	60.4890604890605 (470/777)	52.3505077096653 (1392/2659)	3.85E-05
GO Biological Process	GO:0044267	cellular protein metabolic process	Enriched	27.5418275418275 (214/777)	20.947724708537 (557/2659)	6.97E-05
GO Biological Process	GO:0044238	primary metabolic process	Enriched	57.4002574002574 (446/777)	49.4922903347123 (1316/2659)	7.99E-05
GO Biological Process	GO:0010467	gene expression	Enriched	25.0965250965251 (195/777)	18.9921022940955 (505/2659)	0.000181761
GO Biological Process	GO:0019538	protein metabolic process	Enriched	28.0566280566281 (218/777)	21.7374952989846 (578/2659)	0.000218183
GO Biological Process	GO:0002181	cytoplasmic translation	Enriched	7.46460746460746 (58/777)	4.28732606242948 (114/2659)	0.000259033
GO Biological Process	GO:0008152	metabolic process	Enriched	62.5482625482625 (486/777)	55.1711169612636 (1467/2659)	0.000265347
GO Biological Process	GO:0034613	cellular protein localization	Enriched	12.6126126126126 (98/777)	8.3490033847311 (222/2659)	0.000265347
GO Biological Process	GO:0051641	cellular localization	Enriched	18.6615186615187 (145/777)	13.4637081609628 (358/2659)	0.000265347
GO Biological Process	GO:0070727	cellular macromolecule localization	Enriched	12.6126126126126 (98/777)	8.3490033847311 (222/2659)	0.000265347
GO Biological Process	GO:0044260	cellular macromolecule metabolic process	Enriched	44.5302445302445 (346/777)	37.6081233546446 (1000/2659)	0.000750596
GO Biological Process	GO:0034645	cellular macromolecule biosynthetic process	Enriched	22.7799227799228 (177/777)	17.3749529898458 (462/2659)	0.000906809
GO Biological Process	GO:0008104	protein localization	Enriched	12.6126126126126 (98/777)	8.61226024821361 (229/2659)	0.00108161
GO Biological Process	GO:0009059	macromolecule biosynthetic process	Enriched	22.7799227799228 (177/777)	17.4125611132005 (463/2659)	0.0010963
GO Biological Process	GO:0033365	protein localization to organelle	Enriched	6.30630630630631 (49/777)	3.57277171869124 (95/2659)	0.0010963
GO Biological Process	GO:0006412	translation	Enriched	8.23680823680824 (64/777)	5.07709665287702 (135/2659)	0.0012874
GO Biological Process	GO:0043170	macromolecule metabolic process	Enriched	45.5598455598456 (354/777)	38.9620157954118 (1036/2659)	0.0016868
GO Biological Process	GO:0006342	chromatin silencing	Enriched	3.86100386100386 (30/777)	1.99323053779616 (53/2659)	0.00671842
GO Biological Process	GO:0040029	regulation of gene expression, epigenetic	Enriched	3.86100386100386 (30/777)	1.99323053779616 (53/2659)	0.00671842
GO Biological Process	GO:0045814	negative regulation of gene expression, epigenetic	Enriched	3.86100386100386 (30/777)	1.99323053779616 (53/2659)	0.00671842
GO Biological Process	GO:0048523	negative regulation of cellular process	Enriched	11.1969111969112 (87/777)	7.74727341105679 (206/2659)	0.00671842
GO Biological Process	GO:0072594	establishment of protein localization to organelle	Enriched	4.24710424710425 (33/777)	2.29409552463332 (61/2659)	0.00689193
GO Biological Process	GO:0016458	gene silencing	Enriched	3.86100386100386 (30/777)	2.03083866115081 (54/2659)	0.00778347
GO Biological Process	GO:0044699	single-organism process	Enriched	60.3603603603604 (469/777)	54.3813463708161 (1446/2659)	0.00918356
GO Biological Process	GO:0048519	negative regulation of biological process	Enriched	11.5830115830116 (90/777)	8.19857089131252 (218/2659)	0.00918356
GO Biological Process	GO:0080090	regulation of primary metabolic process	Enriched	16.2162162162162 (126/777)	12.1850319669049 (324/2659)	0.00918356
GO Biological Process	GO:0031323	regulation of cellular metabolic process	Enriched	16.3449163449163 (127/777)	12.2978563369688 (327/2659)	0.00919828
Gene Expression		Highly expressed genes	Enriched	12.998712998713 (101/777)	7.78488153441143 (207/2659)	9.40E-07
Gene Expression		Ribosomal protein module 2	Enriched	9.3951093951094 (73/777)	5.2275291462956 (139/2659)	3.67E-06
Gene Expression		Oxidative Stess Cluster 6	Enriched	6.43500643500643 (50/777)	3.27190673185408 (87/2659)	1.86E-05
Gene Expression		Ribosomal protein module Homol D E	Enriched	8.10810810810811 (63/777)	4.58819104926664 (122/2659)	5.80E-05
Gene Expression		Oxidative Stess Cluster 11	Enriched	4.89060489060489 (38/777)	2.36931177134261 (63/2659)	0.000116757
Gene Expression		Oxidative Stess Cluster 9	Enriched	5.66280566280566 (44/777)	2.97104174501692 (79/2659)	0.000265347
Gene Expression		Lowly expressed	Underrepresented	5.79150579150579 (45/777)	9.28920646859722 (247/2659)	0.00725416
Phenotypes (FYPO)	FYPO:0004325	sensitive to 5-fluorouracil	Enriched	13.7709137709138 (107/777)	7.44640842421963 (198/2659)	2.68E-10
Phenotypes (FYPO)	FYPO:0002683	increased sensitivity to chemical	Enriched	50.1930501930502 (390/777)	42.008273787138 (1117/2659)	3.09E-05
Phenotypes (FYPO)	FYPO:0000127	increased sensitivity to chemical during vegetative growth	Enriched	50.0643500643501 (389/777)	41.9330575404287 (1115/2659)	3.62E-05
Phenotypes (FYPO)	FYPO:0000046	decreased cell population growth	Enriched	16.8597168597169 (131/777)	11.6209101165852 (309/2659)	7.74E-05
Phenotypes (FYPO)	FYPO:0001355	decreased vegetative cell population growth	Enriched	16.6023166023166 (129/777)	11.5080857465212 (306/2659)	0.000116757
Phenotypes (FYPO)	FYPO:0001126	abnormal cell shape	Enriched	22.6512226512227 (176/777)	17.3373448664912 (461/2659)	0.0013081
Phenotypes (FYPO)	FYPO:0001356	abnormal vegetative cell population growth	Enriched	61.6473616473616 (479/777)	55.0959007145543 (1465/2659)	0.00240605
Phenotypes (FYPO)	FYPO:0000045	abnormal cell population growth	Enriched	61.7760617760618 (480/777)	55.3967657013915 (1473/2659)	0.00411862
Phenotypes (FYPO)	FYPO:0000005	abnormal cell morphology	Enriched	23.6808236808237 (184/777)	18.6160210605491 (495/2659)	0.00444475
Phenotypes (FYPO)	FYPO:0002177	viable vegetative cell with normal cell morphology	Underrepresented	85.5855855855856 (665/777)	89.6201579541181 (2383/2659)	0.00454276
Phenotypes (FYPO)	FYPO:0004638	abnormal cellular physical quality phenotype	Enriched	37.8378378378378 (294/777)	31.9292967280933 (849/2659)	0.00560873
Phenotypes (FYPO)	FYPO:0000128	auxotrophy	Enriched	2.7027027027027 (21/777)	1.20345994734863 (32/2659)	0.0059207
Phenotypes (FYPO)	FYPO:0004639	abnormal cellular physical quality phenotype during vegetative growth	Enriched	32.5611325611326 (253/777)	26.9650244452802 (717/2659)	0.006109
Phenotypes (FYPO)	FYPO:0000115	sensitive to valproic acid	Enriched	6.56370656370656 (51/777)	4.02406919894697 (107/2659)	0.00715588
Phenotypes (FYPO)	FYPO:0002546	sensitive to trichostatin A	Enriched	2.18790218790219 (17/777)	0.940203083866115 (25/2659)	0.00906616
Phenotypes (FYPO)	FYPO:0000139	cell population growth phenotype	Enriched	64.0926640926641 (498/777)	58.1421587062806 (1546/2659)	0.00918356
Phenotypes (FYPO)	FYPO:0001358	vegetative cell population growth phenotype	Enriched	64.0926640926641 (498/777)	58.1421587062806 (1546/2659)	0.00918356
Protein Features		Homo sapiens orthologs	Enriched	69.3693693693694 (539/777)	61.2260248213614 (1628/2659)	1.86E-05
Protein Features		S.cerevisiae orthologs	Enriched	77.3487773487773 (601/777)	71.4178262504701 (1899/2659)	0.00225769
Transcript Features		Relative Pol II occupancy	Higher	1.463890066	1.188110326	2.41E-07
Transcript Features		mRNA copies per proliferating cell	Higher	11.67486516	5.008942266	4.62E-07
Transcript Features		mRNA level (WT)	Higher	3054.260029	2296.836789	0.000116757
Transcript Features		mRNA copies per quiescent cell	Higher	1.811943871	1.191745684	0.00060158

DOUBLE POSITIVES

NO ENRICHMENT

DOUBLE-SPECIFIC NEGATIVES

Category_Name	External_ID	GeneSet_Name	over_represented/under_represented	List_Frequency	Background_Frequency	Corrected_pvalue
Gene Expression		Highly expressed genes	Enriched	13.508064516129 (67/496)	7.78488153441143 (207/2659)	0.00765039

DOUBLE_SPECIFIC POSITIVES

Category_Name	External_ID	GeneSet_Name	over_represented/under_represented	List_Frequency	Background_Frequency	Corrected_pvalue
Phenotypes (FYPO)	FYPO:0004325	sensitive to 5-fluorouracil	Underrepresented	1.31147540983607 (4/305)	7.44640842421963 (198/2659)	0.00512246
